# Genomic characterization of two metagenome-assembled genomes of *Tropheryma whipplei* from China

**DOI:** 10.3389/fcimb.2022.947486

**Published:** 2022-09-16

**Authors:** Zhongdong Lv, Yong Chen, Houqing Zhou, Zhonglin Chen, Qianru Yao, Jiali Ren, Xianglu Liu, Shuang Liu, Xiaomei Deng, Yingchen Pang, Weijun Chen, Huiling Yang, Ping Xu

**Affiliations:** ^1^ Department of Respiration and Critical Care Medicine, Peking University Shenzhen Hospital, Shenzhen, China; ^2^ Clinical Laboratory of BGI Health, BGI-Shenzhen, Shenzhen, China; ^3^ BGI PathoGenesis Pharmaceutical Technology Co., Ltd, BGI-Shenzhen, Shenzhen, China; ^4^ Department of Laboratory Medicine, Fuwai Hospital Chinese Academy of Medical Sciences, Shenzhen, China; ^5^ Comprehensive Ward, Peking University Shenzhen Hospital, Shenzhen, China; ^6^ Guangdong Provincial Key Laboratory of Research and Development of Natural Drugs, and School of Pharmacy, Guangdong Medical University, Dongguan, China

**Keywords:** tropheryma whipplei, whole-genome analysis, bronchoalveolar lavage (BAL), immunodeficiency – primary, metagenome-assembled genome (MAGs)

## Abstract

Whipple’s disease is a rare chronic systemic disease that affects almost any organ system of the body caused by the intracellular bacterium *Tropheryma whipplei*, which is found ubiquitously in the environment. Sequencing of the *T. whipplei genome* has revealed that it has a reduced genome (0.93 Mbp), a characteristic shared with other intracellular bacteria. Until our research started, 19 *T. whipplei* strains had been sequenced from cultures originated in France, Canada, and Germany. The genome of *T. whipplei* bacterium has not been studied in Asia yet. Here, two metagenome-assembled genomes (MAGs) of *T. whipplei* from China were reconstructed through metagenomic next-generation sequencing (mNGS) and genome binning. We also provided genomic insights into the geographical role and genomic features by analyzing the whole genome. The whole-genome phylogenetic tree was constructed based on single-nucleotide polymorphism (SNP) distance calculations and then grouped by distance similarity. The phylogenetic tree shows inconsistencies with geographic origins, thus suggesting that the variations in geographical origins cannot explain the phylogenetic relationships among the 21 *T. whipplei* strains. The two Chinese strains were closely related to each other, and also found to be related to strains from Germany (*T. whipplei* TW08/27) and France (*T. whipplei* Bcu26 and *T. whipplei* Neuro1). Furthermore, the Average Nucleotide Identity (ANI) matrix also showed no association between geographic origins and genomic similarities. The pan-genome analysis revealed that *T. whipplei* has a closed pan-genome composed of big core-genomes and small accessory genomes, like other intracellular bacteria. By examining the genotypes of the sequenced strains, all 21 *T. whipplei* strains were found to be resistant to fluoroquinolones, due to the genetic mutations in genes *gyrA*, *gyrB*, *parC*, and *parE*. The 21 *T. Whipplei* strains shared the same virulence factors, except for the *alpC* gene, which existed in 7 out of the 21 *T. whipplei* strains. When comparing 21 entire *T. whipplei* pan-genomes from various nations, it was discovered that the bacterium also possessed a closed genome, which was a trait shared by intracellular pathogens.

## Introduction

The Gram-positive bacterium *Tropheryma whipplei* causes a rare multi-systematic infectious disease known as Whipple’s disease, which has clinical manifestations of fever, weight loss, lymphadenopathy, and polyarthritis, as well as cardiac manifestations and central nervous system complications (Ratliff et al., 1984; Durand et al., 1997). The “intestinal lipodystrophy” disease was first reported by George H. Whipple in 1907 and renamed Whipple’s disease by Black-Schaffer in 1949 (Black-Schaffer, 1949). A bacterial infection was long believed to be responsible for Whipple’s disease until in 1961 when the real origin of the disease was discovered by electron microscopy. Researchers detected bacterial inclusions in macrophages and monocytes, which together constituted the predominant infected cell types of this disease (Yardley and Hendrix, 1961). Researchers discovered that *T. whipplei* is a fastidious bacterium and extremely difficult to culture. The bacterium was first successfully isolated and grown in inactivated human mononuclear phagocytes by Schoedon in 1997, but culture could not be reproduced (Schoedon et al., 1997). In 2000, Raoult isolated the bacterium *T. whipplei* Twist from the aortic valve of a patient with endocarditis and propagated it in a human fibroblast cell line with the doubling time of 18 days; however, it could not be cultivated in the absence of living eukaryotic cells (Raoult et al., 2000). Subsequently, Raoult found a doubling time of 32 to 34 h for *T. whipplei* Twist strain, when propagated in the MRC5 cell culture system (Masselot et al., 2003). However, this doubling time was still longer than the slowly growing bacterium *M. tuberculosis* (14.3 *h* to 24 h) (James et al., 2000). In 2001, the *T. whipplei* strain Twist-Marseille was proposed by La Scola as the type strain of a new species of a new genus. The detailed characterization of the bacterium was described and deposited at the Collection Nationale de Culture de Micro-organisms de l’Institut Pasteur, Paris, France (La Scola et al., 2001).

The cultivation of *T. whipplei* made it possible to reveal the genome. So far, 19 strains of *T. whipplei* had been successfully cultured, and their genomes were sequenced by Bentley et al. (2003); Raoult et al. (2003), and Wetzstein (2017). Similar to other intracellular bacteria with rudimentary metabolic functions, genomic sequencing revealed that *T. whipplei* had a reduced genome (*T. whipplei* Twist, 0.93 Mbp). *T. whipplei* is ubiquitous in the environment, and it can lead to widespread colonization of the lower respiratory tract of healthy children and adults (Dickson et al., 2015). The bacterium could result in endocarditis, gastrointestinal infection, neurological complications and pulmonary infection, but the incident of Whipple’s disease is very rare. There were some reports of pulmonary infection caused by *T. whipplei*, which were diagnosed by metagenomic next-generation sequencing (Li et al., 2021; Zhu et al., 2021). *T. whipplei* is a commensal bacterium that only causes Whipple’s disease in a small number of individuals. Our understanding of the genome of *T. whipplei* from China is still not clear, due to the harsh cultivation conditions and the lengthy culture period. Metagenomic next-generation sequencing (mNGS) is a high-throughput sequencing technique that sequences all nucleic acids in a sample simultaneously *in situ*, which includes *T. whipplei* DNA if an individual had been infected by Whipple’s disease. The possible clinical mNGS applications are tremendous, including diagnosis of infectious diseases, outbreak tracking, infection control surveillance, and new pathogen discovery, among many other purposes. This emerging approach is an unbiased hypothesis-free diagnostic tool. It has been widely applied to guiding infectious disease management and developing treatment strategies. Next-generation sequencing (NGS) makes it possible to analyze genomes precisely and accurately, which aids the detection of single-nucleotide polymorphisms (SNPs) on a large scale. A metagenome-assembled genome *T. whipplei* shenzhen1 was assembly based on binned metagenome data of bronchoalveolar lavage samples from 26 non-immunodeficient patients, and metagenome-assembled genome *T. whipplei* shenzhen2 genome was assembled based on metagenome data of a bronchoalveolar lavage sample from an immunodeficient patient. All bronchoalveolar lavage samples from patients were collected by Peking University Shenzhen Hospital in China. The purpose of this study is to answer whether there was any difference between *T. whipplei* shenzhen1 and *T. whipplei* shenzhen2 genome, as well as the 19 genomes of *T. whipplei* that had been deposited in the NCBI database, including the two completed genomes of *T. whipplei* Twist and *T. whipplei* TW08/27 (Bentley et al., 2003; Raoult et al., 2003; Wetzstein, 2017).

## Materials and methods

### Sample collection and DNA extraction

Bronchoalveolar lavage fluid (BALF) samples were collected from one immunodeficient patient and 26 non-immunodeficient patients who had been admitted to Shenzhen Peking University Shenzhen Hospital with pulmonary infection. We subsequently extracted total genomic DNA from each BALF sample. Briefly, 0.6 ml of BALF and 250 μl of 0.5-mm glass beads in a 1.5-ml microcentrifuge tube were attached to a horizontal platform on a vortex mixer and agitated vigorously at 2,800–3,200 rpm for 30 min. Then, 7.2 μl of lysozyme was added for wall-breaking reaction. A 0.3-ml sample was separated into a new 1.5-ml microcentrifuge tube and DNA was extracted using the TIANamp Micro DNA Kit (DP316, TIANGEN BIOTECH) according to the manufacturer’s recommendation.

### DNA library construction and sequencing

The extracted DNA obtained in the previous step was first fragmented to yield 300-bp fragments using enzymatic digestion (RM0434, BGI Wuhan Biotechnology). To construct the DNA library, fragmented DNA was further end-repaired, ligated to adapters, and amplified using PCR with the PMseq high-throughput gene detection kit for infectious pathogens (combined probe anchored polymerization sequencing method, BGI-Shenzhen, China, RM0438), according to the manufacturer’s instruction. Based on the qualified double-strand DNA library, the single-stranded circular DNA library was then generated through DNA denaturation and circularization. Then, DNA nanoballs (DNBs) were formed by rolling circle amplification (RCA) using a universal kit for sequencing reaction (Combinatorial Probe-Anchor Synthesis, BGI-Shenzhen, China, RM0170). DNBs were qualified by the Qubit^®^ ssDNA Assay Kit (Thermo Fisher Scientific) and were further sequenced by the MGISEQ-2000 platform (MGI, China).

### Metagenome-assembly genome of *T. whipplei*

A quality control step was conducted on the metagenomic sequencing data by using the fastp tool to filter out low-quality and contaminated reads. By utilizing the Burrows-Wheeler alignment algorithm, the human DNA reads that aligned to human reference genome HG19 were eliminated, and only microbe reads were reserved. The remaining data were mapped and classified by aligning the reads to genomes of bacteria, fungi, viruses, and parasites from the Pathogens Metagenomics Database (PMDB). The classification reference databases were downloaded from NCBI (ftp://ftp.ncbi.nlm.nih.gov/genomes/). *T. whipplei* was found in all 27 BALF samples, according to the mNGS diagnostic results. The *T. whipplei* shenzhen1 genome was assembled by combining metagenomic sequencing data of BALF samples from 26 non-immunodeficient patients with low-quality reads, and host genomes were removed. The *T. whipplei* shenzhen2 genome was assembled using metagenomic sequencing data of BALF sample from one immunodeficient patient. Briefly, the reads were subjected to *de novo* metagenomic assembly through metaSPAdes, and contigs shorter than 1,000 nt were discarded from further processing. Reads were mapped to contigs using Bowtie2, and the mapping output was used for contig binning through MetaBAT2. Lastly, putative genomes were subjected to quality control to generate the final set of reconstructed draft genomes. Two metagenome assembled genomes of *T. whipplei* from China were obtained. **Table 1** summarizes the origin and the genome information of *T. whipplei* strains from China in this study and the 19 *T. whipplei* genomes reported by other researchers.

**Table 1:** Summary of 21 *T. whipplei* strains studied in this study.

Strain	Genome size (Mb)	GC-content (%)	Level	Accession	Geographical origin
Twist	0.927303	46.3	Chromosome	AE014184.1	Canada
TW08/27	0.925938	46.3	Chromosome	BX072543.1	Germany
SLOW2	0.927621	46.3	Scaffold	HG794425.1	France
NEURO1	0.927567	46.3	Scaffold	NZ_HG421449.1	Germany
DIG7	0.927564	46.3	Scaffold	HG794427.1	France
DIG9	0.880115	46.4	Contig	CAUY000000000	France
DIG10	0.927515	46.4	Scaffold	HG794428.1	Germany
ART1	0.927575	46.3	Scaffold	HG424698.1	France
NEURO14	0.885853	46.4	Contig	CAUR000000000	Germany
DIG15	0.927582	46.3	Scaffold	HG794423.1	Germany
DIGMUSC17	0.884564	46.4	Contig	CAVA000000000	France
NEURO20	0.883582	46.3	Contig	CAUX000000000	Germany
DIGADP25	0.883649	46.3	Contig	CAUW000000000	France
TWBCU26	0.880271	46.4	Contig	CAVB000000000	France
ENDO27	0.927598	46.4	Scaffold	HG794429.1	France
SALI28	0.927465	46.4	Scaffold	HG794430.1	France
ART29	0.927595	46.3	Scaffold	HG794431.1	France
PNEUMO30	0.927553	46.4	Scaffold	HG794432.1	France
ENDO32	0.927567	46.4	Scaffold	HG794424.1	France
shenzhen1	0.883965	46.4	Scaffold	JAMYWJ000000000	China
shenzhen2	0.899012	46.6	Scaffold	JAMYWK000000000	China

Genome size, GC-contents, genome assembly level, accession codes, and geographical origin are depicted as well.

### Whole-genome phylogenetic tree analysis

Conducting whole-genome phylogenetic tree for microbial pathogens is a powerful approach that assists scientists to gain a better understanding of how species have evolved while explaining the similarities and differences among species. A wide range of genomic features can be observed across the entire genome derived from mNGS. These characteristics make phylogeny building extremely accurate. To discover the evolution of the 21 *T. whipplei* strains’ origin from different countries, the phylogenetic trees were constructed based on SNP datasets.

### Characterization of core-genome and pan-genome

To characterize the core- and pan-genomes of the 21 strains of the *T. whipplei* genome, the PEPPAN pipeline, which can construct pan-genomes from thousands of genetically diverse bacterial genomes, was applied (Zhou et al., 2020). Genes presented in all 21 *T. whipplei* genomes were considered to be the core-genome, genes presented in more than 95% but not in all strains were defined as the softcore genes, genes presented in 15%–95% of the genomes were considered the shell genes, while genes presented in lower than 15% of the genomes were defined as the cloud genes. The pan-genome analysis of the 21 *T. whipplei* was performed by Anvi`o workflow to display the genome, which is an advanced analysis and visualization platform that offers both automated and/or user-specified characterization of metagenomic assembly genomes. (Eren et al., 2015)

### Average nucleotide identity analysis

Average nucleotide identity analysis is a useful approach to compare genetic relatedness among prokaryotic genomes The whole-genome average nucleotide identity (ANI) values for the 21 *T. whipplei* strains belonging to diverse geographic origins (Goris et al., 2007; Jain et al., 2018) were calculated to assess the genome similarities by using the FastANI v.0.1.3 software, which produces accurate ANI estimates and is a more efficient approach than alignment (e.g., BLAST)-based approaches.

### Identification of antibiotic-resistant genes and virulence genes

To comprehend the virulence genes of the *T. whipplei* pathogens, 21 *T. whipplei* genomes were annotated with the Prokka annotation pipeline (ProkkaAnnotation v.3.2.1), and we then utilized the BLAST search tool for all known VF-related genes found in the virulence factor database (VFDB) (Chen et al., 2005). At the same time, the virulence factors were compared between *T. whipplei* strains originating from different countries. Reliable virulence genes were confirmed if the sequence identities were greater than 80% and the query coverages were greater than 80%, in which the values are used as benchmarks for virulence factor detection. The aligned amino acid sequences of GyrA, GyrB, ParC, and ParE for 21 *T. whipplei* strains were submitted to ESPript 3 to perform the sequence similarities, respectively (Robert and Gouet, 2014).

For the 21 genomes of *T. whipplei*, antibiotic resistance genes were predicted by aligning the hybrid assembled sequences in the CARD database using RGI v4.2.2 (Resistance Gene Identifier). Subsequently, the genes and the subsequent protein sequence were predicted using Prodigal. During this step, sequences that had an identity of less than 75% or a length coverage of less than 50% with the resistant genes denoted in the database were removed (Jia et al., 2017). The antibiotic resistance genes of *T. whipplei* were predicted using RAST (Rapid Annotation using Subsystem Technology, https://rast.nmpdr.org/), which is an automated service for annotating bacterial genomes.

## Results

The pathogen diagnosis of severe respiratory diseases was carried out using clinical metagenomic next-generation sequencing on 27 BALF samples from one immunodeficient patient and 26 non-immunodeficient patients who were admitted to Shenzhen Peking University Hospital. The metagenome-assembled genome of *T. whipplei* shenzhen1 was constructed based on the binned metagenomes of 26 BALF samples from non-immunodeficient patients, whereas the metagenome-assembled genome *T. whipplei* shenzhen2 genome was built based on one metagenome of a BALF sample from an immunodeficient patient.

### Whole-genome phylogenetic tree analysis

**Figure 1:**
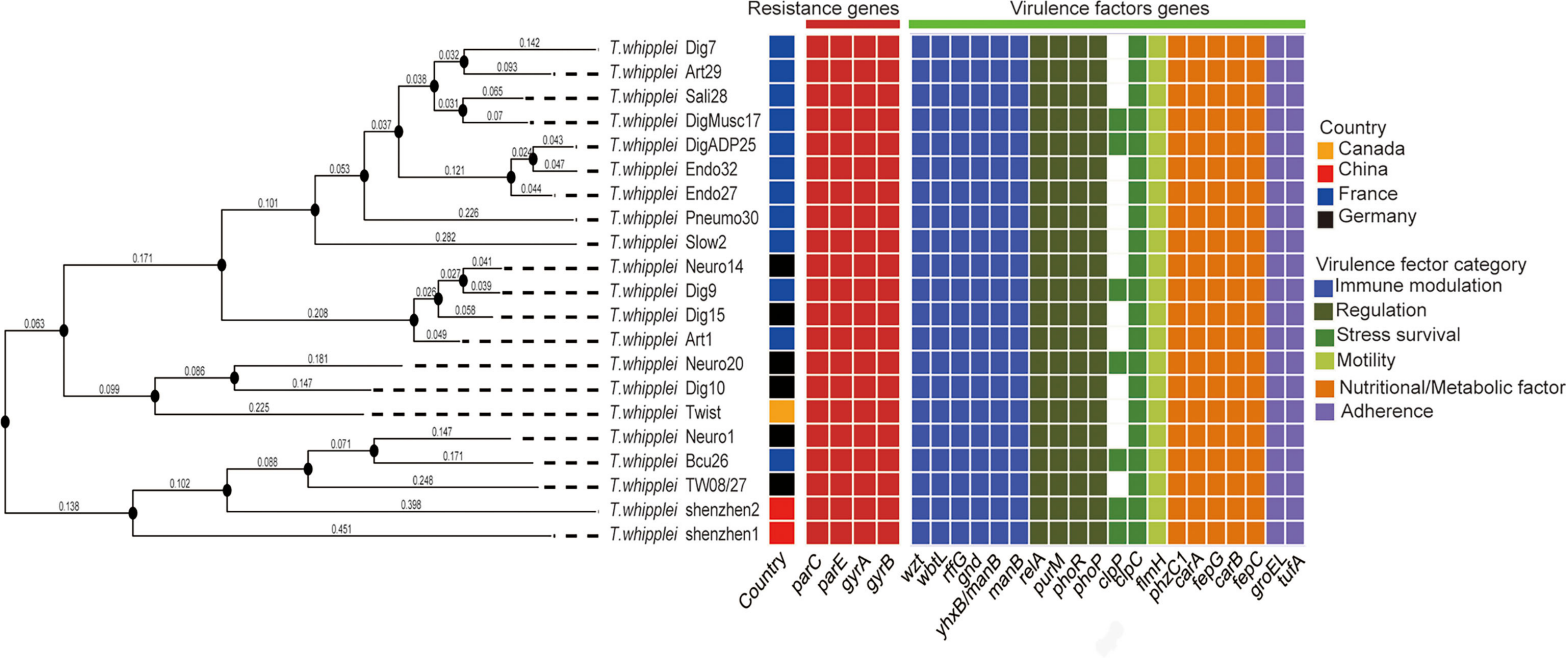
Whole-genome phylogenetic tree of 21 *T. whipplei* was constructed by single-nucleotide polymorphism (SNP) distance calculation, and the geographical origin of strains was obtained. The heatmaps represent the predicted resistance genes of chromosomal mutations known to confer resistance (gyrase and topoisomerase mutations conferring fluoroquinolone resistance) and the predicted virulence factors of *T. whipplei*.

The whole-genome phylogenetic tree constructed with 21 strains of the *T. whipplei* genome comprises three major clades, while one clade contains two subclades (**Figure 1**). The two strains *T. whipplei* shenzhen1 and *T. whipplei* shenzhen2 from China, the two strains *T. whipplei* TW08/27 and *T. whipplei* Meuro1 from Germany, and the one strain *T. whipplei* Bcu26 from France are located within the same subclade in the phylogenetic tree. Furthermore, the clades and subclades in the phylogenetic tree are not correlated with the geographical origins of *T. whipplei*.

### Characterization of core-genome and pan-genome

**Figure 2:**
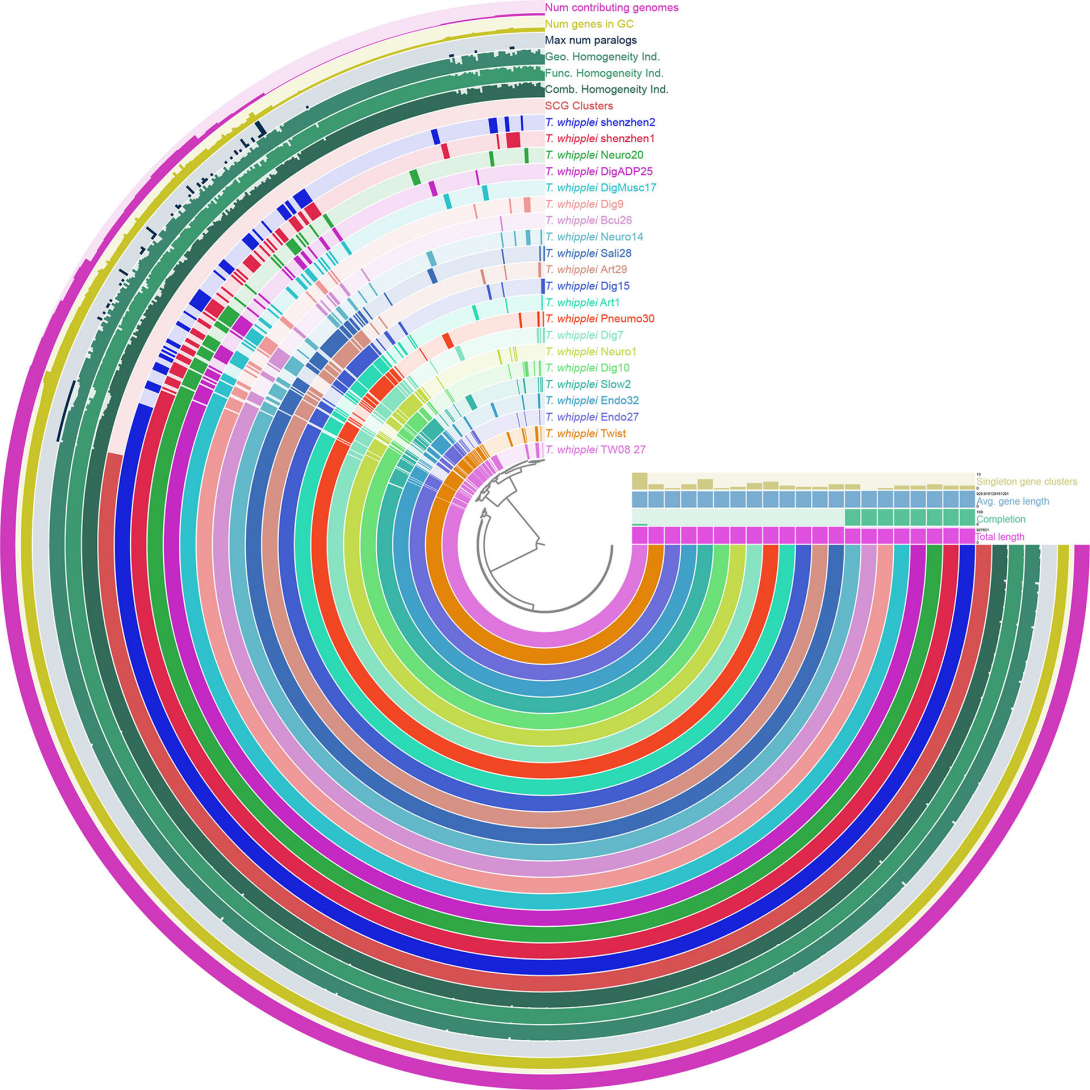
Anvi’o representation of the pan-genome of *T. whipplei shenzhen1*, *T. whippleishenzhen2*, and 19 other *T. whipplei* based on the presence/absence of gene clusters. The inner layers represent individual genomes organized by their phylogenetic relationships as indicated by the dendrogram. The first 21 layers represent each genome. Circle bars represent the occurrence of gene clusters in each genome, and the dark colors indicate the existence of the gene cluster. The subsequent seven layers correspond to various statistics related to the analysis, i.e., single-copy core gene clusters, combine homogeneity index, functional homogeneity index, geometric homogeneity index, maximum number of paralogs per gene cluster, number of genes per gene cluster, and the number of contributing genomes per gene cluster.

To uncover the view of *T. whipplei* genome contents, the core-genome and pan-genome for 21 *T. whipplei* assembled genomes were calculated. The pan-genome of *T. whipplei* contained 977 genes, which were predicted by using the PEPPAN pipeline. The core-genome contained 809 genes that are common to all 21 strains of *T. whipplei*, and the core-genome accounts for 82.8% of the pan-genome, indicating that *T. whipplei* has a closed pan-genome. An additional 22 genes belong to the softcore genes, 58 genes form the shell genes, and 88 genes belong to the cloud genes. The pan-genome of 21 *T. whipplei* genomes was further analyzed by Anvi`o pan-genomic pipeline to visualize the pan-genome (**Figure 2**). As **Figure 2** shows, the single-copy core genes occupy a major part of the pan-genome. Thirteen singleton gene clusters were found from *T. whipplei* TW08/27, which is the maximum number of singleton gene clusters of *T. whipplei*. We could thus hypothesize that *T. whipplei* is a strictly intracellular living organism that has a reduced genome, and no significant horizontal gene transfer has taken place in the past.

### Average nucleotide identity analysis

**Figure 3:**
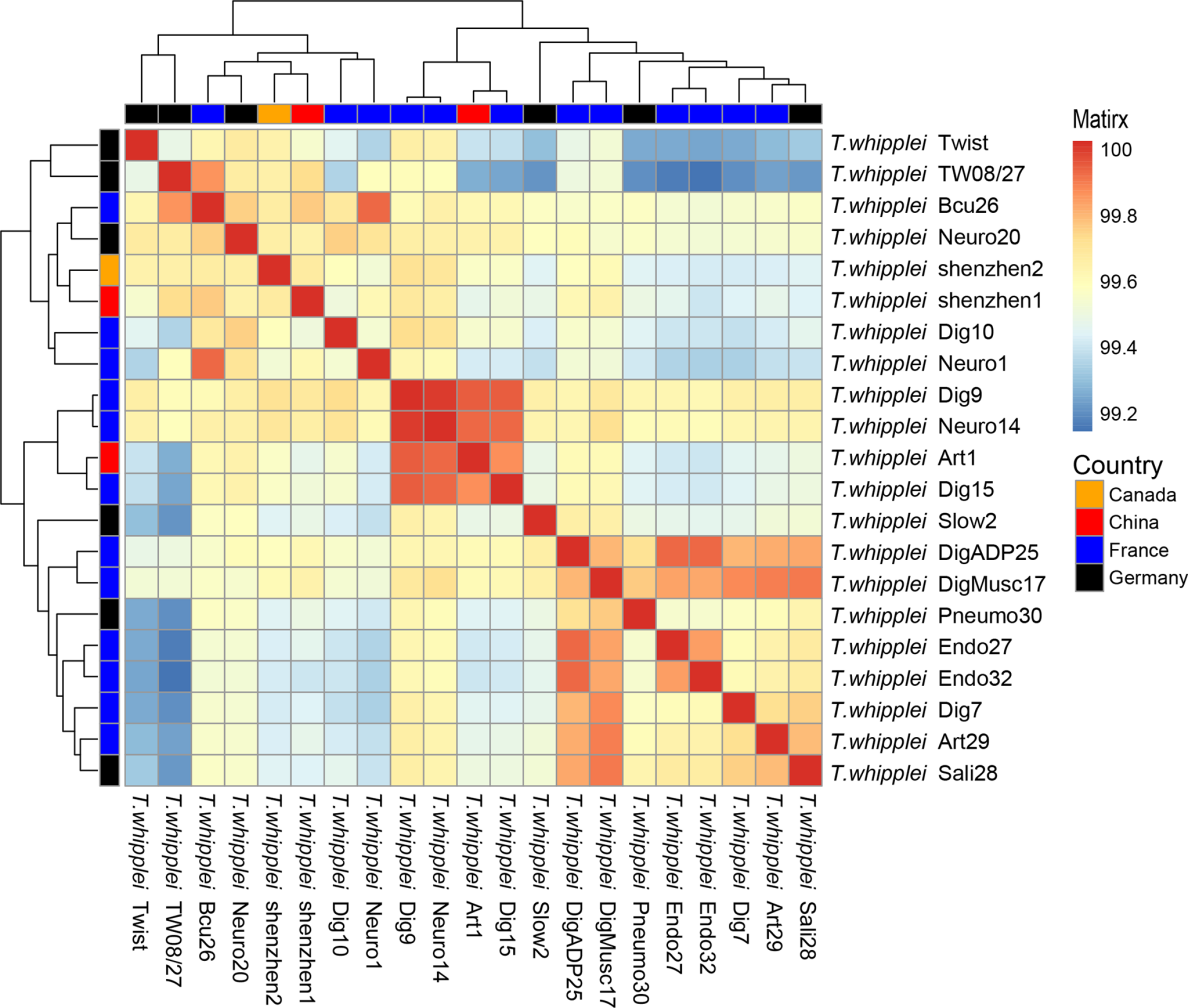
Heatmap of Average Nucleotide Identity (ANI) values for 21 whole genomes of *T. whipplei* strains from the different geographical origins (orange, Canada; red, China; blue, France; black, Germany).

The whole-genome ANI lay between 99.11% and 99.98%, whereas the median ANI of all sequenced strains is 99.54% when all 21 *T. whipplei* genomes were compared with each other. The genomes with maximum and minimum ANI values for *T. whipplei* shenzhen1 are *T. whipplei* Bcu26 (99.74%) and *T. whipplei* strain Endo32 (99.38%) originating from France. The genome with maximum ANI for *T. whipplei* strain shenzhen2 is *T. whipplei* Bcu26. At the same time, the ANI between the two *T. whipplei* strains from China is 99.65%. As **Figure 3** illustrates, the average nucleotide identity value calculated between all pairs of strains shows no discernible difference associated with geographical origins.

### Identification of antibiotic-resistant genetic determinants and virulence genes

**Figure 4:**
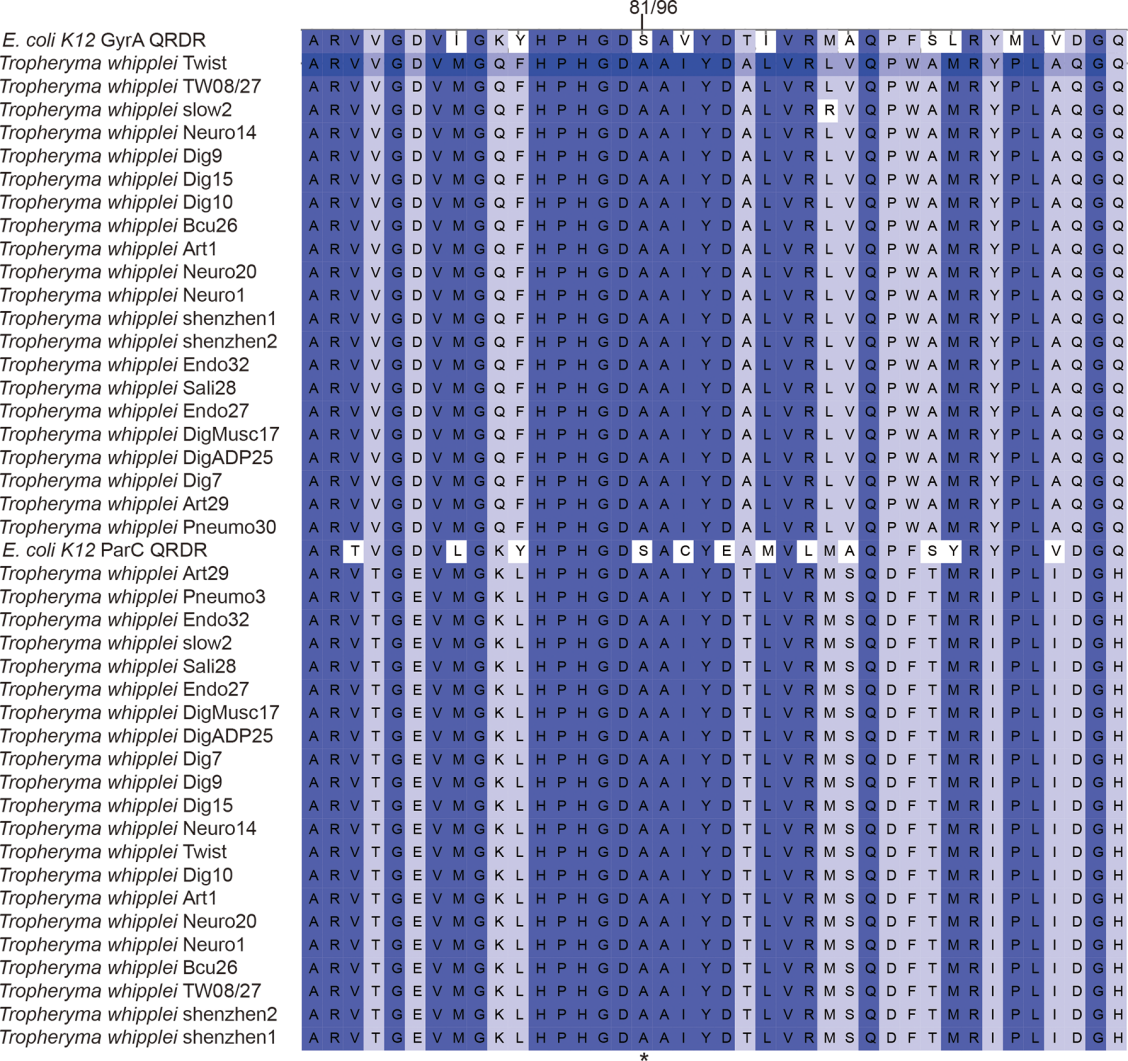
Alignment of amino acid sequences of GyrA and ParC QRDRs from the 21 *T. whipplei* strains and *E. coli* K-12. Numbers refer to the amino acid positions in the *T. whipplei* GyrA and ParC sequence.

Submitting the 21 strains of *T. whipplei* sequenced genomes to the Resistance Gene Identifier failed to detect any antibiotic resistance genes. However, after utilizing RAST-Annotation, the specific mutation in the genes for DNA gyrase (*gyrA* and *gyrB*) and topoisomerase IV (*parC* and *parE*) leading to the genotypic antibiotic resistance to fluoroquinolones was found in *T. whipplei*. Some reports proved that *gyrA, gyrB, parC*, and *parE* gene mutations induce resistance to fluoroquinolones, due to the altered structures of the target proteins of fluoroquinolones (Gonzalez et al., 1998; Pantel et al., 2012; Johnning et al., 2015; Chien et al., 2016). Alignment of the amino acid sequences of GyrA, GyrB, ParC, and ParE of the 21 *T. whipplei* strains showed that these genes are highly conserved within the species (**Figures S1**–**S4**). The *T. whipplei* GyrA and ParC quinolone resistance-determining regions (QRDRs) are shown in **Figures S1**, **S3**. According to Didier Raoult’s report (Masselot et al., 2003), the *T. whipplei* GyrA QRDR extends from the alanine at position 65 to the glutamine at position 104, and the ParC QRDR extends from the alanine at position 80 to the histidine a position 119. The amino acid sequences of GyrA and ParC QRDR from the 21 *T. whipplei* strains and that of *Escherichia coli* were aligned, and the Ser-to-Ala mutation is indicated in **Figure 4**. The positions of this mutation are at positions 81 and 96, respectively. Alanines at these positions have previously been associated with increased fluoroquinolone resistance in *T. whipplei, E. coli*, and *Mycobacteria* (Cullen et al., 1989; Yagupsky et al., 1990; Masselot et al., 2003). The *T. whipplei* GyrB and ParE QRDR were identified by aligning with known homologous QRDR sequences of *Mycobacterium fortuitum* H37Rv (Cole et al., 1998). The GyrB QRDR of *T. whipplei* extends from the serine at position 474 to the glutamine at position 512. The ParE QRDR of *T. whipplei* extends from the alanine at position 488 to alanine at position 526. The mutations that likely to cause the fluoroquinolone resistance (e.g., Asp to Asn) were not detected in the GyrB and ParE QRDR regions.

The virulence factors of *T. whipplei* were investigated through the virulence factor database, which harbors information of bacterial virulence factors from various known pathogens. Twenty virulence factors had been predicted to play a role in the pathogenesis of *T. whipplei* (**Figure 1**). These include six bacterial VF categories: adherence (*groEL* and *ufa*), immune modulation (*wzt*, *wbtL, rffG, gnd, manB*, and *yhxB/manB*), nutritional/metabolic factor (*phzC*1*, carA, fepG, carB*, and *fepC*), regulation (*relA, purM, phoR*, and *phoP*), stress survival (*clpC* and *clpP*), and motility (*flmH*), but further studies need to be done to understand the role of these virulence factors. Among the 21 *T. whipplei* strains, 19 virulence factors are shared among the strains, while the *clpC* gene is found in 7 out of 21 *T. whipplei* strains. It encodes a general stress protein belonging to the HSP-100/Clp family, which promotes intracellular bacteria *Listeria monocytogenes* to escape from the macrophage phagocytosis (Rouquette et al., 1998).

## Discussion

*T. whipplei* is a fastidious bacterium that is difficult to culture, and not until after 2000 could the researchers successfully culture this bacterium in the human fibroblast cell line in the laboratory (Raoult et al., 2000). There is increasing evidence to suggest that the predominant reservoir of *T. whipplei* is found in humans, as *T. whipplei* is known to be viable in the human respiratory tract, fecal, and saliva samples. It also suggests that *T. whipplei* might be transmitted through both fecal–oral and oral–oral routes (La Scola et al., 2011; Pightling et al., 2018). In previous studies, 19 *T. whipplei* strains were isolated from various specimens, including the aortic valve, small intestine biopsy, mesenterial lymph node, and bronchoalveolar lavage cutaneous biopsy, in which the pathogens were cultured and the genomes were sequenced (Bentley et al., 2003; Raoult et al., 2003; Wetzstein, 2017). The cultivation of *T. whipplei* requires living eukaryotic cells, while the bacterium has a very slow replication with a doubling time of 18 days, which severely impedes routine culture-based diagnostics and genomic analysis. On the other hand, obtaining the bacterial whole genome is significant to understanding the properties of this pathogen, including antibiotic resistance, molecular epidemiology, and pathogenic virulence. Utilizing whole-genome sequence analyses could supplement epidemiological studies and trace back evidence for epidemic regulations (Pightling et al., 2018). Moreover, it could help identify sources of pathogens during disease outbreaks. In this study, we report two genome sequences of *T. whipplei* shenzhen1 and *T. whipplei* shenzhen2, which were assembled using clinical metagenomic sequencing, without the need to conduct long-term bacterial cultivation.

In comparative genomics, the genetic content of 21 *T. whipplei* was compared to each other. The antibiotic resistance genes and virulence genes were predicted, and the phylogenetic relationships between the strains were determined. Interestingly, even strains of *T. whipplei* that originate from different geographical regions have close relationships, including the isolate *T. whipplei* shenzhen2 from an immunodeficient patient. *T. whipplei* is an intracellular bacterium that has a closed pan-genome, suggested by investigating the core and pan-genomes of *T. whipplei*. It has a limited capacity to acquire foreign genes likely due to its limited horizontal gene transfer mechanisms. As shown by the whole-genome phylogenetic tree and the ANI matrix, there is no correlation between the strains and their geographical origins. All *T. whipplei* strains exhibit genotypic resistance to fluoroquinolones, due to mutations found in the *gyrA*, *gyrB*, *parC*, and *parE* genes in the RAST annotation. Mutations in the quinolone resistance-determining region (QRDR) of *gyrA*, *gyrB*, *parC*, and *parE* leading to reduce susceptibility to fluoroquinolone have been reported in many bacterial species (Gonzalez et al., 1998; Pantel et al., 2012; Johnning et al., 2015; Chien et al., 2016). All *T. whipplei* strains exhibit genotypic resistance to fluoroquinolones, due to the GyrA and ParC QRDR with an alanine residue at positions 81 and 96 (Masselot et al., 2003). The QRDRs of *T. whipplei* GyrB and ParE were defined as codons 474 to 512 in GyrB, and 488 to 526 in the ParE, but the specific amino acid relative to fluoroquinolone resistance was not discovered. Although the mechanisms of quinolone resistance of GyrB and ParE have not been fully investigated, we still hypothesize that *T. whipplei* has a natural resistance to fluoroquinolones.

In conclusion, we have obtained two metagenome-assembled genomes of *T. whipplei* from China using metagenomic next-generation sequencing (mNGS). The 21 *T. whipplei* strains share highly similar genomic characteristics despite originating from different countries. Aided by the mNGS culture-independent characterization of pathogens, we therefore propose that clinical mNGS could be considered as an approach to obtain and analyze genomic information for difficult or “unculturable” microorganisms.

## Data availability statement

The datasets presented in this study can be found in online repositories. The name of the repository and accession number can be found below: NCBI; PRJNA831609.

## Ethics statement

The studies involving human participants were reviewed and approved by The Peking University Shenzhen Hospital Ethics Committee. The patients/participants provided their written informed consent to participate in this study.

## Author contributions

PX, YH participated in study conception and design. ZL, SL, XLL, XMD enrolled and managed patients, CY, CZ and YQ carried out collection and assembly of data. ZL, CY were involved in data analysis and interpretation. PX, YH, ZL, CY prepared the manuscript and manuscript figures. PX, YH and ZL edited, critically read, and revised the manuscript. All authors contributed to the article and approved the submitted version.

## Funding

This study was supported by the National Key Research and Development Program of China (2019YFC1200500 and 2019YFC1200501), the Shenzhen Science and Technology Innovation Commission Foundation (grant no. JCYJ20190809103203711), the Shenzhen Science and Technology Innovation Commission foundation (grant no. JCYJ20210324105411031), the Fund of “Sanming” Project of Medicine in Shenzhen (no. SZSM201811096), the Shenzhen High-level Hospital Construction Fund (LCYJ2021022), and the Shenzhen High-level Hospital Construction Fund Discipline Construction Project of Guangdong Medical University.

## Conflict of interest

Authors YC, ZC, QY, JR and WC are employed by BGI.

The remaining authors declare that the research was conducted in the absence of any commercial or financial relationships that could be construed as a potential conflict of interest.

## Publisher’s note

All claims expressed in this article are solely those of the authors and do not necessarily represent those of their affiliated organizations, or those of the publisher, the editors and the reviewers. Any product that may be evaluated in this article, or claim that may be made by its manufacturer, is not guaranteed or endorsed by the publisher.
