# Supplementary material for: Genomic characterization of two metagenome-assembled genomes of Tropheryma whipplei from China
Source: Front Cell Infect Microbiol. 2022 Sep 16;12:947486. doi: 10.3389/fcimb.2022.947486 (PMC9523146; doi:10.3389/fcimb.2022.947486)
Supplement: Supplementary file 1 [file DataSheet_1.pdf]

```
                                .1          10          20          30          40          50          60          70
Tropheryma whipplei Twist       MSEQNINQVDLREEMQRSYLDYAMSVIIGRALPDVRDGLKPVHRRVLYAMYKGGYRPEKAFSKCARVVGDVMGQF
Tropheryma whipplei TW08/27     MSEQNINQVDLREEMQRSYLDYAMSVIIGRALPDVRDGLKPVHRRVLYAMYKGGYRPEKAFSKCARVVGDVMGQF
Tropheryma whipplei slow2       MSEQNINQVDLREEMQRSYLDYAMSVIIGRALPDVRDGLKPVHRRVLYAMYKGGYRPEKAFSKCARVVGDVMGQF
Tropheryma whipplei Neuro14     MSEQNINQVDLREEMQRSYLDYAMSVIIGRALPDVRDGLKPVHRRVLYAMYKGGYRPEKAFSKCARVVGDVMGQF
Tropheryma whipplei Dig9        MSEQNINQVDLREEMQRSYLDYAMSVIIGRALPDVRDGLKPVHRRVLYAMYKGGYRPEKAFSKCARVVGDVMGQF
Tropheryma whipplei Dig15       MSEQNINQVDLREEMQRSYLDYAMSVIIGRALPDVRDGLKPVHRRVLYAMYKGGYRPEKAFSKCARVVGDVMGQF
Tropheryma whipplei Dig10       MSEQNINQVDLREEMQRSYLDYAMSVIIGRALPDVRDGLKPVHRRVLYAMYKGGYRPEKAFSKCARVVGDVMGQF
Tropheryma whipplei Bcu26       MSEQNINQVDLREEMQRSYLDYAMSVIIGRALPDVRDGLKPVHRRVLYAMYKGGYRPEKAFSKCARVVGDVMGQF
Tropheryma whipplei Art1        MSEQNINQVDLREEMQRSYLDYAMSVIIGRALPDVRDGLKPVHRRVLYAMYKGGYRPEKAFSKCARVVGDVMGQF
Tropheryma whipplei Neuro20     MSEQNINQVDLREEMQRSYLDYAMSVIIGRALPDVRDGLKPVHRRVLYAMYKGGYRPEKAFSKCARVVGDVMGQF
Tropheryma whipplei Neuro1      MSEQNINQVDLREEMQRSYLDYAMSVIIGRALPDVRDGLKPVHRRVLYAMYKGGYRPEKAFSKCARVVGDVMGQF
Tropheryma whippleishenzhen1    MSEQNINQVDLREEMQRSYLDYAMSVIIGRALPDVRDGLKPVHRRVLYAMYKGGYRPEKAFSKCARVVGDVMGQF
Tropheryma whipplei shenzhen2   MSEQNINQVDLREEMQRSYLDYAMSVIIGRALPDVRDGLKPVHRRVLYAMYKGGYRPEKAFSKCARVVGDVMGQF
Tropheryma whipplei Endo32      MSEQNINQVDLREEMQRSYLDYAMSVIIGRALPDVRDGLKPVHRRVLYAMYKGGYRPEKAFSKCARVVGDVMGQF
Tropheryma whipplei Sali28      MSEQNINQVDLREEMQRSYLDYAMSVIIGRALPDVRDGLKPVHRRVLYAMYKGGYRPEKAFSKCARVVGDVMGQF
Tropheryma whipplei Endo27      MSEQNINQVDLREEMQRSYLDYAMSVIIGRALPDVRDGLKPVHRRVLYAMYKGGYRPEKAFSKCARVVGDVMGQF
Tropheryma whipplei DigMusc17   MSEQNINQVDLREEMQRSYLDYAMSVIIGRALPDVRDGLKPVHRRVLYAMYKGGYRPEKAFSKCARVVGDVMGQF
Tropheryma whipplei DigADP25    MSEQNINQVDLREEMQRSYLDYAMSVIIGRALPDVRDGLKPVHRRVLYAMYKGGYRPEKAFSKCARVVGDVMGQF
Tropheryma whipplei Dig7        MSEQNINQVDLREEMQRSYLDYAMSVIIGRALPDVRDGLKPVHRRVLYAMYKGGYRPEKAFSKCARVVGDVMGQF
Tropheryma whipplei Art29       MSEQNINQVDLREEMQRSYLDYAMSVIIGRALPDVRDGLKPVHRRVLYAMYKGGYRPEKAFSKCARVVGDVMGQF
Tropheryma whipplei Pneumo30    MSEQNINQVDLREEMQRSYLDYAMSVIIGRALPDVRDGLKPVHRRVLYAMYKGGYRPEKAFSKCARVVGDVMGQF
```

Quinolone

```
                                    80          90          100         110         120         130         140         150
Tropheryma whipplei Twist       HPHGDAAIYDALVRLVQPWAMRYPLAQGQGNFGSPGNDGAAAPRYTETKMATLAIEMVRDIDEDTVDFSPNYDGE
Tropheryma whipplei TW08/27     HPHGDAAIYDALVRLVQPWAMRYPLAQGQGNFGSPGNDGAAAPRYTETKMATLAIEMVRDIDEDTVDFSPNYDGE
Tropheryma whipplei slow2       HPHGDAAIYDALVRRVQPWAMRYPLAQGQGNFGSPGNDGAAAPRYTETKMATLAIEMVRDIDEDTVDFSPNYDGE
Tropheryma whipplei Neuro14     HPHGDAAIYDALVRLVQPWAMRYPLAQGQGNFGSPGNDGAAAPRYTETKMATLAIEMVRDIDEDTVDFSPNYDGE
Tropheryma whipplei Dig9        HPHGDAAIYDALVRLVQPWAMRYPLAQGQGNFGSPGNDGAAAPRYTETKMATLAIEMVRDIDEDTVDFSPNYDGE
Tropheryma whipplei Dig15       HPHGDAAIYDALVRLVQPWAMRYPLAQGQGNFGSPGNDGAAAPRYTETKMATLAIEMVRDIDEDTVDFSPNYDGE
Tropheryma whipplei Dig10       HPHGDAAIYDALVRLVQPWAMRYPLAQGQGNFGSPGNDGAAAPRYTETKMATLAIEMVRDIDEDTVDFSPNYDGE
Tropheryma whipplei Bcu26       HPHGDAAIYDALVRLVQPWAMRYPLAQGQGNFGSPGNDGAAAPRYTETKMATLAIEMVRDIDEDTVDFSPNYDGE
Tropheryma whipplei Art1        HPHGDAAIYDALVRLVQPWAMRYPLAQGQGNFGSPGNDGAAAPRYTETKMATLAIEMVRDIDEDTVDFSPNYDGE
Tropheryma whipplei Neuro20     HPHGDAAIYDALVRLVQPWAMRYPLAQGQGNFGSPGNDGAAAPRYTETKMATLAIEMVRDIDEDTVDFSPNYDGE
Tropheryma whipplei Neuro1      HPHGDAAIYDALVRLVQPWAMRYPLAQGQGNFGSPGNDGAAAPRYTETKMATLAIEMVRDIDEDTVDFSPNYDGE
Tropheryma whipplei shenzhen1   HPHGDAAIYDALVRLVQPWAMRYPLAQGQGNFGSPGNDGAAAPRYTETKMATLAIEMVRDIDEDTVDFSPNYDGE
Tropheryma whipplei shenzhen2   HPHGDAAIYDALVRLVQPWAMRYPLAQGQGNFGSPGNDGAAAPRYTETKMATLAIEMVRDIDEDTVDFSPNYDGE
Tropheryma whipplei Endo32      HPHGDAAIYDALVRLVQPWAMRYPLAQGQGNFGSPGNDGAAAPRYTETKMATLAIEMVRDIDEDTVDFSPNYDGE
Tropheryma whipplei Sali28      HPHGDAAIYDALVRLVQPWAMRYPLAQGQGNFGSPGNDGAAAPRYTETKMATLAIEMVRDIDEDTVDFSPNYDGE
Tropheryma whipplei Endo27      HPHGDAAIYDALVRLVQPWAMRYPLAQGQGNFGSPGNDGAAAPRYTETKMATLAIEMVRDIDEDTVDFSPNYDGE
Tropheryma whipplei DigMusc17   HPHGDAAIYDALVRLVQPWAMRYPLAQGQGNFGSPGNDGAAAPRYTETKMATLAIEMVRDIDEDTVDFSPNYDGE
Tropheryma whipplei DigADP25    HPHGDAAIYDALVRLVQPWAMRYPLAQGQGNFGSPGNDGAAAPRYTETKMATLAIEMVRDIDEDTVDFSPNYDGE
Tropheryma whipplei Dig7        HPHGDAAIYDALVRLVQPWAMRYPLAQGQGNFGSPGNDGAAAPRYTETKMATLAIEMVRDIDEDTVDFSPNYDGE
Tropheryma whipplei Art29       HPHGDAAIYDALVRLVQPWAMRYPLAQGQGNFGSPGNDGAAAPRYTETKMATLAIEMVRDIDEDTVDFSPNYDGE
Tropheryma whipplei Pneumo30    HPHGDAAIYDALVRLVQPWAMRYPLAQGQGNFGSPGNDGAAAPRYTETKMATLAIEMVRDIDEDTVDFSPNYDGE
```

Resistance-Determining Regions

```
                                        160         170         180         190         200         210         220
Tropheryma whipplei Twist       SVEPTVMPCRFPNLLVNGSVGIAVGMATNIPPHNLAEVSAGALWCLENPDATDEELTEALLERIKGPDFPTGATV
Tropheryma whipplei TW08/27     SVEPTVMPCRFPNLLVNGSVGIAVGMATNIPPHNLAEVSAGALWCLENPDATDEELTEALLERIKGPDFPTGATV
Tropheryma whipplei slow2       SVEPTVMPCRFPNLLVNGSVGIAVGMATNIPPHNLAEVSAGALWCLENPDATDEELTEALLERIKGPDFPTGATV
Tropheryma whipplei Neuro14     SVEPTVMPCRFPNLLVNGSVGIAVGMATNIPPHNLAEVSAGALWCLENPDATDEELTEALLERIKGPDFPTGATV
Tropheryma whipplei Dig9        SVEPTVMPCRFPNLLVNGSVGIAVGMATNIPPHNLAEVSAGALWCLENPDATDEELTEALLERIKGPDFPTGATV
Tropheryma whipplei Dig15       SVEPTVMPCRFPNLLVNGSVGIAVGMATNIPPHNLAEVSAGALWCLENPDATDEELTEALLERIKGPDFPTGATV
Tropheryma whipplei Dig10       SVEPTVMPCRFPNLLVNGSVGIAVGMATNIPPHNLAEVSAGALWCLENPDATDEELTEALLERIKGPDFPTGATV
Tropheryma whipplei Bcu26       SVEPTVMPCRFPNLLVNGSVGIAVGMATNIPPHNLAEVSAGALWCLENPDATDEELTEALLERIKGPDFPTGATV
Tropheryma whipplei Art1        SVEPTVMPCRFPNLLVNGSVGIAVGMATNIPPHNLAEVSAGALWCLENPDATDEELTEALLERIKGPDFPTGATV
Tropheryma whipplei Neuro20     SVEPTVMPCRFPNLLVNGSVGIAVGMATNIPPHNLAEVSAGALWCLENPDATDEELTEALLERIKGPDFPTGATV
Tropheryma whipplei Neuro1      SVEPTVMPCRFPNLLVNGSVGIAVGMATNIPPHNLAEVSAGALWCLENPDATDEELTEALLERIKGPDFPTGATV
Tropheryma whipplei shenzhen1   SVEPTVMPCRFPNLLVNGSVGIAVGMATNIPPHNLAEVSAGALWCLENPDATDEELTEALLERIKGPDFPTGATV
Tropheryma whipplei shenzhen2   SVEPTVMPCRFPNLLVNGSVGIAVGMATNIPPHNLAEVSAGALWCLENPDATDEELTEALLERIKGPDFPTGATV
Tropheryma whipplei Endo32      SVEPTVMPCRFPNLLVNGSVGIAVGMATNIPPHNLAEVSAGALWCLENPDATDEELTEALLERIKGPDFPTGATV
Tropheryma whipplei Sali28      SVEPTVMPCRFPNLLVNGSVGIAVGMATNIPPHNLAEVSAGALWCLENPDATDEELTEALLERIKGPDFPTGATV
Tropheryma whipplei Endo27      SVEPTVMPCRFPNLLVNGSVGIAVGMATNIPPHNLAEVSAGALWCLENPDATDEELTEALLERIKGPDFPTGATV
Tropheryma whipplei DigMusc17   SVEPTVMPCRFPNLLVNGSVGIAVGMATNIPPHNLAEVSAGALWCLENPDATDEELTEALLERIKGPDFPTGATV
Tropheryma whipplei DigADP25    SVEPTVMPCRFPNLLVNGSVGIAVGMATNIPPHNLAEVSAGALWCLENPDATDEELTEALLERIKGPDFPTGATV
Tropheryma whipplei Dig7        SVEPTVMPCRFPNLLVNGSVGIAVGMATNIPPHNLAEVSAGALWCLENPDATDEELTEALLERIKGPDFPTGATV
Tropheryma whipplei Art29       SVEPTVMPCRFPNLLVNGSVGIAVGMATNIPPHNLAEVSAGALWCLENPDATDEELTEALLERIKGPDFPTGATV
Tropheryma whipplei Pneumo30    SVEPTVMPCRFPNLLVNGSVGIAVGMATNIPPHNLAEVSAGALWCLENPDATDEELTEALLERIKGPDFPTGATV
```

```
                                  230         240         250         260         270         280         290         300
Tropheryma whipplei Twist       LDTGGIKDAYLTGRGSVVMRGVVDIEEIDGRTCLVVRELPYQVNPDNLALKIASLVKENKLSGIADIRDETSGRT
Tropheryma whipplei TW08/27     LDTGGIKDAYLTGRGSVVMRGVVDIEEIDGRTCLVVRELPYQVNPDNLALKIASLVKENKLSGIADIRDETSGRT
Tropheryma whipplei slow2       LDTGGIKDAYLTGRGSVVMRGVVDIEEIDGRTCLVVRELPYQVNPDNLALKIASLVKENKLSGIADIRDETSGRT
Tropheryma whipplei Neuro14     LDTGGIKDAYLTGRGSVVMRGVVDIEEIDGRTCLVVRELPYQVNPDNLALKIASLVKENKLSGIADIRDETSGRT
Tropheryma whipplei Dig9        LDTGGIKDAYLTGRGSVVMRGVVDIEEIDGRTCLVVRELPYQVNPDNLALKIASLVKENKLSGIADIRDETSGRT
Tropheryma whipplei Dig15       LDTGGIKDAYLTGRGSVVMRGVVDIEEIDGRTCLVVRELPYQVNPDNLALKIASLVKENKLSGIADIRDETSGRT
Tropheryma whipplei Dig10       LDTGGIKDAYLTGRGSVVMRGVVDIEEIDGRTCLVVRELPYQVNPDNLALKIASLVKENKLSGIADIRDETSGRT
Tropheryma whipplei Bcu26       LDTGGIKDAYLTGRGSVVMRGVVDIEEIDGRTCLVVRELPYQVNPDNLALKIASLVKENKLSGIADIRDETSGRT
Tropheryma whipplei Art1        LDTGGIKDAYLTGRGSVVMRGVVDIEEIDGRTCLVVRELPYQVNPDNLALKIASLVKENKLSGIADIRDETSGRT
Tropheryma whipplei Neuro20     LDTGGIKDAYLTGRGSVVMRGVVDIEEIDGRTCLVVRELPYQVNPDNLALKIASLVKENKLSGIADIRDETSGRT
Tropheryma whipplei Neuro1      LDTGGIKDAYLTGRGSVVMRGVVDIEEIDGRTCLVVRELPYQVNPDNLALKIASLVKENKLSGIADIRDETSGRT
Tropheryma whipplei shenzhen1   LDTGGIKDAYLTGRGSVVMRGVVDIEEIDGRTCLVVRELPYQVNPDNLALKIASLVKENKLSGIADIRDETSGRT
Tropheryma whipplei shenzhen2   LDTGGIRDAYLTGRGSVVMRGVVDIEEIDGRTCLVVRELPYQVNPDNLALKIASLVKENKLSGIADIRDETSGRT
Tropheryma whipplei Endo32      LDTGGIRDAYLTGRGSVVMRGVVDIEEIDGRTCLVVRELPYQVNPDNLALKIASLVKENKLSGIADIRDETSGRT
Tropheryma whipplei Sali28      LDTGGIRDAYLTGRGSVVMRGVVDIEEIDGRTCLVVRELPYQVNPDNLALKIASLVKENKLSGIADIRDETSGRT
Tropheryma whipplei Endo27      LDTGGIRDAYLTGRGSVVMRGVVDIEEIDGRTCLVVRELPYQVNPDNLALKIASLVKENKLSGIADIRDETSGRT
Tropheryma whipplei DigMusc17   LDTGGIRDAYLTGRGSVVMRGVVDIEEIDGRTCLVVRELPYQVNPDNLALKIASLVKENKLSGIADIRDETSGRT
Tropheryma whipplei DigADP25    LDTGGIRDAYLTGRGSVVMRGVVDIEEIDGRTCLVVRELPYQVNPDNLALKIASLVKENKLSGIADIRDETSGRT
Tropheryma whipplei Dig7        LDTGGIRDAYLTGRGSVVMRGVVDIEEIDGRTCLVVRELPYQVNPDNLALKIASLVKENKLSGIADIRDETSGRT
Tropheryma whipplei Art29       LDTGGIRDAYLTGRGSVVMRGVVDIEEIDGRTCLVVRELPYQVNPDNLALKIASLVKENKLSGIADIRDETSGRT
Tropheryma whipplei Pneumo30    LDTGGIRDAYLTGRGSVVMRGVVDIEEIDGRTCLVVRELPYQVNPDNLALKIASLVKENKLSGIADIRDETSGRT
```

```
                                   310       320       330       340       350       360       370
Tropheryma whipplei Twist         GQRLIIVLKRDAVARVVLNSLYKHTQLQENFGVNMLAIVDGAPRTLSLCSFIRLWVEHQIEVIVRRTTFRLARAE
Tropheryma whipplei TW08/27       GQRLIIVLKRDAVARVVLNSLYKHTQLQENFGVNMLAIVDGAPRTLSLCSFIRLWVEHQIEVIVRRTTFRLARAE
Tropheryma whipplei slow2         GQRLIIVLKRDAVARVVLNSLYKHTQLQENFGVNMLAIVDGAPRTLSLCSFIRLWVEHQIEVIVRRTTFRLARAE
Tropheryma whipplei Neuro14       GQRLIIVLKRDAVARVVLNSLYKHTQLQENFGVNMLAIVDGAPRTLSLCSFIRLWVEHQIEVIVRRTTFRLARAE
Tropheryma whipplei Dig9          GQRLIIVLKRDAVARVVLNSLYKHTQLQENFGVNMLAIVDGAPRTLSLCSFIRLWVEHQIEVIVRRTTFRLARAE
Tropheryma whipplei Dig15         GQRLIIVLKRDAVARVVLNSLYKHTQLQENFGVNMLAIVDGAPRTLSLCSFIRLWVEHQIEVIVRRTTFRLARAE
Tropheryma whipplei Dig10         GQRLIIVLKRDAVARVVLNSLYKHTQLQENFGVNMLAIVDGAPRTLSLCSFIRLWVEHQIEVIVRRTTFRLARAE
Tropheryma whipplei Bcu26         GQRLIIVLKRDAVARVVLNSLYKHTQLQENFGVNMLAIVDGAPRTLSLCSFIRLWVEHQIEVIVRRTTFRLARAE
Tropheryma whipplei Art1          GQRLIIVLKRDAVARVVLNSLYKHTQLQENFGVNMLAIVDGAPRTLSLCSFIRLWVEHQIEVIVRRTTFRLARAE
Tropheryma whipplei Neuro20       GQRLIIVLKRDAVARVVLNSLYKHTQLQENFGVNMLAIVDGAPRTLSLCSFIRLWVEHQIEVIVRRTTFRLARAE
Tropheryma whipplei Neuro1        GQRLIIVLKRDAVARVVLNSLYKHTQLQENFGVNMLAIVDGAPRTLSLCSFIRLWVEHQIEVIVRRTTFRLARAE
Tropheryma whipplei shenzhen1     GQRLIIVLKRDAVARVVLNSLYKHTQLQENFGVNMLAIVDGAPRTLSLCSFIRLWVEHQIEVIVRRTTFRLARAE
Tropheryma whipplei shenzhen2     GQRLIIVLKRDAVARVVLNSLYKHTQLQENFGVNMLAIVDGAPRTLSLCSFIRLWVEHQIEVIVRRTTFRLARAE
Tropheryma whipplei Endo32        GQRLIIVLKRDAVARVVLNSLYKHTQLQENFGVNMLAIVDGAPRTLSLCSFIRLWVEHQIEVIVRRTTFRLARAE
Tropheryma whipplei Sali28        GQRLIIVLKRDAVARVVLNSLYKHTQLQENFGVNMLAIVDGAPRTLSLCSFIRLWVEHQIEVIVRRTTFRLARAE
Tropheryma whipplei Endo27        GQRLIIVLKRDAVARVVLNSLYKHTQLQENFGVNMLAIVDGAPRTLSLCSFIRLWVEHQIEVIVRRTTFRLARAE
Tropheryma whipplei DigMusc17     GQRLIIVLKRDAVARVVLNSLYKHTQLQENFGVNMLAIVDGAPRTLSLCSFIRLWVEHQIEVIVRRTTFRLARAE
Tropheryma whipplei DigADP25      GQRLIIVLKRDAVARVVLNSLYKHTQLQENFGVNMLAIVDGAPRTLSLCSFIRLWVEHQIEVIVRRTTFRLARAE
Tropheryma whipplei Dig7          GQRLIIVLKRDAVARVVLNSLYKHTQLQENFGVNMLAIVDGAPRTLSLCSFIRLWVEHQIEVIVRRTTFRLARAE
Tropheryma whipplei Art29         GQRLIIVLKRDAVARVVLNSLYKHTQLQENFGVNMLAIVDGAPRTLSLCSFIRLWVEHQIEVIVRRTTFRLARAE
Tropheryma whipplei Pneumo30      GQRLIIVLKRDAVARVVLNSLYKHTQLQENFGVNMLAIVDGAPRTLSLCSFIRLWVEHQIEVIVRRTTFRLARAE

                              380       390       400       410       420       430       440       450
Tropheryma whipplei Twist         QRAHILRAYLRALDQIDEVIQLIRSSKSAEEAQSNLMALLKIDETQARAILDLQLRRLAALERQRIISEAREIEE
Tropheryma whipplei TW08/27       QRAHILRAYLRALDQIDEVIQLIRSSKSAEEAQSNLMALLKIDETQARAILDLQLRRLAALERQRIISEAREIEE
Tropheryma whipplei slow2         QRAHILRAYLRALDQIDEVIQLIRSSKSAEEAQSNLMALLKIDETQARAILDLQLRRLAALERQRIISEAREIEE
Tropheryma whipplei Neuro14       QRAHILRAYLRALDQIDEVIQLIRSSKSAEEAQSNLMALLKIDETQARAILDLQLRRLAALERQRIISEAREIEE
Tropheryma whipplei Dig9          QRAHILRAYLRALDQIDEVIQLIRSSKSAEEAQSNLMALLKIDETQARAILDLQLRRLAALERQRIISEAREIEE
Tropheryma whipplei Dig15         QRAHILRAYLRALDQIDEVIQLIRSSKSAEEAQSNLMALLKIDETQARAILDLQLRRLAALERQRIISEAREIEE
Tropheryma whipplei Dig10         QRAHILRAYLRALDQIDEVIQLIRSSKSAEEAQSNLMALLKIDETQARAILDLQLRRLAALERQRIISEAREIEE
Tropheryma whipplei Bcu26         QRAHILRAYLRALDQIDEVIQLIRSSKSAEEAQSNLMALLKIDETQARAILDLQLRRLAALERQRIISEAREIEE
Tropheryma whipplei Art1          QRAHILRAYLRALDQIDEVIQLIRSSKSAEEAQSNLMALLKIDETQARAILDLQLRRLAALERQRIISEAREIEE
Tropheryma whipplei Neuro20       QRAHILRAYLRALDQIDEVIQLIRSSKSAEEAQSNLMALLKIDETQARAILDLQLRRLAALERQRIISEAREIEE
Tropheryma whipplei Neuro1        QRAHILRAYLRALDQIDEVIQLIRSSKSAEEAQSNLMALLKIDETQARAILDLQLRRLAALERQRIISEAREIEE
Tropheryma whipplei shenzhen1     QRAHILRAYLKALDQIDEVIQLIRSSKSAEEAQSNLMALLKIDETQARAILDLQLRRLAALERQRIISEAREIEE
Tropheryma whipplei shenzhen2     QRAHILRAYLKALDQIDEVIQLIRSSKSAEGAQSNLMALLKIDETQARAILDLQLRRLAALERQRIISEAREIEE
Tropheryma whipplei Endo32        QRAHILRAYLKALDQIDEVIQLIRSSKSAEEAQSNLMALLKIDETQARAILDLQLRRLAALERQRIISEAREIEE
Tropheryma whipplei Sali28        QRAHILRAYLKALDQIDEVIQLIRSSKSAEEAQSNLMALLKIDETQARAILDLQLRRLAALERQRIISEAREIEE
Tropheryma whipplei Endo27        QRAHILRAYLKALDQIDEVIQLIRSSKSAEEAQSNLMALLKIDETQARAILDLQLRRLAALERQRIISEAREIEE
Tropheryma whipplei DigMusc17     QRAHILRAYLKALDQIDEVIQLIRSSKSAEEAQSNLMALLKIDETQARAILDLQLRRLAALERQRIISEAREIEE
Tropheryma whipplei DigADP25      QRAHILRAYLKALDQIDEVIQLIRSSKSAEEAQSNLMALLKIDETQARAILDLQLRRLAALERQRIISEAREIEE
Tropheryma whipplei Dig7          QRAHILRAYLKALDQIDEVIQLIRSSKSAEEAQSNLMALLKIDETQARAILDLQLRRLAALERQRIISEAREIEE
Tropheryma whipplei Art29         QRAHILRAYLKALDQIDEVIQLIRSSKSAEEAQSNLMALLKIDETQARAILDLQLRRLAALERQRIISEAREIEE
Tropheryma whipplei Pneumo30      QRAHILRAYLKALDQIDEVIQLIRSSKSAEEAQSNLMALLKIDETQARAILDLQLRRLAALERQRIISEAREIEE

                                        460       470       480       490       500       510       520
Tropheryma whipplei Twist         LISSFRKILSDKTAQCQVIAEELREIGQKHSDARRTEIIRGFDGSVSIEDLIPVEPVVVVMTRCGYIKRTRIDQY
Tropheryma whipplei TW08/27       LISSFRKILSDKTAQCQVIAEELREIGQKHSDARRTEIIRGFDGSVSIEDLIPVEPVVVVMTRCGYIKRTRIDQY
Tropheryma whipplei slow2         LISSFRKILSDKTAQCQVIAEELREIGQKHSDARRTEIIRGFDGSVSIEDLIPVEPVVVVMTRCGYIKRTRIDQY
Tropheryma whipplei Neuro14       LISSFRKILSDKTAQCQVIAEELREIGQKHSDARRTEIIRGFDGSVSIEDLIPVEPVVVVMTRCGYIKRTRIDQY
Tropheryma whipplei Dig9          LISSFRKILSDKTAQCQVIAEELREIGQKHSDARRTEIIRGFDGSVSIEDLIPVEPVVVVMTRCGYIKRTRIDQY
Tropheryma whipplei Dig15         LISSFRKILSDKTAQCQVIAEELREIGQKHSDARRTEIIRGFDGSVSIEDLIPVEPVVVVMTRCGYIKRTRIDQY
Tropheryma whipplei Dig10         LISSFRKILSDKTAQCQVIAEELREIGQKHSDARRTEIIRGFDGSVSIEDLIPVEPVVVVMTRCGYIKRTRIDQY
Tropheryma whipplei Bcu26         LISSFRKILSDKTAQCQVIAEELREIGQKHSDARRTEIIRGFDGSVSIEDLIPVEPVVVVMTRCGYIKRTRIDQY
Tropheryma whipplei Art1          LISSFRKILSDKTAQCQVIAEELREIGQKHSDARRTEIIRGFDGSVSIEDLIPVEPVVVVMTRCGYIKRTRIDQY
Tropheryma whipplei Neuro20       LISSFRKILSDKTAQCQVIAEELREIGQKHSDARRTEIIRGFDGSVSIEDLIPVEPVVVVMTRCGYIKRTRIDQY
Tropheryma whipplei Neuro1        LISSFRKILSDKTAQCQVIAEELREIGQKHSDARRTEIIRGFDGSVSIEDLIPVEPVVVVMTRCGYIKRTRIDQY
Tropheryma whipplei shenzhen1     LISSFRKILSDKTAQCQVIAEELREIGQKHSDARRTEIIRGFDGSVSIEDLIPVEPVVVVMTRCGYIKRTRIDQY
Tropheryma whipplei shenzhen2     LISSFRKILSDKTAQCQVIAEELREIGQKHSDARRTEIIRGFDGSVSIEDLIPVEPVVVVMTRCGYIKRTRIDQY
Tropheryma whipplei Endo32        LISSFRKILSDKTAQCRVIAEELREIGQKHSDARRTEIIRGFDGSVSIEDLIPVEPVVVVMTRCGYIKRTRIDQY
Tropheryma whipplei Sali28        LISSFRKILSDKTAQCRVIAEELREIGQKHSDARRTEIIRGFDGSVSIEDLIPVEPVVVVMTRCGYIKRTRIDQY
Tropheryma whipplei Endo27        LISSFRKILSDKTAQCRVIAEELREIGQKHSDARRTEIIRGFDGSVSIEDLIPVEPVVVVMTRCGYIKRTRIDQY
Tropheryma whipplei DigMusc17     LISSFRKILSDKTAQCRVIAEELREIGQKHSDARRTEIIRGFDGSVSIEDLIPVEPVVVVMTRCGYIKRTRIDQY
Tropheryma whipplei DigADP25      LISSFRKILSDKTAQCRVIAEELREIGQKHSDARRTEIIRGFDGSVSIEDLIPVEPVVVVMTRCGYIKRTRIDQY
Tropheryma whipplei Dig7          LISSFRKILSDKTAQCRVIAEELREIGQKHSDARRTEIIRGFDGSVSIEDLIPVEPVVVVMTRCGYIKRTRIDQY
Tropheryma whipplei Art29         LISSFRKILSDKTAQCRVIAEELREIGQKHSDARRTEIIRGFDGSVSIEDLIPVEPVVVVMTRCGYIKRTRIDQY
Tropheryma whipplei Pneumo30      LISSFRKILSDKTAQCRVIAEELREIGQKHSDARRTEIIRGFDGSVSIEDLIPVEPVVVVMTRCGYIKRTRIDQY

                                 530       540       550       560       570       580       590       600
Tropheryma whipplei Twist         RSQHRGGRGVKGAQVKADDVVLQFISTTTHNWLMFFTNTGRVYRAKAYEVPEASREARGQHVANLLPLKPNEAVT
Tropheryma whipplei TW08/27       RSQHRGGRGVKGAQVKADDVVLQFISTTTHNWLMFFTNTGRVYRAKAYEVPEASREARGQHVANLLPLKPNEAVT
Tropheryma whipplei slow2         RSQHRGGRGVKGAQVKADDVVLQFISTTTHNWLMFFTNTGRVYRAKAYEVPEASREARGQHVANLLPLKPNEAVT
Tropheryma whipplei Neuro14       RSQHRGGRGVKGAQVKADDVVLQFISTTTHNWLMFFTNTGRVYRAKAYEVPEASREARGQHVANLLPLKPNEAVT
Tropheryma whipplei Dig9          RSQHRGGRGVKGAQVKADDVVLQFISTTTHNWLMFFTNTGRVYRAKAYEVPEASREARGQHVANLLPLKPNEAVT
Tropheryma whipplei Dig15         RSQHRGGRGVKGAQVKADDVVLQFISTTTHNWLMFFTNTGRVYRAKAYEVPEASREARGQHVANLLPLKPNEAVT
Tropheryma whipplei Dig10         RSQHRGGRGVKGAQVKADDVVLQFISTTTHNWLMFFTNTGRVYRAKAYEVPEASREARGQHVANLLPLKPNEAVT
Tropheryma whipplei Bcu26         RSQHRGGRGVKGAQVKADDVVLQFISTTTHNWLMFFTNTGRVYRAKAYEVPEASREARGQHVANLLPLKPNEAVT
Tropheryma whipplei Art1          RSQHRGGRGVKGAQVKADDVVLQFISTTTHNWLMFFTNTGRVYRAKAYEVPEASREARGQHVANLLPLKPNEAVT
Tropheryma whipplei Neuro20       RSQHRGGRGVKGAQVKADDVVLQFISTTTHNWLMFFTNTGRVYRAKAYEVPEASREARGQHVANLLPLKPNEAVT
Tropheryma whipplei Neuro1        RSQHRGGRGVKGAQVKADDVVLQFISTTTHNWLMFFTNTGRVYRAKAYEVPEASREARGQHVANLLPLKPNEAVT
Tropheryma whipplei shenzhen1     RSQHRGGRGVKGAQVKADDVVLQFISTTTHNWLMFFTNTGRVYRAKAYEVPEASREARGQHVANLLPLKPNEAVT
Tropheryma whipplei shenzhen2     RSQHRGGRGVKGAQVKADDVVLQFISTTTHNWLMFFTNTGRVYRAKAYEVPEASREARGQHVANLLPLKPNEAVT
Tropheryma whipplei Endo32        RSQHRGGRGVKGAQVKADDVVLQFISTTTHNWLMFFTNTGRVYRAKAYEVPEASREARGQHVANLLPLKPNEAVT
Tropheryma whipplei Sali28        RSQHRGGRGVKGAQVKADDVVLQFISTTTHNWLMFFTNTGRVYRAKAYEVPEASREARGQHVANLLPLKPNEAVT
Tropheryma whipplei Endo27        RSQHRGGRGVKGAQVKADDVVLQFISTTTHNWLMFFTNTGRVYRAKAYEVPEASREARGQHVANLLPLKPNEAVT
Tropheryma whipplei DigMusc17     RSQHRGGRGVKGAQVKADDVVLQFISTTTHNWLMFFTNTGRVYRAKAYEVPEASREARGQHVANLLPLKPNEAVT
Tropheryma whipplei DigADP25      RSQHRGGRGVKGAQVKADDVVLQFISTTTHNWLMFFTNTGRVYRAKAYEVPEASREARGQHVANLLPLKPNEAVT
Tropheryma whipplei Dig7          RSQHRGGRGVKGAQVKADDVVLQFISTTTHNWLMFFTNTGRVYRAKAYEVPEASREARGQHVANLLPLKPNEAVT
Tropheryma whipplei Art29         RSQHRGGRGVKGAQVKADDVVLQFISTTTHNWLMFFTNTGRVYRAKAYEVPEASREARGQHVANLLPLKPNEAVT
Tropheryma whipplei Pneumo30      RSQHRGGRGVKGAQVKADDVVLQFISTTTHNWLMFFTNTGRVYRAKAYEVPEASREARGQHVANLLPLKPNEAVT
```

```
                                          610       620       630       640       650       660       670
                                            .         .         .         .         .         .         .
Tropheryma whipplei Twist        RILSVSNFDSEDTSLVLATKDGYVKKTTLKEYDTSLSTGVIAIKLRPGDKLVSAVLARSDQDIILVTKNARSLRF
Tropheryma whipplei TW08/27      RILSVSNFDSEDTSLVLATKDGYVKKTTLKEYDTSLSTGVIAIKLRPGDKLVSAVLARSDQDIILVTKNARSLRF
Tropheryma whipplei slow2        RILSVSNFDSEDTSLVLATKDGYVKKTTLKEYDTSLSTGVIAIKLRPGDKLVSAVLARSDQDIILVTKNARSLRF
Tropheryma whipplei Neuro14      RILSVSNFDSEDTSLVLATKDGYVKKTTLKEYDTSLSTGVIAIKLRPGDKLVSAVLARSDQDIILVTKNARSLRF
Tropheryma whipplei Dig9         RILSVSNFDSEDTSLVLATKDGYVKKTTLKEYDTSLSTGVIAIKLRPGDKLVSAVLARSDQDIILVTKNARSLRF
Tropheryma whipplei Dig15        RILSVSNFDSEDTSLVLATKDGYVKKTTLKEYDTSLSTGVIAIKLRPGDKLVSAVLARSDQDIILVTKNARSLRF
Tropheryma whipplei Dig10        RILSVSNFDSEDTSLVLATKDGYVKKTTLKEYDTSLSTGVIAIKLRPGDKLVSAVLARSDQDIILVTKNARSLRF
Tropheryma whipplei Bcu26        RILSVSNFDSEDTSLVLATKDGYVKKTTLKEYDTSLSTGVIAIKLRPGDKLVSAVLARSDQDIILVTKNARSLRF
Tropheryma whipplei Art1         RILSVSNFDSEDTSLVLATKDGYVKKTTLKEYDTSLSTGVIAIKLRPGDKLVSAVLARSDQDIILVTKNARSLRF
Tropheryma  whipplei Neuro20     RILSVSNFDSEDTSLVLATKDGYVKKTTLKEYDTSLSTGVIAIKLRPGDKLVSAVLARSDQDIILVTKNARSLRF
Tropheryma whipplei Neuro1       RILSVSNFDSEDTSLVLATKDGYVKKTTLKEYDTSLSTGVIAIKLRPGDKLVSAVLARSDQDIILVTKNARSLRF
Tropheryma whipplei shenzhen1    RILSVSNFDSEDTSLVLATKDGYVKKTTLKEYDTSLSTGVIAIKLRPGDKLVSAVLARSDQDIILVTKNARSLRF
Tropheryma whipplei shenzhen2    RILSVSNFDSEDTSLVLATKDGYVKKTTLKEYDTSLSTGVIAIKLRPGDKLVSAVLARSDQDIILVTKNARSLRF
Tropheryma whipplei Endo32       RILSVSNFDSEDTSLVLATKDGYVKKTTLKEYDTSLSTGVIAIKLRPGDKLVSAVLARSDQDIILVTKNARSLRF
Tropheryma whipplei Sali28       RILSVSNFDSEDTSLVLATKDGYVKKTTLKEYDTSLSTGVIAIKLRPGDKLVSAVLARSDQDIILVTKNARSLRF
Tropheryma whipplei Endo27       RILSVSNFDSEDTSLVLATKDGYVKKTTLKEYDTSLSTGVIAIKLRPGDKLVSAVLARSDQDIILVTKNARSLRF
Tropheryma whipplei DigMusc17    RILSVSNFDSEDTSLVLATKDGYVKKTTLKEYDTSLSTGVIAIKLRPGDKLVSAVLARSDQDIILVTKNARSLRF
Tropheryma whipplei DigADP25     RILSVSNFDSEDTSLVLATKDGYVKKTTLKEYDTSLSTGVIAIKLRPGDKLVSAVLARSDQDIILVTKNARSLRF
Tropheryma whipplei Dig7         RILSVSNFDSEDTSLVLATKDGYVKKTTLKEYDTSLSTGVIAIKLRPGDKLVSAVLARSDQDIILVTKNARSLRF
Tropheryma whipplei Art29        RILSVSNFDSEDTSLVLATKDGYVKKTTLKEYDTSLSTGVIAIKLRPGDKLVSAVLARSDQDIILVTKNARSLRF
Tropheryma whipplei Pneumo30     RILSVSNFDSEDTSLVLATKDGYVKKTTLKEYDTSLSTGVIAIKLRPGDKLVSAVLARSDQDIILVTKNARSLRF
```

```
                                    680       690       700       710       720       730       740       750
                                      .         .         .         .         .         .         .         .
Tropheryma whipplei Twist        KSGTLRPLSRGSYGVKGISLRENDLLLSAELILSDDSYIFVVSERGYAKRSRVGDYKLQARGGIGVRVANITEQK
Tropheryma whipplei TW08/27      KSGTLRPLSRGSYGVKGISLRENDLLLSAELILSDDSYIFVVSERGYAKRSRVGDYKLQARGGIGVRVANITEQK
Tropheryma whipplei  slow2       KSGTLRPLSRGSYGVKGISLRENDLLLSAELILSDDSYIFVVSERGYAKRSRVGDYKLQARGGIGVRVANITEQK
Tropheryma whipplei Neuro14      KSGTLRPLSRGSYGVKGISLRENDLLLSAELILSDDSYIFVVSERGYAKRSRVGDYKLQARGGIGVRVANITEQK
Tropheryma whipplei Dig9         KSGTLRPLSRGSYGVKGISLRENDLLLSAELILSDDSYIFVVSERGYAKRSRVGDYKLQARGGIGVRVANITEQK
Tropheryma whipplei Dig15        KSGTLRPLSRGSYGVKGISLRENDLLLSAELILSDDSYIFVVSERGYAKRSRVGDYKLQARGGIGVRVANITEQK
Tropheryma whipplei Dig10        KSGTLRPLSRGSYGVKGISLRENDLLLSAELILSDDSYIFVVSERGYAKRSRVGDYKLQARGGIGVRVANITEQK
Tropheryma whipplei Bcu26        KSGTLRPLSRGSYGVKGISLRENDLLLSAELILSDDSYIFVVSERGYAKRSRVGDYKLQARGGIGVRVANITEQK
Tropheryma whipplei Art1         KSGTLRPLSRGSYGVKGISLRENDLLLSAELILSDDSYIFVVSERGYAKRSRVGDYKLQARGGIGVRVANITEQK
Tropheryma whipplei Neuro20      KSGTLRPLSRGSYGVKGISLRENDLLLSAELILSDDSYIFVVSERGYAKRSRVGDYKLQARGGIGVRVANITEQK
Tropheryma whipplei Neuro1       KSGTLRPLSRGSYGVKGISLRENDLLLSAELILSDDSYIFVVSERGYAKRSRVGDYKLQARGGIGVRVANITEQK
Tropheryma whipplei shenzhen1    KSGTLRPLSRGSYGVKGISLRENDLLLSAELILSDDSYIFVVSERGYAKRSRVGDYKLQTRGGIGVRVANITEQK
Tropheryma whipplei shenzhen2    KSGTLRPLSRGSYGVKGISLRENDLLLSAELILSDDSYIFVVSERGYAKRSRVGDYKLQTRGGIGVRVANITEQK
Tropheryma whipplei Endo32       KSGTLRPLSRGSYGVKGISLRENDLLLSAELILSDDSYIFVVSERGYAKRSRVGDYKLQTRGGIGVRVANITEQK
Tropheryma whipplei Sali28       KSGTLRPLSRGSYGVKGISLRENDLLLSAELILSDDSYIFVVSERGYAKRSRVGDYKLQTRGGIGVRVANITEQK
Tropheryma whipplei Endo27       KSGTLRPLSRGSYGVKGISLRENDLLLSAELILSDDSYIFVVSERGYAKRSRVGDYKLQTRGGIGVRVANITEQK
Tropheryma whipplei DigMusc17    KSGTLRPLSRGSYGVKGISLRENDLLLSAELILSDDSYIFVVSERGYAKRSRVGDYKLQTRGGIGVRVANITEQK
Tropheryma whipplei DigADP25     KSGTLRPLSRGSYGVKGISLRENDLLLSAELILSDDSYIFVVSERGYAKRSRVGDYKLQTRGGIGVRVANITEQK
Tropheryma whipplei Dig7         KSGTLRPLSRGSYGVKGISLRENDLLLSAELILSDDSYIFVVSERGYAKRSRVGDYKLQTRGGIGVRVANITEQK
Tropheryma whipplei Art29        KSGTLRPLSRGSYGVKGISLRENDLLLSAELILSDDSYIFVVSERGYAKRSRVGDYKLQTRGGIGVRVANITEQK
Tropheryma whipplei Pneumo30     KSGTLRPLSRGSYGVKGISLRENDLLLSAELILSDDSYIFVVSERGYAKRSRVGDYKLQTRGGIGVRVANITEQK
```

```
                                          760       770       780       790       800       810
                                            .         .         .         .         .         .
Tropheryma whipplei Twist        GVLADMLVVNDDDEVLVILASGKVIRSSVAEVSPTLRYTTGVVFVRMSDGDKILAMTIAEKCDDLEGQG
Tropheryma whipplei TW08/27      GVLADMLVVNDDDEVLVILASGKVIRSSVAEVSPTLRYTTGVVFVRMSDGDKILAMTIAEKCDDLEGQG
Tropheryma whipplei slow2        GVLADMLVVNDDDEVLVILASGKVIRSSVAEVSPTLRYTTGVVFVRMSDGDKILAMTIAEKCDDLEGQG
Tropheryma whipplei Neuro14      GVLADMLVVNDDDEVLVILASGKVIRSSVAEVSPTLRYTTGVVFVRMSDGDKILAMTIAEKCDDLEGQG
Tropheryma whipplei Dig9         GVLADMLVVNDDDEVLVILASGKVIRSSVAEVSPTLRYTTGVVFVRMSDGDKILAMTIAEKCDDLEGQG
Tropheryma whipplei Dig15        GVLADMLVVNDDDEVLVILASGKVIRSSVAEVSPTLRYTTGVVFVRMSDGDKILAMTIAEKCDDLEGQG
Tropheryma whipplei Dig10        GVLADMLVVNDDDEVLVILASGKVIRSSVAEVSPTLRYTTGVVFVRMSDGDKILAMTIAEKCDDLEGQG
Tropheryma whipplei Bcu26        GVLADMLVVNDDDEVLVILASGKVIRSSVAEVSPTLRYTTGVVFVRMSDGDKILAMTIAEKCDDLEGQG
Tropheryma whipplei Art1         GVLADMLVVNDDDEVLVILASGKVIRSSVAEVSPTLRYTTGVVFVRMSDGDKILAMTIAEKCDDLEGQG
Tropheryma whipplei Neuro20      GVLADMLVVNDDDEVLVILASGKVIRSSVAEVSPTLRYTTGVVFVRMSDGDKILAMTIAEKCDDLEGQG
Tropheryma whipplei Neuro1       GVLADMLVVNDDDEVLVILASGKVIRSSVAEVSPTLRYTTGVVFVRMSDGDKILAMTIAEKCDDLEGQG
Tropheryma whipplei shenzhen1    GVLADMLVVNDDDEVLVILASGKVIRSSVAEVSPTLRYTTGVVFVRMSDGDKILAMTIAEKCDDLEGQG
Tropheryma whipplei shenzhen2    GVLADMLVVNDDDEVLVILASGKVIRSSVAEVSPTLRYTTGVVFVRMSDGDKILAMTIAEKCDDLEGQG
Tropheryma whipplei Endo32       GVLADMLVVNDDDEVLVILASGKVIRSSVAEVSPTLRYTTGVVFVRMSDGDKILAMTIAEKCDDLEGQG
Tropheryma whipplei Sali28       GVLADMLVVNDDDEVLVILASGKVIRSSVAEVSPTLRYTTGVVFVRMSDGDKILAMTIAEKCDDLEGQG
Tropheryma whipplei Endo27       GVLADMLVVNDDDEVLVILASGKVIRSSVAEVSPTLRYTTGVVFVRMSDGDKILAMTIAEKCDDLEGQG
Tropheryma whipplei DigMusc17    GVLADMLVVNDDDEVLVILASGKVIRSSVAEVSPTLRYTTGVVFVRMSDGDKILAMTIAEKCDDLEGQG
Tropheryma whipplei DigADP25     GVLADMLVVNDDDEVLVILASGKVIRSSVAEVSPTLRYTTGVVFVRMSDGDKILAMTIAEKCDDLEGQG
Tropheryma whipplei Dig7         GVLADMLVVNDDDEVLVILASGKVIRSSVAEVSPTLRYTTGVVFVRMSDGDKILAMTIAEKCDDLEGQG
Tropheryma whipplei Art29        GVLADMLVVNDDDEVLVILASGKVIRSSVAEVSPTLRYTTGVVFVRMSDGDKILAMTIAEKCDDLEGQG
Tropheryma whipplei Pneumo30     GVLADMLVVNDDDEVLVILASGKVIRSSVAEVSPTLRYTTGVVFVRMSDGDKILAMTIAEKCDDLEGQG
```
