# Supplementary material for: Genomic characterization of two metagenome-assembled genomes of Tropheryma whipplei from China
Source: Front Cell Infect Microbiol. 2022 Sep 16;12:947486. doi: 10.3389/fcimb.2022.947486 (PMC9523146; doi:10.3389/fcimb.2022.947486)
Supplement: Supplementary file 2 [file DataSheet_2.pdf]

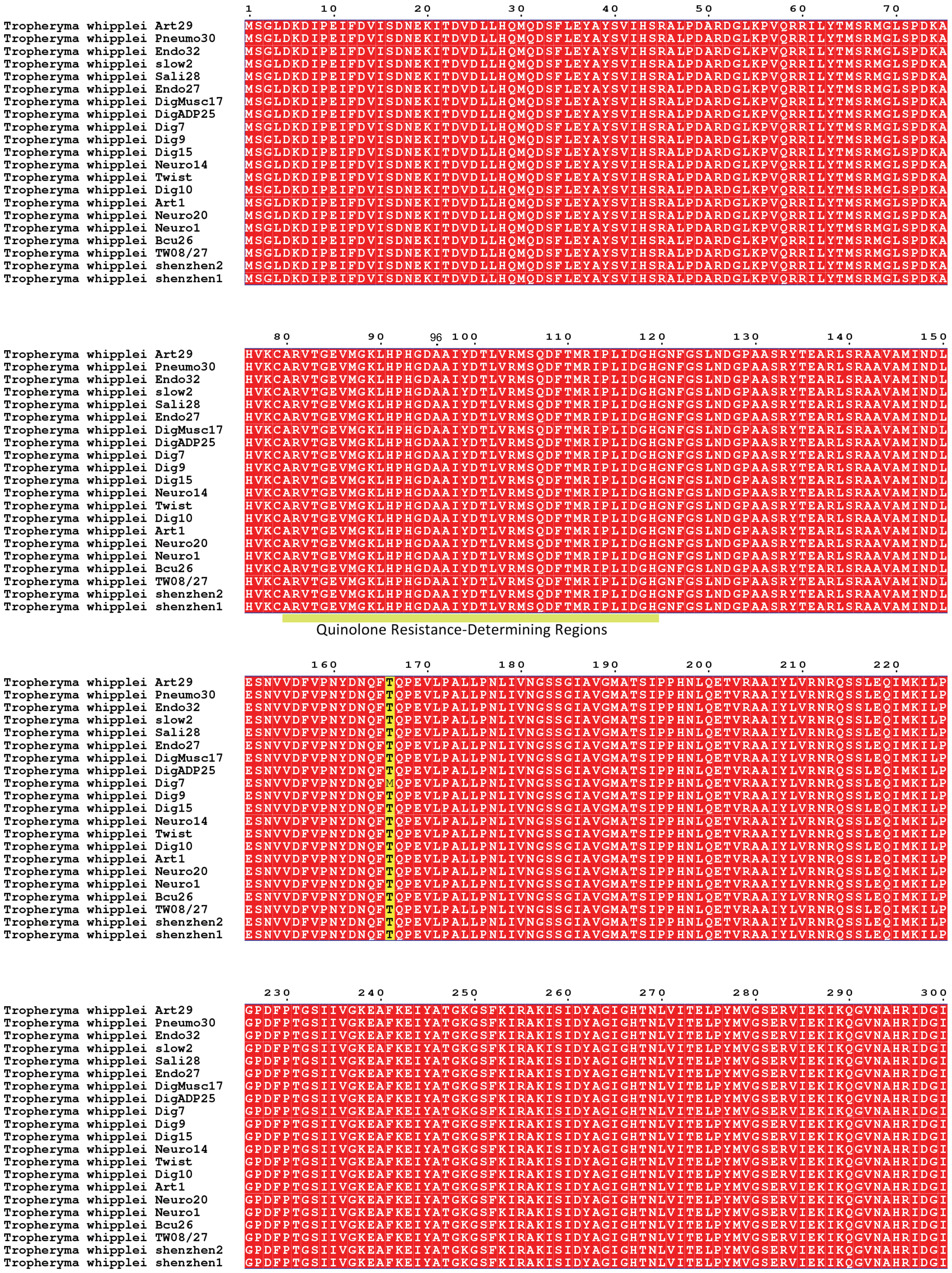
1 10 20 30 40 50 60 70
Tropheryma whipplei Art29 MSGLDKDIPEIFDVISDNEKITDVDLLHQMQDSFLEYAYSVIHSRALPDARDGLKPVQRRILYTMSRMGLSPDKA
Tropheryma whipplei Pneumo30 MSGLDKDIPEIFDVISDNEKITDVDLLHQMQDSFLEYAYSVIHSRALPDARDGLKPVQRRILYTMSRMGLSPDKA
Tropheryma whipplei Endo32 MSGLDKDIPEIFDVISDNEKITDVDLLHQMQDSFLEYAYSVIHSRALPDARDGLKPVQRRILYTMSRMGLSPDKA
Tropheryma whipplei slow2 MSGLDKDIPEIFDVISDNEKITDVDLLHQMQDSFLEYAYSVIHSRALPDARDGLKPVQRRILYTMSRMGLSPDKA
Tropheryma whipplei Sali28 MSGLDKDIPEIFDVISDNEKITDVDLLHQMQDSFLEYAYSVIHSRALPDARDGLKPVQRRILYTMSRMGLSPDKA
Tropheryma whipplei Endo27 MSGLDKDIPEIFDVISDNEKITDVDLLHQMQDSFLEYAYSVIHSRALPDARDGLKPVQRRILYTMSRMGLSPDKA
Tropheryma whipplei DigMusc17 MSGLDKDIPEIFDVISDNEKITDVDLLHQMQDSFLEYAYSVIHSRALPDARDGLKPVQRRILYTMSRMGLSPDKA
Tropheryma whipplei DigADP25 MSGLDKDIPEIFDVISDNEKITDVDLLHQMQDSFLEYAYSVIHSRALPDARDGLKPVQRRILYTMSRMGLSPDKA
Tropheryma whipplei Dig7 MSGLDKDIPEIFDVISDNEKITDVDLLHQMQDSFLEYAYSVIHSRALPDARDGLKPVQRRILYTMSRMGLSPDKA
Tropheryma whipplei Dig9 MSGLDKDIPEIFDVISDNEKITDVDLLHQMQDSFLEYAYSVIHSRALPDARDGLKPVQRRILYTMSRMGLSPDKA
Tropheryma whipplei Dig15 MSGLDKDIPEIFDVISDNEKITDVDLLHQMQDSFLEYAYSVIHSRALPDARDGLKPVQRRILYTMSRMGLSPDKA
Tropheryma whipplei Neuro14 MSGLDKDIPEIFDVISDNEKITDVDLLHQMQDSFLEYAYSVIHSRALPDARDGLKPVQRRILYTMSRMGLSPDKA
Tropheryma whipplei Twist MSGLDKDIPEIFDVISDNEKITDVDLLHQMQDSFLEYAYSVIHSRALPDARDGLKPVQRRILYTMSRMGLSPDKA
Tropheryma whipplei Dig10 MSGLDKDIPEIFDVISDNEKITDVDLLHQMQDSFLEYAYSVIHSRALPDARDGLKPVQRRILYTMSRMGLSPDKA
Tropheryma whipplei Art1 MSGLDKDIPEIFDVISDNEKITDVDLLHQMQDSFLEYAYSVIHSRALPDARDGLKPVQRRILYTMSRMGLSPDKA
Tropheryma whipplei Neuro20 MSGLDKDIPEIFDVISDNEKITDVDLLHQMQDSFLEYAYSVIHSRALPDARDGLKPVQRRILYTMSRMGLSPDKA
Tropheryma whipplei Neuro1 MSGLDKDIPEIFDVISDNEKITDVDLLHQMQDSFLEYAYSVIHSRALPDARDGLKPVQRRILYTMSRMGLSPDKA
Tropheryma whipplei Bcu26 MSGLDKDIPEIFDVISDNEKITDVDLLHQMQDSFLEYAYSVIHSRALPDARDGLKPVQRRILYTMSRMGLSPDKA
Tropheryma whipplei TW08/27 MSGLDKDIPEIFDVISDNEKITDVDLLHQMQDSFLEYAYSVIHSRALPDARDGLKPVQRRILYTMSRMGLSPDKA
Tropheryma whipplei shenzhen2 MSGLDKDIPEIFDVISDNEKITDVDLLHQMQDSFLEYAYSVIHSRALPDARDGLKPVQRRILYTMSRMGLSPDKA
Tropheryma whipplei shenzhen1 MSGLDKDIPEIFDVISDNEKITDVDLLHQMQDSFLEYAYSVIHSRALPDARDGLKPVQRRILYTMSRMGLSPDKA
80 90 96 100 110 120 130 140 150
Tropheryma whipplei Art29 HVKCARVTGEVMGKLHPHGDAAIYDTLVRMSQDFTMRIPLIDGHGNFGSLNDGPAASRYTEARLSRAAVAMINDL
Tropheryma whipplei Pneumo30 HVKCARVTGEVMGKLHPHGDAAIYDTLVRMSQDFTMRIPLIDGHGNFGSLNDGPAASRYTEARLSRAAVAMINDL
Tropheryma whipplei Endo32 HVKCARVTGEVMGKLHPHGDAAIYDTLVRMSQDFTMRIPLIDGHGNFGSLNDGPAASRYTEARLSRAAVAMINDL
Tropheryma whipplei slow2 HVKCARVTGEVMGKLHPHGDAAIYDTLVRMSQDFTMRIPLIDGHGNFGSLNDGPAASRYTEARLSRAAVAMINDL
Tropheryma whipplei Sali28 HVKCARVTGEVMGKLHPHGDAAIYDTLVRMSQDFTMRIPLIDGHGNFGSLNDGPAASRYTEARLSRAAVAMINDL
Tropheryma whipplei Endo27 HVKCARVTGEVMGKLHPHGDAAIYDTLVRMSQDFTMRIPLIDGHGNFGSLNDGPAASRYTEARLSRAAVAMINDL
Tropheryma whipplei DigMusc17 HVKCARVTGEVMGKLHPHGDAAIYDTLVRMSQDFTMRIPLIDGHGNFGSLNDGPAASRYTEARLSRAAVAMINDL
Tropheryma whipplei DigADP25 HVKCARVTGEVMGKLHPHGDAAIYDTLVRMSQDFTMRIPLIDGHGNFGSLNDGPAASRYTEARLSRAAVAMINDL
Tropheryma whipplei Dig7 HVKCARVTGEVMGKLHPHGDAAIYDTLVRMSQDFTMRIPLIDGHGNFGSLNDGPAASRYTEARLSRAAVAMINDL
Tropheryma whipplei Dig9 HVKCARVTGEVMGKLHPHGDAAIYDTLVRMSQDFTMRIPLIDGHGNFGSLNDGPAASRYTEARLSRAAVAMINDL
Tropheryma whipplei Dig15 HVKCARVTGEVMGKLHPHGDAAIYDTLVRMSQDFTMRIPLIDGHGNFGSLNDGPAASRYTEARLSRAAVAMINDL
Tropheryma whipplei Neuro14 HVKCARVTGEVMGKLHPHGDAAIYDTLVRMSQDFTMRIPLIDGHGNFGSLNDGPAASRYTEARLSRAAVAMINDL
Tropheryma whipplei Twist HVKCARVTGEVMGKLHPHGDAAIYDTLVRMSQDFTMRIPLIDGHGNFGSLNDGPAASRYTEARLSRAAVAMINDL
Tropheryma whipplei Dig10 HVKCARVTGEVMGKLHPHGDAAIYDTLVRMSQDFTMRIPLIDGHGNFGSLNDGPAASRYTEARLSRAAVAMINDL
Tropheryma whipplei Art1 HVKCARVTGEVMGKLHPHGDAAIYDTLVRMSQDFTMRIPLIDGHGNFGSLNDGPAASRYTEARLSRAAVAMINDL
Tropheryma whipplei Neuro20 HVKCARVTGEVMGKLHPHGDAAIYDTLVRMSQDFTMRIPLIDGHGNFGSLNDGPAASRYTEARLSRAAVAMINDL
Tropheryma whipplei Neuro1 HVKCARVTGEVMGKLHPHGDAAIYDTLVRMSQDFTMRIPLIDGHGNFGSLNDGPAASRYTEARLSRAAVAMINDL
Tropheryma whipplei Bcu26 HVKCARVTGEVMGKLHPHGDAAIYDTLVRMSQDFTMRIPLIDGHGNFGSLNDGPAASRYTEARLSRAAVAMINDL
Tropheryma whipplei TW08/27 HVKCARVTGEVMGKLHPHGDAAIYDTLVRMSQDFTMRIPLIDGHGNFGSLNDGPAASRYTEARLSRAAVAMINDL
Tropheryma whipplei shenzhen2 HVKCARVTGEVMGKLHPHGDAAIYDTLVRMSQDFTMRIPLIDGHGNFGSLNDGPAASRYTEARLSRAAVAMINDL
Tropheryma whipplei shenzhen1 HVKCARVTGEVMGKLHPHGDAAIYDTLVRMSQDFTMRIPLIDGHGNFGSLNDGPAASRYTEARLSRAAVAMINDL
Quinolone Resistance-Determining Regions
160 170 180 190 200 210 220
Tropheryma whipplei Art29 ESNVVDFVPNYDNQFTQPEVLPALLPNLIVNGSSGIAVGMATSIPPHNLQETVRAAIYLVRNRQSSLEQIMKILP
Tropheryma whipplei Pneumo30 ESNVVDFVPNYDNQFTQPEVLPALLPNLIVNGSSGIAVGMATSIPPHNLQETVRAAIYLVRNRQSSLEQIMKILP
Tropheryma whipplei Endo32 ESNVVDFVPNYDNQFTQPEVLPALLPNLIVNGSSGIAVGMATSIPPHNLQETVRAAIYLVRNRQSSLEQIMKILP
Tropheryma whipplei slow2 ESNVVDFVPNYDNQFTQPEVLPALLPNLIVNGSSGIAVGMATSIPPHNLQETVRAAIYLVRNRQSSLEQIMKILP
Tropheryma whipplei Sali28 ESNVVDFVPNYDNQFTQPEVLPALLPNLIVNGSSGIAVGMATSIPPHNLQETVRAAIYLVRNRQSSLEQIMKILP
Tropheryma whipplei Endo27 ESNVVDFVPNYDNQFTQPEVLPALLPNLIVNGSSGIAVGMATSIPPHNLQETVRAAIYLVRNRQSSLEQIMKILP
Tropheryma whipplei DigMusc17 ESNVVDFVPNYDNQFTQPEVLPALLPNLIVNGSSGIAVGMATSIPPHNLQETVRAAIYLVRNRQSSLEQIMKILP
Tropheryma whipplei DigADP25 ESNVVDFVPNYDNQFTQPEVLPALLPNLIVNGSSGIAVGMATSIPPHNLQETVRAAIYLVRNRQSSLEQIMKILP
Tropheryma whipplei Dig7 ESNVVDFVPNYDNQFMQPEVLPALLPNLIVNGSSGIAVGMATSIPPHNLQETVRAAIYLVRNRQSSLEQIMKILP
Tropheryma whipplei Dig9 ESNVVDFVPNYDNQFTQPEVLPALLPNLIVNGSSGIAVGMATSIPPHNLQETVRAAIYLVRNRQSSLEQIMKILP
Tropheryma whipplei Dig15 ESNVVDFVPNYDNQFTQPEVLPALLPNLIVNGSSGIAVGMATSIPPHNLQETVRAAIYLVRNRQSSLEQIMKILP
Tropheryma whipplei Neuro14 ESNVVDFVPNYDNQFTQPEVLPALLPNLIVNGSSGIAVGMATSIPPHNLQETVRAAIYLVRNRQSSLEQIMKILP
Tropheryma whipplei Twist ESNVVDFVPNYDNQFTQPEVLPALLPNLIVNGSSGIAVGMATSIPPHNLQETVRAAIYLVRNRQSSLEQIMKILP
Tropheryma whipplei Dig10 ESNVVDFVPNYDNQFTQPEVLPALLPNLIVNGSSGIAVGMATSIPPHNLQETVRAAIYLVRNRQSSLEQIMKILP
Tropheryma whipplei Art1 ESNVVDFVPNYDNQFTQPEVLPALLPNLIVNGSSGIAVGMATSIPPHNLQETVRAAIYLVRNRQSSLEQIMKILP
Tropheryma whipplei Neuro20 ESNVVDFVPNYDNQFTQPEVLPALLPNLIVNGSSGIAVGMATSIPPHNLQETVRAAIYLVRNRQSSLEQIMKILP
Tropheryma whipplei Neuro1 ESNVVDFVPNYDNQFTQPEVLPALLPNLIVNGSSGIAVGMATSIPPHNLQETVRAAIYLVRNRQSSLEQIMKILP
Tropheryma whipplei Bcu26 ESNVVDFVPNYDNQFTQPEVLPALLPNLIVNGSSGIAVGMATSIPPHNLQETVRAAIYLVRNRQSSLEQIMKILP
Tropheryma whipplei TW08/27 ESNVVDFVPNYDNQFTQPEVLPALLPNLIVNGSSGIAVGMATSIPPHNLQETVRAAIYLVRNRQSSLEQIMKILP
Tropheryma whipplei shenzhen2 ESNVVDFVPNYDNQFTQPEVLPALLPNLIVNGSSGIAVGMATSIPPHNLQETVRAAIYLVRNRQSSLEQIMKILP
Tropheryma whipplei shenzhen1 ESNVVDFVPNYDNQFTQPEVLPALLPNLIVNGSSGIAVGMATSIPPHNLQETVRAAIYLVRNRQSSLEQIMKILP
230 240 250 260 270 280 290 300
Tropheryma whipplei Art29 GPDFPTGSIIVGKEAFKEIYATGKGSFKIRAKISIDYAGIGHTNLVITELPYMVGSERVIEKIKQGVNAHRIDGI
Tropheryma whipplei Pneumo30 GPDFPTGSIIVGKEAFKEIYATGKGSFKIRAKISIDYAGIGHTNLVITELPYMVGSERVIEKIKQGVNAHRIDGI
Tropheryma whipplei Endo32 GPDFPTGSIIVGKEAFKEIYATGKGSFKIRAKISIDYAGIGHTNLVITELPYMVGSERVIEKIKQGVNAHRIDGI
Tropheryma whipplei slow2 GPDFPTGSIIVGKEAFKEIYATGKGSFKIRAKISIDYAGIGHTNLVITELPYMVGSERVIEKIKQGVNAHRIDGI
Tropheryma whipplei Sali28 GPDFPTGSIIVGKEAFKEIYATGKGSFKIRAKISIDYAGIGHTNLVITELPYMVGSERVIEKIKQGVNAHRIDGI
Tropheryma whipplei Endo27 GPDFPTGSIIVGKEAFKEIYATGKGSFKIRAKISIDYAGIGHTNLVITELPYMVGSERVIEKIKQGVNAHRIDGI
Tropheryma whipplei DigMusc17 GPDFPTGSIIVGKEAFKEIYATGKGSFKIRAKISIDYAGIGHTNLVITELPYMVGSERVIEKIKQGVNAHRIDGI
Tropheryma whipplei DigADP25 GPDFPTGSIIVGKEAFKEIYATGKGSFKIRAKISIDYAGIGHTNLVITELPYMVGSERVIEKIKQGVNAHRIDGI
Tropheryma whipplei Dig7 GPDFPTGSIIVGKEAFKEIYATGKGSFKIRAKISIDYAGIGHTNLVITELPYMVGSERVIEKIKQGVNAHRIDGI
Tropheryma whipplei Dig9 GPDFPTGSIIVGKEAFKEIYATGKGSFKIRAKISIDYAGIGHTNLVITELPYMVGSERVIEKIKQGVNAHRIDGI
Tropheryma whipplei Dig15 GPDFPTGSIIVGKEAFKEIYATGKGSFKIRAKISIDYAGIGHTNLVITELPYMVGSERVIEKIKQGVNAHRIDGI
Tropheryma whipplei Neuro14 GPDFPTGSIIVGKEAFKEIYATGKGSFKIRAKISIDYAGIGHTNLVITELPYMVGSERVIEKIKQGVNAHRIDGI
Tropheryma whipplei Twist GPDFPTGSIIVGKEAFKEIYATGKGSFKIRAKISIDYAGIGHTNLVITELPYMVGSERVIEKIKQGVNAHRIDGI
Tropheryma whipplei Dig10 GPDFPTGSIIVGKEAFKEIYATGKGSFKIRAKISIDYAGIGHTNLVITELPYMVGSERVIEKIKQGVNAHRIDGI
Tropheryma whipplei Art1 GPDFPTGSIIVGKEAFKEIYATGKGSFKIRAKISIDYAGIGHTNLVITELPYMVGSERVIEKIKQGVNAHRIDGI
Tropheryma whipplei Neuro20 GPDFPTGSIIVGKEAFKEIYATGKGSFKIRAKISIDYAGIGHTNLVITELPYMVGSERVIEKIKQGVNAHRIDGI
Tropheryma whipplei Neuro1 GPDFPTGSIIVGKEAFKEIYATGKGSFKIRAKISIDYAGIGHTNLVITELPYMVGSERVIEKIKQGVNAHRIDGI
Tropheryma whipplei Bcu26 GPDFPTGSIIVGKEAFKEIYATGKGSFKIRAKISIDYAGIGHTNLVITELPYMVGSERVIEKIKQGVNAHRIDGI
Tropheryma whipplei TW08/27 GPDFPTGSIIVGKEAFKEIYATGKGSFKIRAKISIDYAGIGHTNLVITELPYMVGSERVIEKIKQGVNAHRIDGI
Tropheryma whipplei shenzhen2 GPDFPTGSIIVGKEAFKEIYATGKGSFKIRAKISIDYAGIGHTNLVITELPYMVGSERVIEKIKQGVNAHRIDGI
Tropheryma whipplei shenzhen1 GPDFPTGSIIVGKEAFKEIYATGKGSFKIRAKISIDYAGIGHTNLVITELPYMVGSERVIEKIKQGVNAHRIDGI

```
                                     310       320       330       340       350       360       370
Tropheryma whipplei Art29       LDVTDLSDRKNGLKLVIGIKPNFSPNEVMDNLYRHTPLEESFSVNSVALIDGKPKTLGMLDMLKVFIDHRVCVIT
Tropheryma whipplei Pneumo30    LDVTDLSDRKNGLKLVIGIKPNFSPNEVMDNLYRHTPLEESFSVNSVALIDGKPKTLGMLDMLKVFIDHRVCVIT
Tropheryma whipplei Endo32      LDVTDLSDRKNGLKLVIGIKPNFSPNEVMDNLYRHTPLEESFSVNSVALIDGKPKTLGMLDMLKVFIDHRVCVIT
Tropheryma whipplei slow2       LDVTDLSDRKNGLKLVIGIKPNFSPNEVMDNLYRHTPLEESFSVNSVALIDGKPKTLGMLDMLKVFIDHRVCVIT
Tropheryma whipplei Sali28      LDVTDLSDRKNGLKLVIGIKPNFSPNEVMDNLYRHTPLEESFSVNSVALIDGKPKTLGMLDMLKVFIDHRVCVIT
Tropheryma whipplei Endo27      LDVTDLSDRKNGLKLVIGIKPNFSPNEVMDNLYRHTPLEESFSVNSVALIDGKPKTLGMLDMLKVFIDHRVCVIT
Tropheryma whipplei DigMusc17   LDVTDLSDRKNGLKLVIGIKPNFSPNEVMDNLYRHTPLEESFSVNSVALIDGKPKTLGMLDMLKVFIDHRVCVIT
Tropheryma whipplei DigADP25    LDVTDLSDRKNGLKLVIGIKPNFSPNEVMDNLYRHTPLEESFSVNSVALIDGKPKTLGMLDMLKVFIDHRVCVIT
Tropheryma whipplei Dig7        LDVTDLSDRKNGLKLVIGIKPNFSPNEVMDNLYRHTPLEESFSVNSVALIDGKPKTLGMLDMLKVFIDHRVCVIT
Tropheryma whipplei Dig9        LDVTDLSDRKNGLKLVIGIKPNFSPNEVMDNLYRHTPLEESFSVNSVALIDGKPKTLGMLDMLKVFIDHRVCVIT
Tropheryma whipplei Dig15       LDVTDLSDRKNGLKLVIGIKPNFSPNEVMDNLYRHTPLEESFSVNSVALIDGKPKTLGMLDMLKVFIDHRVCVIT
Tropheryma whipplei Neuro14     LDVTDLSDRKNGLKLVIGIKPNFSPNEVMDNLYRHTPLEESFSVNSVALIDGKPKTLGMLDMLKVFIDHRVCVIT
Tropheryma whipplei Twist       LDVTDLSDRKNGLKLVIGIKPNFSPNEVMDNLYRHTPLEESFSVNSVALIDGKPKTLGMLDMLKVFIDHRVCVIT
Tropheryma whipplei Dig10       LDVTDLSDRKNGLKLVIGIKPNFSPNEVMDNLYRHTPLEESFSVNSVALIDGKPKTLGMLDMLKVFIDHRVCVIT
Tropheryma whipplei Art1        LDVTDLSDRKNGLKLVIGIKPNFSPNEVMDNLYRHTPLEESFSVNSVALIDGKPKTLGMLDMLKVFIDHRVCVIT
Tropheryma whipplei Neuro20     LDVTDLSDRKNGLKLVIGIKPNFSPNEVMDNLYRHTPLEESFSVNSVALIDGKPKTLGMLDMLKVFIDHRVCVIT
Tropheryma whipplei Neuro1      LDVTDLSDRKNGLKLVIGIKPNFSPNEVMDNLYRHTPLEESFSVNSVALIDGKPKTLGMLDMLKVFIDHRVCVIT
Tropheryma whipplei Bcu26       LDVTDLSDRKNGLKLVIGIKPNFSPNEVMDNLYRHTPLEESFSVNSVALIDGKPKTLGMLDMLKVFIDHRVCVIT
Tropheryma whipplei TW08/27     LDVTDLSDRKNGLKLVIGIKPNFSPNEVMDNLYRHTPLEESFSVNSVALIDGKPKTLGMLDMLKVFIDHRVCVIT
Tropheryma whipplei shenzhen2   LDVTDLSDRKNGLKLVIGIKPNFSPNEVMDNLYRHTPLEESFSVNSVALIDGKPKTLGMLDMLKVFIDHRVCVIT
Tropheryma whipplei shenzhen1   LDVTDLSDRKNGLKLVIGIKPNFSPNEVMDNLYRHTPLEESFSVNSVALIDGKPKTLGMLDMLKVFIDHRVCVIT
```

```
                                380       390       400       410       420       430       440       450
Tropheryma whipplei Art29       RRSKFYLATHKKRLHMIDGLLLALVDIDKTISIIRNSEGIKEARKNLMKCLNVTRDQAEYILDIRLNMLTKLSTI
Tropheryma whipplei Pneumo30    RRSKFYLATHKKRLHMIDGLLLALVDIDKTISIIRNSEGIKEARKNLMKCLNVTRDQAEYILDIRLNMLTKLSTI
Tropheryma whipplei Endo32      RRSKFYLATHKKRLHMIDGLLLALVDIDKTISIIRNSEGIKEARKNLMKCLNVTRDQAEYILDIRLNMLTKLSTI
Tropheryma whipplei slow2       RRSKFYLATHKKRLHMIDGLLLALVDIDKTISIIRNSEGIKEARKNLMKCLNVTRDQAEYILDIRLNMLTKLSTI
Tropheryma whipplei Sali28      RRSKFYLATHKKRLHMIDGLLLALVDIDKTISIIRNSEGIKEARKNLMKCLNVTRDQAEYILDIRLNMLTKLSTI
Tropheryma whipplei Endo27      RRSKFYLATHKKRLHMIDGLLLALVDIDKTISIIRNSEGIKEARKNLMKCLNVTRDQAEYILDIRLNMLTKLSTI
Tropheryma whipplei DigMusc17   RRSKFYLATHKKRLHMIDGLLLALVDIDKTISIIRNSEGIKEARKNLMKCLNVTRDQAEYILDIRLNMLTKLSTI
Tropheryma whipplei DigADP25    RRSKFYLATHKKRLHMIDGLLLALVDIDKTISIIRNSEGIKEARKNLMKCLNVTRDQAEYILDIRLNMLTKLSTI
Tropheryma whipplei Dig7        RRSKFYLATHKKRLHMIDGLLLALVDIDKTISIIRNSEGIKEARKNLMKCLNVTRDQAEYILDIRLNMLTKLSTI
Tropheryma whipplei Dig9        RRSKFYLATHKKRLHMIDGLLLALVDIDKTISIIRNSEGIKEARKNLMKCLNVTRDQAEYILDIRLNMLTKLSTI
Tropheryma whipplei Dig15       RRSKFYLATHKKRLHMIDGLLLALVDIDKTISIIRNSEGIKEARKNLMKCLNVTRDQAEYILDIRLNMLTKLSTI
Tropheryma whipplei Neuro14     RRSKFYLATHKKRLHMIDGLLLALVDIDKTISIIRNSEGIKEARKNLMKCLNVTRDQAEYILDIRLNMLTKLSTI
Tropheryma whipplei Twist       RRSKFYLATHKKRLHMIDGLLLALVDIDKTISIIRNSEGIKEARKNLMKCLNVTRDQAEYILDIRLNMLTKLSTI
Tropheryma whipplei Dig10       RRSKFYLATHKKRLHMIDGLLLALVDIDKTISIIRNSEGIKEARKNLMKCLNVTRDQAEYILDIRLNMLTKLSTI
Tropheryma whipplei Art1        RRSKFYLATHKKRLHMIDGLLLALVDIDKTISIIRNSEGIKEARKNLMKCLNVTRDQAEYILDIRLNMLTKLSTI
Tropheryma whipplei Neuro20     RRSKFYLATHKKRLHMIDGLLLALVDIDKTISIIRNSEGIKEARKNLMKCLNVTRDQAEYILDIRLNMLTKLSTI
Tropheryma whipplei Neuro1      RRSKFYLATHKKRLHMIDGLLLALVDIDKTISIIRNSEGIKEARKNLMKCLNVTRDQAEYILDIRLNMLTKLSTI
Tropheryma whipplei Bcu26       RRSKFYLATHKKRLHMIDGLLLALVDIDKTISIIRNSEGIKEARKNLMKCLNVTRDQAEYILDIRLNMLTKLSTI
Tropheryma whipplei TW08/27     RRSKFYLATHKKRLHMIDGLLLALVDIDKTISIIRNSEGIKEARKNLMKCLNVTRDQAEYILDIRLNMLTKLSTI
Tropheryma whipplei shenzhen2   RRSKFYLATHKKRLHMIDGLLLALVDIDKTISIIRNSEGIKEARKNLMKCLNVTRDQAEYILDIRLNMLTKLSTI
Tropheryma whipplei shenzhen1   RRSKFYLATHKKRLHMIDGLLLALVDIDKTISIIRNSEGIKEARKNLMKCLNVTRDQAEYILDIRLNMLTKLSTI
```

```
                                     460       470       480       490       500       510       520
Tropheryma whipplei Art29       KLQSEQEELRKTTQDLEEILSDPNKIDAILIDELKKVAQRLPSPRRTILESSVKSKKQKTDYPRQVVLTTNGFIF
Tropheryma whipplei Pneumo30    KLQSEQEELRKTTQDLEEILSDPNKIDAILIDELKKVAQRLPSPRRTILESSVKSKKQKTDYPRQVVLTTNGFIF
Tropheryma whipplei Endo32      KLQSEQEELRKTTQDLEEILSDPNKIDAILIDELKKVAQRLPSPRRTILESSVKSKKQKTDYPRQVVLTTNGFIF
Tropheryma whipplei slow2       KLQSEQEELRKTTQDLEEILSDPNKIDAILIDELKKVAQRLPSPRRTILESSVKSKKQKTDYPRQVVLTTNGFIF
Tropheryma whipplei Sali28      KLQSEQEELRKTTQDLEEILSDPNKIDAILIDELKKVAQRLPSPRRTILESSVKSKKQKTDYPRQVVLTTNGFIF
Tropheryma whipplei Endo27      KLQSEQEELRKTTQDLEEILSDPNKIDAILIDELKKVAQRLPSPRRTILESSVKSKKQKTDYPRQVVLTTNGFIF
Tropheryma whipplei DigMusc17   KLQSEQEELRKTTQDLEEILSDPNKIDAILIDELKKVAQRLPSPRRTILESSVKSKKQKTDYPRQVVLTTNGFIF
Tropheryma whipplei DigADP25    KLQSEQEELRKTTQDLEEILSDPNKIDAILIDELKKVAQRLPSPRRTILESSVKSKKQKTDYPRQVVLTTNGFIF
Tropheryma whipplei Dig7        KLQSEQEELRKTTQDLEEILSDPNKIDAILIDELKKVAQRLPSPRRTILESSVKSKKQKTDYPRQVVLTTNGFIF
Tropheryma whipplei Dig9        KLQSEQEELRKTTQDLEEILSDPNKIDAILIDELKKVAQRLPSPRRTILESSVKSKKQKTDYPRQVVLTTNGFIF
Tropheryma whipplei Dig15       KLQSEQEELRKTTQDLEEILSDPNKIDAILIDELKKVAQRLPSPRRTILESSVKSKKQKTDYPRQVVLTTNGFIF
Tropheryma whipplei Neuro14     KLQSEQEELRKTTQDLEEILSDPNKIDAILIDELKKVAQRLPSPRRTILESSVKSKKQKTDYPRQVVLTTNGFIF
Tropheryma whipplei Twist       KLQSEQEELRKTTQDLEEILSDPNKIDAILIDELKKVAQRLPSPRRTILESSVKSKKQKTDYPRQVVLTTNGFIF
Tropheryma whipplei Dig10       KLQSEQEELRKTTQDLEEILSDPNKIDAILIDELKKVAQRLPSPRRTILESSVKSKKQKTDYPRQVVLTTNGFIF
Tropheryma whipplei Art1        KLQSEQEELRKTTQDLEEILSDPNKIDAILIDELKKVAQRLPSPRRTILESSVKSKKQKTDYPRQVVLTTNGFIF
Tropheryma whipplei Neuro20     KLQSEQEELRKTTQDLEEILSDPNKIDAILIDELKKVAQRLPSPRRTILESSVKSKKQKTDYPRQVVLTTNGFIF
Tropheryma whipplei Neuro1      KLQSEQEELRKTTQDLEEILSDPNKIDAILIDELKKVAQRLPSPRRTILESSVKSKKQKTDYPRQVVLTTNGFIF
Tropheryma whipplei Bcu26       KLQSEQEELRKTTQDLEEILSDPNKIDAILIDELKKVAQRLPSPRRTILESSVKSKKQKTDYPRQVVLTTNGFIF
Tropheryma whipplei TW08/27     KLQSEQEELRKTTQDLEEILSDPNKIDAILIDELKKVAQRLPSPRRTILESSVKSKKQKTDYPRQVVLTTNGFIF
Tropheryma whipplei shenzhen2   KLQSEQEELRKTTQDLEEILSDPNKIDAILIDELKKVAQRLPSPRRTILESSVKSKKQKTDYPRQVVLTTNGFIF
Tropheryma whipplei shenzhen1   KLQSEQEELRKTTQDLEEILSDPNKIDAILIDELKKVAQRLPSPRRTILESSVKSKKQKTDYPRQVVLTTNGFIF
```

```
                                530       540       550       560       570       580       590       600
Tropheryma whipplei Art29       TIEVNNDLISELSQPETFDMAFNTRSEPLRVVKSSIVCGVRSRIGALTSAGKVIPFTPIGLPVLDSGFVTAKSFL
Tropheryma whipplei Pneumo30    TIEVNNDLISELSQPETFDMAFNTRSEPLRVVKSSIVCGVRSRIGALTSAGKVIPFTPIGLPVLDSGFVTAKSFL
Tropheryma whipplei Endo32      TIEVNNDLISELSQPETFDMAFNTRSEPLRVVKSSIVCGVRSRIGALTSAGKVIPFTPIGLPVLDSGFVTAKSFL
Tropheryma whipplei slow2       TIEVNNDLISELSQPETFDMAFNTRSEPLRVVKSSIVCGVRSRIGALTSAGKVIPFTPIGLPVLDSGFVTAKSFL
Tropheryma whipplei Sali28      TIEVNNDLISELSQPETFDMAFNTRSEPLRVVKSSIVCGVRSRIGALTSAGKVIPFTPIGLPVLDSGFVTAKSFL
Tropheryma whipplei Endo27      TIEVNNDLISELSQPETFDMAFNTRSEPLRVVKSSIVCGVRSRIGALTSAGKVIPFTPIGLPVLDSGFVTAKSFL
Tropheryma whipplei DigMusc17   TIEVNNDLISELSQPETFDMAFNTRSEPLRVVKSSIVCGVRSRIGALTSAGKVIPFTPIGLPVLDSGFVTAKSFL
Tropheryma whipplei DigADP25    TIEVNNDLISELSQPETFDMAFNTRSEPLRVVKSSIVCGVRSRIGALTSAGKVIPFTPIGLPVLDSGFVTAKSFL
Tropheryma whipplei Dig7        TIEVNNDLISELSQPETFDMAFNTRSEPLRVVKSSIVCGVRSRIGALTSAGKVIPFTPIGLPVLDSGFVTAKSFL
Tropheryma whipplei Dig9        TIEVNNDLISELSQPETFDMAFNTHSEPLRVVKSSIVCGVRSRIGALTSAGKVIPFTPIGLPVLDSGFVTAKSFL
Tropheryma whipplei Dig15       TIEVNNDLISELSQPETFDMAFNTHSEPLRVVKSSIVCGVRSRIGALTSAGKVIPFTPIGLPVLDSGFVTAKSFL
Tropheryma whipplei Neuro14     TIEVNNDLISELSQPETFDMAFNTHSEPLRVVKSSIVCGVRSRIGALTSAGKVIPFTPIGLPVLDSGFVTAKSFL
Tropheryma whipplei Twist       TIEVNNDLISELSQPETFDMAFNTHSEPLRVVKSSIVCGVRSRIGALTSAGKVIPFTPIGLPVLDSGFVTAKSFL
Tropheryma whipplei Dig10       TIEVNNDLISELSQPETFDMAFNTHSEPLRVVKSSIVCGVRSRIGALTSAGKVIPFTPIGLPVLDSGFVTAKSFL
Tropheryma whipplei Art1        TIEVNNDLISELSQPETFDMAFNTHSEPLRVVKSSIVCGVRSRIGALTSAGKVIPFTPIGLPVLDSGFVTAKSFL
Tropheryma whipplei Neuro20     TIEVNNDLISELSQPETFDMAFNTHSEPLRVVKSSIVCGVRSRIGALTSAGKVIPFTPIGLPVLDSGFVTAKSFL
Tropheryma whipplei Neuro1      TIEVNNDLISELSQPETFDMTFNTHSEPLRVVKSSIVCGVRSRIGALTSAGKVIPFTPIGLPVLDSGFVTAKSFL
Tropheryma whipplei Bcu26       TIEVNNDLISELSQPETFDMTFNTHSEPLRVVKSSIVCGVRSRIGALTSAGKVIPFTPIGLPVLDSGFVTAKSFL
Tropheryma whipplei TW08/27     TIEVNNDLISELSQPETFDMTFNTHSEPLRVVKSSIVCGVRSRIGALTSAGKVIPFTPIGLPVLDSGFVTAKSFL
Tropheryma whipplei shenzhen2   TIEVNNDLISELSQPETFDMTFNTHSEPLRVVKSSIVCGVRSRIGALTSAGKVIPFTPIGLPVLDSGFVTAKSFL
Tropheryma whipplei shenzhen1   TIEVNNDLISELSQPETFDMTFNTHSEPLRVVKSSIVCGVRSRIGALTSAGKVIPFTPIGLPVLDSGFVTAKSFL
```

```
                                       610       620       630       640       650       660       670
Tropheryma whipplei Art29      GLENDEEAVLLFELHQKESLAIATSSGIVKRVTPAIWPDRTERVITLSAGDRVVGAARGVSSCGYFVFITAKGNL
Tropheryma whipplei Pneumo30   GLENDEEAVLLFELHQKESLAIATSSGIVKRVTPAIWPDRTERVITLSAGDRVVGAARGVSSCGYFVFITAKGNL
Tropheryma whipplei Endo32     GLENDEEAVLLFELHQKESLAIATSSGIVKRVTPAIWPDRTERVITLSAGDRVVGAARGVSSCGYFVFITAKGNL
Tropheryma whipplei slow2      GLENDEEAVLLFELHQKESLAIATSSGIVKRVTPAIWPDRTERVITLSAGDRVVGAARGVSSCGYFVFITAKGNL
Tropheryma whipplei Sali28     GLENDEEAVLLFELHQKESLAIATSSGIVKRVTPAIWPDRTERVITLSAGDRVVGAARGVSSCGYFVFITAKGNL
Tropheryma whipplei Endo27     GLENDEEAVLLFELHQKESLAIATSSGIVKRVTPAIWPDRTERVITLSAGDRVVGAARGVSSCGYFVFITAKGNL
Tropheryma whipplei DigMusc17  GLENDEEAVLLFELHQKESLAIATSSGIVKRVTPAIWPDRTERVITLSAGDRVVGAARGVSSCGYFVFITAKGNL
Tropheryma whipplei DigADP25   GLENDEEAVLLFELHQKESLAIATSSGIVKRVTPAIWPDRTERVITLSAGDRVVGAARGVSSCGYFVFITAKGNL
Tropheryma whipplei Dig7       GLENDEEAVLLFELHQKESLAIATSSGIVKRVTPAIWPDRTERVITLSAGDRVVGAARGVSSCGYFVFITAKGNL
Tropheryma whipplei Dig9       GLENDEEAVLLFELHQKESLAIATSSGIVKRVTPAIWPDRTERVITLSAGDRVVGAARGVSSCGYFVFITAKGNL
Tropheryma whipplei Dig15      GLENDEEAVLLFELHQKESLAIATSSGIVKRVTPAIWPDRTERVITLSAGDRVVGAARGVSSCGYFVFITAKGNL
Tropheryma whipplei Neuro14    GLENDEEAVLLFELHQKESLAIATSSGIVKRVTPAIWPDRTERVITLSAGDRVVGAARGVSSCGYFVFITAKGNL
Tropheryma whipplei Twist      GLENDEEAVLLFELHQKESLAIATSSGIVKRVTPAIWPDRTERVITLSAGDRVVGAARGVSSCGYFVFITAKGNL
Tropheryma whipplei Dig10      GLENDEEAVLLFELHQKESLAIATSSGIVKRVTPAIWPDRTERVITLSAGDRVVGAARGVSSCGYFVFITAKGNL
Tropheryma whipplei Art1       GLENDEEAVLLFELHQKESLAIATSSGIVKRVTPAIWPDRTERVITLSAGDRVVGAARGVSSCGYFVFITAKGNL
Tropheryma whipplei Neuro20    GLENDEEAVLLFELHQKESLAIATSSGIVKRVTPAIWPDRTERVITLSAGDRVVGAARGVSSCGYFVFITAKGNL
Tropheryma whipplei Neuro1     GLENDEEAVLLFELHQKESLAIATSSGIVKRVTPAIWPDRTERVITLSAGDRVVGAARGVSSCGYFVFITAKGNL
Tropheryma whipplei Bcu26      GLENDEEAVLLFELHQKESLAIATSSGIVKRVTPAIWPDRTERVITLSAGDRVVGAARGVSSCGYFVFITAKGNL
Tropheryma whipplei TW08/27    GLENDEEAVLLFELHQKESLAIATSSGIVKRVTPAIWPDRTERVITLSAGDRVVGAARGVSSCGYFVFITAKGNL
Tropheryma whipplei shenzhen2  GLENDEEAVLLFELHQKESLAIATSSGIVKRVTPAIWPDRTERVITLSAGDRVVGAARGVSSCGYFVFITAKGNL
Tropheryma whipplei shenzhen1  GLENDEEAVLLFELHQKESLAIATSSGIVKRVTPAIWPDRTERVITLSAGDRVVGAARGVSSCGYFVFITAKGNL

                                    680       690       700       710       720       730       740       750
Tropheryma whipplei Art29      LHYSAGLVNPQNRPARGVAGVTLKDDDDRVISFGYTEDVDRANVLTVSKRMTTSRLSSYQTRGRATRGVQCHKFL
Tropheryma whipplei Pneumo30   LHYSAGLVNPQNRPARGVAGVTLKDDDDRVISFGYTEDVDRANVLTVSKRMTTSRLSSYQTRGRATRGVQCHKFL
Tropheryma whipplei Endo32     LHYSAGLVNPQNRPARGVAGVTLKDDDDRVISFGYTEDVDRANVLTVSKRMTTSRLSSYQTRGRATRGVQCHKFL
Tropheryma whipplei slow2      LHYSAGLVNPQNRPARGVAGVTLKDDDDRVISFGYTEDVDRANVLTVSKRMTTSRLSSYQTRGRATRGVQCHKFL
Tropheryma whipplei Sali28     LHYSAGLVNPQNRPARGVAGVTLKDDDDRVISFGYTEDVDRANVLTVSKRMTTSRLSSYQTRGRATRGVQCHKFL
Tropheryma whipplei Endo27     LHYSAGLVNPQNRPARGVAGVTLKDDDDRVISFGYTEDVDRANVLTVSKRMTTSRLSSYQTRGRATRGVQCHKFL
Tropheryma whipplei DigMusc17  LHYSAGLVNPQNRPARGVAGVTLKDDDDRVISFGYTEDVDRANVLTVSKRMTTSRLSSYQTRGRATRGVQCHKFL
Tropheryma whipplei DigADP25   LHYSAGLVNPQNRPARGVAGVTLKDDDDRVISFGYTEDVDRANVLTVSKRMTTSRLSSYQTRGRATRGVQCHKFL
Tropheryma whipplei Dig7       LHYSAGLVNPQNRPARGVAGVTLKDDDDRVISFGYTEDVDRANVLTVSKRMTTSRLSSYQTRGRATRGVQCHKFL
Tropheryma whipplei Dig9       LHYSAGLVNPQNRPARGVAGVTLKDDDDRVISFGYTEDVDRANVLTVSKRMTTSRLSSYQTRGRATRGVQCHKFL
Tropheryma whipplei Dig15      LHYSAGLVNPQNRPARGVAGVTLKDDDDRVISFGYTEDVDRANVLTVSKRMTTSRLSSYQTRGRATRGVQCHKFL
Tropheryma whipplei Neuro14    LHYSAGLVNPQNRPARGVAGVTLKDDDDRVISFGYTEDVDRANVLTVSKRMTTSRLSSYQTRGRATRGVQCHKFL
Tropheryma whipplei Twist      LHYSAGLVNPQNRPARGVAGVTLKDDDDRVISFGYTEDVDRANVLTVSKRMTTSRLSSYQTRGRATRGVQCHKFL
Tropheryma whipplei Dig10      LHYSAGLVNPQNRPARGVAGVTLKDDDDRVISFGYTEDVDRANVLTVSKRMTTSRLSSYQTRGRATRGVQCHKFL
Tropheryma whipplei Art1       LHYSAGLVNPQNRPARGVAGVTLKDDDDRVISFGYTEDVDRANVLTVSKRMTTSRLSSYQTRGRATRGVQCHKFL
Tropheryma whipplei Neuro20    LHYSAGLVNPQNRPARGVAGVTLKDDDDRVISFGYTEDVDRANVLTVSKRMTTSRLSSYQTRGRATRGVQCHKFL
Tropheryma whipplei Neuro1     LHYSAGLVNPQNRPARGVAGVTLKDDDDRVISFGYTEDVDRANVLTVSKRMTTSRLSSYQTRGRATRGVQCHKFL
Tropheryma whipplei Bcu26      LHYSAGLVNPQNRPARGVAGVTLKDDDDRVISFGYTEDVDRANVLTVSKRMTTSRLSSYQTRGRATRGVQCHKFL
Tropheryma whipplei TW08/27    LHYSAGLVNPQNRPARGVAGVTLKDDDDRVISFGYTEDVDRANVLTVSKRMTTSRLSSYQTRGRATRGVQCHKFL
Tropheryma whipplei shenzhen2  LHYSAGLVNPQNRPARGVAGVTLKDDDDRVISFGYTEDVDRANVLTVSKRMTTSRLSSYQTRGRATRGVQCHKFL
Tropheryma whipplei shenzhen1  LHYSAGLVNPQNRPARGVAGVTLKDDDDRVISFGYTEDVDRANVLTVSKRMTTSRLSSYQTRGRATRGVQCHKFL

                                       760       770       780       790       800       810       820
Tropheryma whipplei Art29      KGEEWLHAAIVGENLAAIDNLGNVVAIGKSSVTAKSCAEVKQYRLPQEKSGKSKREDRLIRYLGYPFVHGNDTY
Tropheryma whipplei Pneumo30   KGEEWLHAAIVGENLAAIDNLGNVVAIGKSSVTAKSCAGVKQYRLPQEKSGKSKREDRLIRYLGYPFVHGNDTY
Tropheryma whipplei Endo32     KGEEWLHAAIVGENLAAIDNLGNVVAIGKSSVTAKSCAGVKQYRLPQEKSGKSKREDRLIRYLGYPFVHGNDTY
Tropheryma whipplei slow2      KGEEWLHAAIVGENLAAIDNLGNVVAIGKSSVTAKSCAGVKQYRLPQEKSGKSKREDRLIRYLGYPFVHGNDTY
Tropheryma whipplei Sali28     KGEEWLHAAIVGENLAAIDNLGNVVAIGKSSVTAKSCAGVKQYRLPQEKSGKSKREDRLIRYLGYPFVHGNDTY
Tropheryma whipplei Endo27     KGEEWLHAAIVGENLAAIDNLGNVVAIGKSSVTAKSCAGVKQYRLPQEKSGKSKREDRLIRYLGYPFVHGNDTY
Tropheryma whipplei DigMusc17  KGEEWLHAAIVGENLAAIDNLGNVVAIGKSSVTAKSCAGVKQYRLPQEKSGKSKREDRLIRYLGYPFVHGNDTY
Tropheryma whipplei DigADP25   KGEEWLHAAIVGENLAAIDNLGNVVAIGKSSVTAKSCAGVKQYRLPQEKSGKSKREDRLIRYLGYPFVHGNDTY
Tropheryma whipplei Dig7       KGEEWLHAAIVGENLAAIDNLGNVVAIGKSSVTAKSCAGVKQYRLPQEKSGKSKREDRLIRYLGYPFVHGNDTY
Tropheryma whipplei Dig9       KGEEWLHAAIVGENLAAIDNLGNVVAIGKSSVTAKSCAGVKQYRLPQEKSGKSKREDRLIRYLGYPFVHGNDTY
Tropheryma whipplei Dig15      KGEEWLHAAIVGENLAAIDNLGNVVAIGKSSVTAKSCAGVKQYRLPQEKSGKSKREDRLIRYLGYPFVHGNDTY
Tropheryma whipplei Neuro14    KGEEWLHAAIVGENLAAIDNLGNVVAIGKSSVTAKSCAGVKQYRLPQEKSGKSKREDRLIRYLGYPFVHGNDTY
Tropheryma whipplei Twist      KGEEWLHAAIVGENLAAIDNLGNVVAIGKSSVTAKSCAGVKQYRLPQEKSGKSKREDRLIRYLGYPFVHGNDTY
Tropheryma whipplei Dig10      KGEEWLHAAIVGENLAAIDNLGNVVAIGKSSVTAKSCAGVKQYRLPQEKSGKSKREDRLIRYLGYPFVHGNDTY
Tropheryma whipplei Art1       KGEEWLHAAIVGENLAAIDNLGNVVAIGKSSVTAKSCAGVKQYRLPQEKSGKSKREDRLIRYLGYPFVHGNDTY
Tropheryma whipplei Neuro20    KGEEWLHAAIVGENLAAIDNLGNVVAIGKSSVTAKSCAGVKQYRLPQEKSGKSKREDRLIRYLGYPFVHGNDTY
Tropheryma whipplei Neuro1     KGEEWLHAAIVGENLAAIDNLGNVVAIGKSSVTAKSCAGVKQYRLPQEKSGKSKREDRLIRYLGYPFVHGNDTY
Tropheryma whipplei  Bcu26     KGEEWLHAAIVGENLAAIDNLGNVVAIGKSSVTAKSCAGVKQYRLPQEKSGKSKREDRLIRYLGYPFVHGNDTY
Tropheryma whipplei TW08/27    KGEEWLHAAIVGENLAAIDNLGNVVAIGKSSVTAKSCAGVKQYRLPQEKSGKSKREDRLIRYLGYPFVHGNDTY
Tropheryma whipplei shenzhen2  KGEEWLHAAIVGENLAAIDNLGNVVAIGKSSVTAKSCAGVKQYRLPQEKSGKSKREDRLIRYLGYPFVHGNDTY
Tropheryma whipplei shenzhen1  KGEEWLHAAIVGENLAAIDNLGNVVAIGKSSVTAKSCAGVKQYRLPQEKSGKSKREDRLIRYLGYPFVHGNDTY
```
