# Supplementary material for: Genomic characterization of two metagenome-assembled genomes of Tropheryma whipplei from China
Source: Front Cell Infect Microbiol. 2022 Sep 16;12:947486. doi: 10.3389/fcimb.2022.947486 (PMC9523146; doi:10.3389/fcimb.2022.947486)
Supplement: Supplementary file 3 [file DataSheet_3.pdf]

```
                                       1        10        20        30        40        50        60        70
Tropheryma whipplei Dig9       MSSNTDYNADDIALLEGLAAVRKRPGMYIGSTDNHGLAHCLWEIIDNSVDEVLAGFGNVVNITLHGDNSFSVQDF
Tropheryma whipplei Art1       MSSNTDYNADDIALLEGLAAVRKRPGMYIGSTDNHGLAHCLWEIIDNSVDEVLAGFGNVVNITLHGDNSFSVQDF
Tropheryma whipplei Neuro14    MSSNTDYNADDIALLEGLAAVRKRPGMYIGSTDNHGLAHCLWEIIDNSVDEVLAGFGNVVNITLHGDNSFSVQDF
Tropheryma whipplei Dig15      MSSNTDYNADDIALLEGLAAVRKRPGMYIGSTDNHGLAHCLWEIIDNSVDEVLAGFGNVVNITLHGDNSFSVQDF
Tropheryma whipplei Dig10      MSSNTDYNADDIALLEGLAAVRKRPGMYIGSTDNHGLAHCLWEIIDNSVDEVLAGFGNVVNITLHGDNSFSVQDF
Tropheryma whipplei Twist      MSSNTDYNADDIALLEGLAAVRKRPGMYIGSTDNHGLAHCLWEIIDNSVDEVLAGFGNVVNITLHGDNSFSVQDF
Tropheryma whipplei Neuro20    MSSNTDYNADDIALLEGLAAVRKRPGMYIGSTDNHGLAHCLWEIIDNSVDEVLAGFGNVVNITLHGDNSFSVQDF
Tropheryma whipplei TW08/27    MSSNTDYNADDIALLEGLAAVRKRPGMYIGSTDNHGLAHCLWEIIDNSVDEVLAGFGNVVNITLHGDNSFSVQDF
Tropheryma whipplei slow2      MSSNTDYNADDIALLEGLAAVRKRPGMYIGSTDNHGLAHCLWEIIDNSVDEVLAGFGNVVNITLHGDNSFSVQDF
Tropheryma whipplei Sali28     MSSNTDYNADDIALLEGLAAVRKRPGMYIGSTDNHGLAHCLWEIIDNSVDEVLAGFGNVVNITLHGDNSFSVQDF
Tropheryma whipplei DigMusc17  MSSNTDYNADDIALLEGLAAVRKRPGMYIGSTDNHGLAHCLWEIIDNSVDEVLAGFGNVVNITLHGDNSFSVQDF
Tropheryma whipplei Endo27     MSSNTDYNADDIALLEGLAAVRKRPGMYIGSTDNHGLAHCLWEIIDNSVDEVLAGFGNVVNITLHGDNSFSVQDF
Tropheryma whipplei  Dig7      MSSNTDYNADDIALLEGLAAVRKRPGMYIGSTDNHGLAHCLWEIIDNSVDEVLAGFGNVVNITLHGDNSFSVQDF
Tropheryma whipplei Bcu26      MSSNTDYNADDIALLEGLAAVRKRPGMYIGSTDNHGLAHCLWEIIDNSVDEVLAGFGNVVNITLHGDNSFSVQDF
Tropheryma whipplei Art29      MSSNTDYNADDIALLEGLAAVRKRPGMYIGSTDNHGLAHCLWEIIDNSVDEVLAGFGNVVNITLHGDNSFSVQDF
Tropheryma whipplei Pneumo30   MSSNTDYNADDIALLEGLAAVRKRPGMYIGSTDNHGLAHCLWEIIDNSVDEVLAGFGNVVNITLHGDNSFSVQDF
Tropheryma whipplei Neuro1     MSSNTDYNADDIALLEGLAAVRKRPGMYIGSTDNHGLAHCLWEIIDNSVDEVLAGFGNVVNITLHGDNSFSVQDF
Tropheryma whipplei Endo32     MSSNTDYNADDIALLEGLAAVRKRPGMYIGSTDNHGLAHCLWEIIDNSVDEVLAGFGNVVNITLHGDNSFSVQDF
Tropheryma whipplei DigADP25   MSSNTDYNADDIALLEGLAAVRKRPGMYIGSTDNHGLAHCLWEIIDNSVDEVLAGFGNVVNITLHGDNSFSVQDF
Tropheryma whipplei shenzhen2  MSSNTDYNADDIALLEGLAAVRKRPGMYIGSTDNHGLAHCLWEIIDNSVDEVLAGFGNVVNITLHGDNSFSVQDF
Tropheryma whipplei shenzhen1  MSSNTDYNADDIALLEGLAAVRKRPGMYIGSTDNHGLAHCLWEIIDNSVDEVLAGFGNVVNITLHGDNSFSVQDF
```

```
                                   80        90       100       110       120       130       140       150
Tropheryma whipplei Dig9       GRGIPVDIESKTGLTGVELVFTKLHAGGKFGTGAYFTSGGLHGVGASVVNALSEKLSVEVDRDGMTWVMEFRNQV
Tropheryma whipplei Art1       GRGIPVDIESKTGLTGVELVFTKLHAGGKFGTGAYFTSGGLHGVGASVVNALSEKLSVEVDRDGMTWVMEFRNQV
Tropheryma whipplei Neuro14    GRGIPVDIESKTGLTGVELVFTKLHAGGKFGTGAYFTSGGLHGVGASVVNALSEKLSVEVDRDGMTWVMEFRNQV
Tropheryma whipplei Dig15      GRGIPVDIESKTGLTGVELVFTKLHAGGKFGTGAYFTSGGLHGVGASVVNALSEKLSVEVDRDGMTWVMEFRNQV
Tropheryma whipplei Dig10      GRGIPVDIESKTGLTGVELVFTKLHAGGKFGTGAYFTSGGLHGVGASVVNALSEKLSVEVDRDGMTWVMEFRNQV
Tropheryma whipplei Twist      GRGIPVDIESKTGLTGVELVFTKLHAGGKFGTGAYFTSGGLHGVGASVVNALSEKLSVEVDRDGMTWVMEFRNQV
Tropheryma whipplei Neuro20    GRGIPVDIESKTGLTGVELVFTKLHAGGKFGTGAYFTSGGLHGVGASVVNALSEKLSVEVDRDGMTWVMEFRNQV
Tropheryma whipplei TW08/27    GRGIPVDIESKTGLTGVELVFTKLHAGGKFGTGAYFTSGGLHGVGASVVNALSEKLSVEVDRDGMTWVMEFRNQV
Tropheryma whipplei slow2      GRGIPVDIESKTGLTGVELVFTKLHAGGKFGTGAYFTSGGLHGVGASVVNALSEKLSVEVDRDGMTWVMEFRNQV
Tropheryma whipplei Sali28     GRGIPVDIESKTGLTGVELVFTKLHAGGKFGTGAYFTSGGLHGVGASVVNALSEKLSVEVDRDGMTWVMEFRNQV
Tropheryma whipplei DigMusc17  GRGIPVDIESKTGLTGVELVFTKLHAGGKFGTGAYFTSGGLHGVGASVVNALSEKLSVEVDRDGMTWVMEFRNQV
Tropheryma whipplei Endo27     GRGIPVDIESKTGLTGVELVFTKLHAGGKFGTGAYFTSGGLHGVGASVVNALSEKLSVEVDRDGMTWVMEFRNQV
Tropheryma whipplei Dig7       GRGIPVDIESKTGLTGVELVFTKLHAGGKFGTGAYFTSGGLHGVGASVVNALSEKLSVEVDRDGMTWVMEFRNQV
Tropheryma whipplei Bcu26      GRGIPVDIESKTGLTGVELVFTKLHAGGKFGTGAYFTSGGLHGVGASVVNALSEKLSVEVDRDGMTWVMEFRNQV
Tropheryma whipplei Art29      GRGIPVDIESKTGLTGVELVFTKLHAGGKFGTGAYFTSGGLHGVGASVVNALSEKLSVEVDRDGMTWVMEFRNQV
Tropheryma whipplei Pneumo30   GRGIPVDIESKTGLTGVELVFTKLHAGGKFGTGAYFTSGGLHGVGASVVNALSEKLSVEVDRDGMTWVMEFRNQV
Tropheryma whipplei Neuro1     GRGIPVDIESKTGLTGVELVFTKLHAGGKFGTGAYFTSGGLHGVGASVVNALSEKLSVEVDRDGMTWVMEFRNQV
Tropheryma whipplei Endo32     GRGIPVDIESKTGLTGVELVFTKLHAGGKFGTGAYFTSGGLHGVGASVVNALSEKLSVEVDRDGMTWVMEFRNQV
Tropheryma whipplei DigADP25   GRGIPVDIESKTGLTGVELVFTKLHAGGKFGTGAYFTSGGLHGVGASVVNALSEKLSVEVDRDGMTWVMEFRNQV
Tropheryma whipplei shenzhen2  GRGIPVDIESKTGLTGVELVFTKLHAGGKFGTGAYFTSGGLHGVGASVVNALSEKLSVEVDRDGMTWVMEFRNQV
Tropheryma whipplei shenzhen1  GRGIPVDIESKTGLTGVELVFTKLHAGGKFGTGAYFTSGGLHGVGASVVNALSEKLSVEVDRDGMTWVMEFRNQV
```

```
                                       160       170       180       190       200       210       220
Tropheryma whipplei Dig9       TSGLRSKCKIASNVTGTRVRFWPDPLIFGSAEICIESLHQRAMQTAFLVPGLRVNIEDRVLGKSSSYLFEGGCKQ
Tropheryma whipplei Art1       TSGLRSKCKIASNVTGTRVRFWPDPLIFGSAEICIESLHQRAMQTAFLVPGLRVNIEDRVLGKSSSYLFEGGCKQ
Tropheryma whipplei Neuro14    TSGLRSKCKIASNVTGTRVRFWPDPLIFGSAEICIESLHQRAMQTAFLVPGLRVNIEDRVLGKSSSYLFEGGCKQ
Tropheryma whipplei Dig15      TSGLRSKCKIASNVTGTRVRFWPDPLIFGSAEICIESLHQRAMQTAFLVPGLRVNIEDRVLGKSSSYLFEGGCKQ
Tropheryma whipplei Dig10      TSGLRSKCKIASNVTGTRVRFWPDPLIFGSAEICIESLHQRAMQTAFLVPGLRVNIEDRVLGKSSSYLFEGGCKQ
Tropheryma whipplei Twist      TSGLRSKCKIASNVTGTRVRFWPDPLIFGSAEICIESLHQRAMQTAFLVPGLRVNIEDRVLGKSSSYLFEGGCKQ
Tropheryma whipplei Neuro20    TSGLRSKCKIASNVTGTRVRFWPDPLIFGSAEICIESLHQRAMQTAFLVPGLRVNIEDRVLGKSSSYLFEGGCKQ
Tropheryma whipplei TW08/27    TSGLRSKCKIASNVTGTRVRFWPDPLIFGSAEICIESLHQRAMQTAFLVPGLRVNIEDRVLGKSSSYLFEGGCKQ
Tropheryma whipplei slow2      TSGLRSKCKIASNVTGTRVRFWPDPLIFGSAEICIESLHQRAMQTAFLVPGLRVNIEDRVLGKSSSYLFEGGCKQ
Tropheryma whipplei Sali28     TSGLRSKCKIASNVTGTRVRFWPDPLIFGSAEICIESLHQRAMQTAFLVPGLRVNIEDRVLGKSSSYLFEGGCKQ
Tropheryma whipplei DigMusc17  TSGLRSKCKIASNVTGTRVRFWPDPLIFGSAEICIESLHQRAMQTAFLVPGLRVNIEDRVLGKSSSYLFEGGCKQ
Tropheryma whipplei Endo27     TSGLRSKCKIASNVTGTRVRFWPDPLIFGSAEICIESLHQRAMQTAFLVPGLRVNIEDRVLGKSSSYLFEGGCKQ
Tropheryma whipplei Dig7       TSGLRSKCKIASNVTGTRVRFWPDPLIFGSAEICIESLHQRAMQTAFLVPGLRVNIEDRVLGKSSSYLFEGGCKQ
Tropheryma whipplei Bcu26      TSGLRSKCKIASNVTGTRVRFWPDPLIFGSAEICIESLHQRAMQTAFLVPGLRVNIEDRVLGKSSSYLFEGGCKQ
Tropheryma whipplei Art29      TSGLRSKCKIASNVTGTRVRFWPDPLIFGSAEICIESLHQRAMQTAFLVPGLRVNIEDRVLGKSSSYLFEGGCKQ
Tropheryma whipplei Pneumo30   TSGLRSKCKIASNVTGTRVRFWPDPLIFGSAEICIESLHQRAMQTAFLVPGLRVNIEDRVLGKSSSYLFEGGCKQ
Tropheryma whipplei Neuro1     TSGLRSKCKIASNVTGTRVRFWPDPLIFGSAEICIESLHQRAMQTAFLVPGLRVNIEDRVLGKSSSYLFEGGCKQ
Tropheryma whipplei Endo32     TSGLRSKCKIASNVTGTRVRFWPDPLIFGSAEICIESLHQRAMQTAFLVPGLRVNIEDRVLGKSSSYLFEGGCKQ
Tropheryma whipplei DigADP25   TSGLRSKCKIASNVTGTRVRFWPDPLIFGSAEICIESLHQRAMQTAFLVPGLRVNIEDRVLGKSSSYLFEGGCKQ
Tropheryma whipplei shenzhen2  TSGLRSKCKIASNVTGTRVRFWPDPLIFGSAEICIESLHQRAMQTAFLVPGLRVNIEDRVLGKSSSYLFEGGCKQ
Tropheryma whipplei shenzhen1  TSGLRSKCKIASNVTGTRVRFWPDPLIFGSAEICIESLHQRAMQTAFLVPGLRVNIEDRVLGKSSSYLFEGGCKQ
```

```
                                   230       240       250       260       270       280       290       300
Tropheryma whipplei Dig9       FVEHLSAGELITDVLCIRGKGSFSEAVPVLKETGNLVISSVERVCDVDIALAWRNDFETHINSFVNVIETPKGGT
Tropheryma whipplei Art1       FVEHLSAGELITDVLCIRGKGSFSEAVPVLKETGNLVISSVERVCDVDIALAWRNDFETHINSFVNVIETPKGGT
Tropheryma whipplei Neuro14    FVEHLSAGELITDVLCIRGKGSFSEAVPVLKETGNLVISSVERVCDVDIALAWRNDFETHINSFVNVIETPKGGT
Tropheryma whipplei Dig15      FVEHLSAGELITDVLCIRGKGSFSEAVPVLKETGNLVISSVERVCDVDIALAWRNDFETHINSFVNVIETPKGGT
Tropheryma whipplei Dig10      FVEHLSAGELITDVLCIRGKGSFSEAVPVLKETGNLVISSVERVCDVDIALAWRNDFETHINSFVNVIETPKGGT
Tropheryma whipplei Twist      FVEHLSAGELITDVLCIRGKGSFSEAVPVLKETGNLVISSVERVCDVDIALAWRNDFETHINSFVNVIETPKGGT
Tropheryma whipplei Neuro20    FVEHLSAGELITDVLCIRGKGSFSEAVPVLKETGNLVISSVERVCDVDIALAWRNDFETHINSFVNVIETPKGGT
Tropheryma whipplei TW08/27    FVEHLSAGELITDVLCIRGKGSFSEAVPVLKETGNLVISSVERVCDVDIALAWRNDFETHINSFVNVIETPKGGT
Tropheryma whipplei slow2      FVEHLSAGELITDVLCIRGKGSFSEAVPVLKETGNLVISSVERVCDVDIALAWRNDFETHINSFVNVIETPKGGT
Tropheryma whipplei Sali28     FVEHLSAGELITDVLCIRGKGSFSEAVPVLKETGNLVISSVERVCDVDIALAWRNDFETHINSFVNVIETPKGGT
Tropheryma whipplei DigMusc17  FVEHLSAGELITDVLCIRGKGSFSEAVPVLKETGNLVISSVERVCDVDIALAWRNDFETHINSFVNVIETPKGGT
Tropheryma whipplei Endo27     FVEHLSAGELITDVLCIRGKGSFSEAVPVLKETGNLVISSVERVCDVDIALAWRNDFETHINSFVNVIETPKGGT
Tropheryma whipplei Dig7       FVEHLSAGELITDVLCIRGKGSFSEAVPVLKETGNLVISSVERVCDVDIALAWRNDFETHINSFVNVIETPKGGT
Tropheryma whipplei Bcu26      FVEHLSAGELITDVLCIRGKGSFSEAVPVLKETGNLVISSVERVCDVDIALAWRNDFETHINSFVNVIETPKGGT
Tropheryma whipplei Art29      FVEHLSAGELITDVLCIRGKGSFSEAVPVLKETGNLVISSVERVCDVDIALAWRNDFETHINSFVNVIETPKGGT
Tropheryma whipplei Pneumo30   FVEHLSAGELITDVLCIRGKGSFSEAVPVLKETGNLVISSVERVCDVDIALAWRNDFETHINSFVNVIETPKGGT
Tropheryma whipplei Neuro1     FVEHLSAGELITDVLCIRGKGSFSEAVPVLKETGNLVISSVERVCDVDIALAWRNDFETHINSFVNVIETPKGGT
Tropheryma whipplei Endo32     FVEHLSAGELITDVLCIRGKGSFSEAVPVLKETGNLVISSVERVCDVDIALAWRNDFETHINSFVNVIETPKGGT
Tropheryma whipplei DigADP25   FVEHLSAGELITDVLCIRGKGSFSEAVPVLKETGNLVISSVERVCDVDIALAWRNDFETHINSFVNVIETPKGGT
Tropheryma whipplei shenzhen2  FVEHLSAGELITDVLCIRGKGSFSEAVPVLKETGNLVISSVERVCDVDIALAWRNDFETHINSFVNVIETPKGGT
Tropheryma whipplei shenzhen1  FVEHLSAGELITDVLCIRGKGSFSEAVPVLKETGNLVISSVERVCDVDIALAWRNDFETHINSFVNVIETPKGGT
```

```
                                       310       320       330       340       350       360       370
Tropheryma whipplei Dig9       HYSGFEQALLKTIRNAVSSQSRYLKSGNNRPEREDVFSGLTAVVSVKLPEPQFEGQTKEVLGTPAVKGIVANIVA
Tropheryma whipplei Art1       HYSGFEQALLKTIRNAVSSQSRYLKSGNNRPEREDVFSGLTAVVSVKLPEPQFEGQTKEVLGTPAVKGIVANIVA
Tropheryma whipplei Neuro14    HYSGFEQALLKTIRNAVSSQSRYLKSGNNRPEREDVFSGLTAVVSVKLPEPQFEGQTKEVLGTPAVKGIVANIVA
Tropheryma whipplei Dig15      HYSGFEQALLKTIRNAVSSQSRYLKSGNNRPEREDVFSGLTAVVSVKLPEPQFEGQTKEVLGTPAVKGIVANIVA
Tropheryma whipplei Dig10      HYSGFEQALLKTIRNAVSSQSRYLKSGNNRPEREDVFSGLTAVVSVKLPEPQFEGQTKEVLGTPAVKGIVANIVA
Tropheryma whipplei Twist      HYSGFEQALLKTIRNAVSSQSRYLKSGNNRPEREDVFSGLTAVVSVKLPEPQFEGQTKEVLGTPAVKGIVANIVA
Tropheryma whipplei Neuro20    HYSGFEQALLKTIRNAVSSQSRYLKSGNNRPEREDVFSGLTAVVSVKLPEPQFEGQTKEVLGTPAVKGIVANIVA
Tropheryma whipplei TW08/27    HYSGFEQALLKTIRNAVSSQSRYLKSGNNRPEREDVFSGLTAVVSVKLPEPQFEGQTKEVLGTPAVKGIVANIVA
Tropheryma whipplei slow2      HYSGFEQALLKTIRNAVSSQSRYLKSGNNRPEREDVFSGLTAVVSVKLPEPQFEGQTKEVLGTPAVKGIVANIVA
Tropheryma whipplei Sali28     HYSGFEQALLKTIRNAVSSQSRYLKSGNNRPEREDVFSGLTAVVSVKLPEPQFEGQTKEVLGTPAVKGIVANIVA
Tropheryma whipplei DigMusc17  HYSGFEQALLKTIRNAVSSQSRYLKSGNNRPEREDVFSGLTAVVSVKLPEPQFEGQTKEVLGTPAVKGIVANIVA
Tropheryma whipplei Endo27     HYSGFEQALLKTIRNAVSSQSRYLKSGNNRPEREDVFSGLTAVVSVKLPEPQFEGQTKEVLGTPAVKGIVANIVA
Tropheryma whipplei Dig7       HYSGFEQALLKTIRNAVSSQSRYLKSGNNRPEREDVFSGLTAVVSVKLPEPQFEGQTKEVLGTPAVKGIVANIVA
Tropheryma whipplei Bcu26      HYSGFEQALLKTIRNAVSSQSRYLKSGNNRPEREDVFSGLTAVVSVKLPEPQFEGQTKEVLGTPAVKGIVANIVA
Tropheryma whipplei Art29      HYSGFEQALLKTIRNAVSSQSRYLKSGNNRPEREDVFSGLTAVVSVKLPEPQFEGQTKEVLGTPAVKGIVANIVA
Tropheryma whipplei Pneumo30   HYSGFEQALLKTIRNAVSSQSRYLKSGNNRPEREDVFSGLTALVSVKLPEPQFEGQTKEVLGTPAVKGIVANIVA
Tropheryma whipplei Neuro1     HYSGFEQALLKTIRNAVSSQSRYLKSGNNRPEREDVFSGLTAVVSVKLPEPQFEGQTKEVLGTPAVKGIVANIVA
Tropheryma whipplei Endo32     HYSGFEQALLKTIRNAVSSQSRYLKSGNNRPEREDVFSGLTAVVSVKLPEPQFEGQTKEVLGTPAVKGIVANIVA
Tropheryma whipplei DigADP25   HYSGFEQALLKTIRNAVSSQSRYLKSGNNRPEREDVFSGLTAVVSVKLPEPQFEGQTKEVLGTPAVKGIVANIVA
Tropheryma whipplei shenzhen2  HYSGFEQALLKTIRNAVSSQSRYLKSGNNRPEREDVFSGLTAVVSVKLPEPQFEGQTKEVLGTPAVKGIVANIVA
Tropheryma whipplei shenzhen1  HYSGFEQALLKTIRNAVSSQSRYLKSGNNRPEREDVFSGLTAVVSVKLPEPQFEGQTKEVLGTPAVKGIVANIVA
```

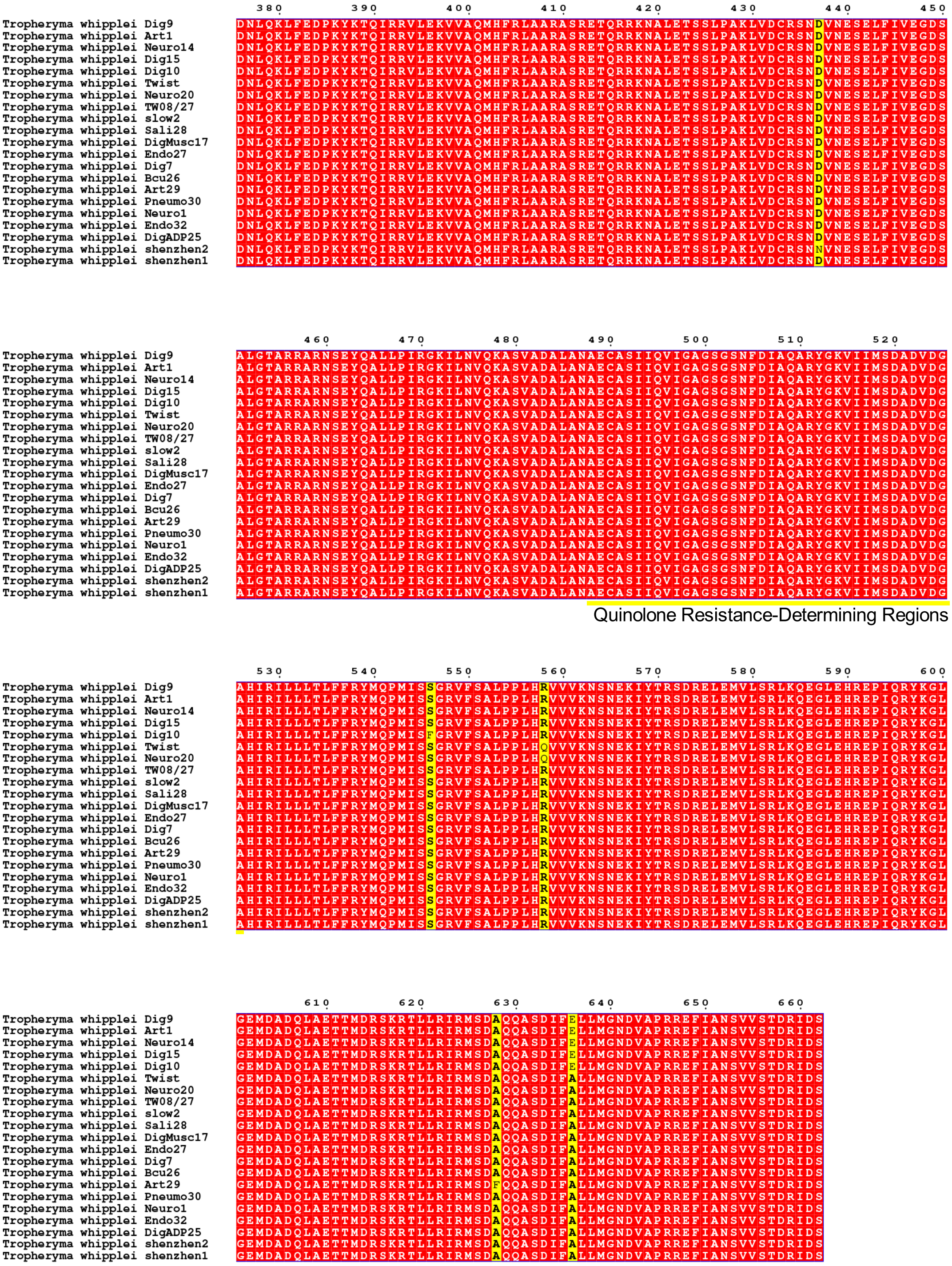
Quinolone Resistance-Determining Regions
