# Supplementary material for: Genomic characterization of two metagenome-assembled genomes of Tropheryma whipplei from China
Source: Front Cell Infect Microbiol. 2022 Sep 16;12:947486. doi: 10.3389/fcimb.2022.947486 (PMC9523146; doi:10.3389/fcimb.2022.947486)
Supplement: Supplementary file 4 [file DataSheet_4.pdf]

```
                                   1        10        20        30        40        50        60        70
Tropheryma whipplei Neuro14        MVDTPEPAYGADQIQVLEGLEAVRRRPGMYIGSTGSRGLHHLVYELVDNSVDEALAGYCTKINVAILADGGVRVT
Tropheryma whipplei Dig9           MVDTPEPAYGADQIQVLEGLEAVRRRPGMYIGSTGSRGLHHLVYELVDNSVDEALAGYCTKINVAILADGGVRVT
Tropheryma whipplei Dig15          MVDTPEPAYGADQIQVLEGLEAVRRRPGMYIGSTGSRGLHHLVYELVDNSVDEALAGYCTKINVAILADGGVRVT
Tropheryma whipplei Art1           MVDTPEPAYGADQIQVLEGLEAVRRRPGMYIGSTGSRGLHHLVYELVDNSVDEALAGYCTKINVAILADGGVRVT
Tropheryma whipplei TW08/27        MVDTPEPAYGADQIQVLEGLEAVRRRPGMYIGSTGSRGLHHLVYELVDNSVDEALAGYCTKINVAILADGGVRVT
Tropheryma whipplei Bcu26          MVDTPEPAYGADQIQVLEGLEAVRRRPGMYIGSTGSRGLHHLVYELVDNSVDEALAGYCTKINVAILADGGVRVT
Tropheryma whipplei Dig10          MVDTPEPAYGADQIQVLEGLEAVRRRPGMYIGSTGSRGLHHLVYELVDNSVDEALAGYCTKINVAILADGGVRVT
Tropheryma whipplei Neuro20        MVDTPEPAYGADQIQVLEGLEAVRRRPGMYIGSTGSRGLHHLVYELVDNSVDEALAGYCTKINVAILADGGVRVT
Tropheryma whipplei Neuro1         MVDTPEPAYGADQIQVLEGLEAVRRRPGMYIGSTGSRGLHHLVYELVDNSVDEALAGYCTKINVAILADGGVRVT
Tropheryma whipplei Twist          MVDTPEPAYGADQIQVLEGLEAVRRRPGMYIGSTGSRGLHHLVYELVDNSVDEALAGYCTKINVAILADGGVRVT
Tropheryma whipplei slow2          MVDTPEPAYGADQIQVLEGLEAVRRRPGMYIGSTGSRGLHHLVYELVDNSVDEALAGYCTKINVAILADGGVRVT
Tropheryma whipplei Sali28         MVDTPEPAYGADQIQVLEGLEAVRRRPGMYIGSTGSRGLHHLVYELVDNSVDEALAGYCTKINVAILADGGVRVT
Tropheryma whipplei DigMusc17      MVDTPEPAYGADQIQVLEGLEAVRRRPGMYIGSTGSRGLHHLVYELVDNSVDEALAGYCTKINVAILADGGVRVT
Tropheryma whipplei Endo27         MVDTPEPAYGADQIQVLEGLEAVRRRPGMYIGSTGSRGLHHLVYELVDNSVDEALAGYCTKINVAILADGGVRVT
Tropheryma whipplei DigADP25       MVDTPEPAYGADQIQVLEGLEAVRRRPGMYIGSTGSRGLHHLVYELVDNSVDEALAGYCTKINVAILADGGVRVT
Tropheryma whipplei Dig7           MVDTPEPAYGADQIQVLEGLEAVRRRPGMYIGSTGSRGLHHLVYELVDNSVDEALAGYCTKINVAILADGGVRVT
Tropheryma whipplei Art29          MVDTPEPAYGADQIQVLEGLEAVRRRPGMYIGSTGSRGLHHLVYELVDNSVDEALAGYCTKINVAILADGGVRVT
Tropheryma whipplei Pneumo30       MVDTPEPAYGADQIQVLEGLEAVRRRPGMYIGSTGSRGLHHLVYELVDNSVDEALAGYCTKINVAILADGGVRVT
Tropheryma whipplei Endo32         MVDTPEPAYGADQIQVLEGLEAVRRRPGMYIGSTGSRGLHHLVYELVDNSVDEALAGYCTKINVAILADGGVRVT
Tropheryma whipplei shenzhen2      MVDTPEPAYGADQIQVLEGLEAVRRRPGMYIGSTGSRGLHHLVYELVDNSVDEALAGYCTKINVAILADGGVRVT
Tropheryma whipplei shenzhen1      MVDTPEPAYGADQIQVLEGLEAVRRRPGMYIGSTGSRGLHHLVYELVDNSVDEALAGYCTKINVAILADGGVRVT
```

```
                                       80        90       100       110       120       130       140       150
Tropheryma whipplei Neuro14        DNGRGIPVDIHPKEGVSTVQVVLTTLHAGGKFGSGGYSVSGGLHGVGSSVVNALSKRLYVEVSRQGFVFRQNYEG
Tropheryma whipplei Dig9           DNGRGIPVDIHPKEGVSTVQVVLTTLHAGGKFGSGGYSVSGGLHGVGSSVVNALSKRLYVEVSRQGFVFRQNYEG
Tropheryma whipplei Dig15          DNGRGIPVDIHPKEGVSTVQVVLTTLHAGGKFGSGGYSVSGGLHGVGSSVVNALSKRLYVEVSRQGFVFRQNYEG
Tropheryma whipplei Art1           DNGRGIPVDIHPKEGVSTVQVVLTTLHAGGKFGSGGYSVSGGLHGVGSSVVNALSKRLYVEVSRQGFVFRQNYEG
Tropheryma whipplei TW08/27        DNGRGIPVDIHPKEGVSTVQVVLTTLHAGGKFGSGGYSVSGGLHGVGSSVVNALSKRLYVEVSRQGFVFRQNYEG
Tropheryma whipplei Bcu26          DNGRGIPVDIHPKEGVSTVQVVLTTLHAGGKFGSGGYSVSGGLHGVGSSVVNALSKRLYVEVSRQGFVFRQNYEG
Tropheryma whipplei Dig10          DNGRGIPVDIHPKEGVSTVQVVLTTLHAGGKFGSGGYSVSGGLHGVGSSVVNALSKRLYVEVSRQGFVFRQNYEG
Tropheryma whipplei Neuro20        DNGRGIPVDIHPKEGVSTVQVVLTTLHAGGKFGSGGYSVSGGLHGVGSSVVNALSKRLYVEVSRQGFVFRQNYEG
Tropheryma whipplei Neuro1         DNGRGIPVDIHPKEGVSTVQVVLTTLHAGGKFGSGGYSVSGGLHGVGSSVVNALSKRLYVEVSRQGFVFRQNYEG
Tropheryma whipplei Twist          DNGRGIPVDIHPKEGVSTVQVVLTTLHAGGKFGSGGYSVSGGLHGVGSSVVNALSKRLYVEVSRQGFVFRQNYEG
Tropheryma whipplei slow2          DNGRGIPVDIHPKEGVSTVQVVLTTLHAGGKFGSGGYSVSGGLHGVGSSVVNALSKRLYVEVSRQGFVFRQNYEG
Tropheryma whipplei Sali28         DNGRGIPVDIHPKEGVSTVQVVLTTLHAGGKFGSGGYSVSGGLHGVGSSVVNALSKRLYVEVSRQGFVFRQNYES
Tropheryma whipplei DigMusc17      DNGRGIPVDIHPKEGVSTVQVVLTTLHAGGKFGSGGYSVSGGLHGVGSSVVNALSKRLYVEVSRQGFVFRQNYEG
Tropheryma whipplei Endo27         DNGRGIPVDIHPKEGVSTVQVVLTTLHAGGKFGSGGYSVSGGLHGVGSSVVNALSKRLYVEVSRQGFVFRQNYEG
Tropheryma whipplei DigADP25       DNGRGIPVDIHPKEGVSTVQVVLTTLHAGGKFGSGGYSVSGGLHGVGSSVVNALSKRLYVEVSRQGFVFRQNYEG
Tropheryma whipplei Dig7           DNGRGIPVDIHPKEGVSTVQVVLTTLHAGGKFGSGGYSVSGGLHGVGSSVVNALSKRLYVEVSRQGFVFRQNYEG
Tropheryma whipplei Art29          DNGRGIPVDIHPKEGVSTVQVVLTTLHAGGKFGSGGYSVSGGLHGVGSSVVNALSKRLYVEVSRQGFVFRQNYEG
Tropheryma whipplei Pneumo30       DNGRGIPVDIHPKEGVSTVQVVLTTLHAGGKFGSGGYSVSGGLHGVGSSVVNALSKRLYVEVSRQGFVFRQNYEG
Tropheryma whipplei Endo32         DNGRGIPVDIHPKEGVSTVQVVLTTLHAGGKFGSGGYSVSGGLHGVGSSVVNALSKRLYVEVSRQGFVFRQNYEG
Tropheryma whipplei shenzhen2      DNGRGIPVDIHPKEGVSTVQVVLTTLHAGGKFGSGGYSVSGGLHGVGSSVVNALSKRLYVEVSRQGFVFRQNYEG
Tropheryma whipplei shenzhen1      DNGRGIPVDIHPKEGVSTVQVVLTTLHAGGKFGSGGYSVSGGLHGVGSSVVNALSKRLYVEVSRQGFVFRQNYEG
```

```
                                            160       170       180       190       200       210       220
Tropheryma whipplei Neuro14        GNPTAALERCEETNATGTAITFWPDDDIFETVCFEYEVLRSRLQQMAFLNSGLTVSITDERVDSPTNNETYCYQN
Tropheryma whipplei Dig9           GNPTAALERCEETNATGTAITFWPDDDIFETVCFEYEVLRSRLQQMAFLNSGLTVSITDERVDSPTNNETYCYQN
Tropheryma whipplei Dig15          GNPTAALERCEETNATGTAITFWPDDDIFETVCFEYEVLRSRLQQMAFLNSGLTVSITDERVDSPTNNETYCYQN
Tropheryma whipplei Art1           GNPTAALERCEETNATGTAITFWPDDDIFETVCFEYEVLRSRLQQMAFLNSGLTVSITDERVDSPTNNETYCYQN
Tropheryma whipplei TW08/27        GNPTAALERCEETNATGTAITFWPDDDIFETVCFEYEVLRSRLQQMAFLNSGLTVSITDERVDSPTNNETYCYQN
Tropheryma whipplei Bcu26          GNPTAALERCEETNATGTAITFWPDDDIFETVCFEYEVLRSRLQQMAFLNSGLTVSITDERVDSPTNNETYCYQN
Tropheryma whipplei Dig10          GNPTAALERCEETNATGTAITFWPDDDIFETVCFEYEVLRSRLQQMAFLNSGLTVSITDERVDSPTNNETYCYQN
Tropheryma whipplei Neuro20        GNPTAALERCEETNATGTAITFWPDDDIFETVCFEYEVLRSRLQQMAFLNSGLTVSITDERVDSPTNNETYCYQN
Tropheryma whipplei Neuro1         GNPTAALERCEETNATGTAITFWPDDDIFETVCFEYEVLRSRLQQMAFLNSGLTVSITDERVDSPTNNETYCYQN
Tropheryma whipplei Twist          GNPTAALERCEETNATGTAITFWPDDDIFETVCFEYEVLRSRLQQMAFLNSGLTVSITDERVDSPTNNETYCYQN
Tropheryma whipplei slow2          GNPTAALERCEETNATGTAITFWPDDDIFETVCFEYEVLRSRLQQMAFLNSGLTVSITDERVDSPTNNETYCYQN
Tropheryma whipplei Sali28         GNPTAALERCEETNATGTAITFWPDDDIFETVCFEYEVLRSRLQQMAFLNSGLTVSITDERVDSPTKSETYCYQN
Tropheryma whipplei DigMusc17      GNPTAALERCEETNATGTAITFWPDDDIFETVCFEYEVLRSRLQQMAFLNSGLTVSITDERVDSPTKSETYCYQN
Tropheryma whipplei Endo27         GNPTAALERCEETNATGTAITFWPDDDIFETVCFEYEVLRSRLQQMAFLNSGLTVSITDERVDSPTKSETYCYQN
Tropheryma whipplei DigADP25       GNPTAALERCEETNATGTAITFWPDDDIFETVCFEYEVLRSRLQQMAFLNSGLTVSITDERVDSPTKSETYCYQN
Tropheryma whipplei Dig7           GNPTAALERCEETNATGTAITFWPDDDIFETVCFEYEVLRSRLQQMAFLNSGLTVSITDERVDSPTKSETYCYQN
Tropheryma whipplei Art29          GNPTAALERCEETNATGTAITFWPDDDIFETVCFEYEVLRSRLQQMAFLNSGLTVSITDERVDSPTKSETYCYQN
Tropheryma whipplei Pneumo30       GNPTAALERCEETNATGTAITFWPDDDIFETVCFEYEVLRSRLQQMAFLNSGLTVSITDERVDSPTKSETYCYQN
Tropheryma whipplei Endo32         GNPTAALERCEETNATGTAITFWPDDDIFETVCFEYEVLRSRLQQMAFLNSGLTVSITDERVDSPTKSETYCYQN
Tropheryma whipplei shenzhen2      GNPTAALERCEETNATGTAITFWPDDDIFETVCFEYEVLRSRLQQMAFLNSGLTVSITDERVDSPAKSETYCYQN
Tropheryma whipplei shenzhen1      GNPTAALERCEETNATGTAITFWPDDDIFETVCFEYEVLRSRLQQMAFLNSGLTVSITDERVDSPTKSETYCYQN
```

```
                                       230       240       250       260       270       280       290       300
Tropheryma whipplei Neuro14        GLFDYVMYQNRIRKHEPLHEEIIHFQAENKDRLMSLEVAMRWTSSSTENIYTFANTINTHEGGTHEEGFRAALTS
Tropheryma whipplei Dig9           GLFDYVMYQNRIRKHEPLHEEIIHFQAENKDRLMSLEVAMRWTSSSTENIYTFANTINTHEGGTHEEGFRAALTS
Tropheryma whipplei Dig15          GLFDYVMYQNRIRKHEPLHEEIIHFQAENKDRLMSLEVAMRWTSSSTENIYTFANTINTHEGGTHEEGFRAALTS
Tropheryma whipplei Art1           GLFDYVMYQNRIRKHEPLHEEIIHFQAENKDRLMSLEVAMRWTSSSTENIYTFANTINTHEGGTHEEGFRAALTS
Tropheryma whipplei TW08/27        GLFDYVMYQNRIRKHEPLHEEIIHFQAENKDRLMSLEVAMRWTSSSTENIYTFANTINTHEGGTHEEGFRAALTS
Tropheryma whipplei Bcu26          GLFDYVMYQNRIRKHEPLHEEIIHFQAENKDRLMSLEVAMRWTSSSTENIYTFANTINTHEGGTHEEGFRAALTS
Tropheryma whipplei Dig10          GLFDYVMYQNRIRKHEPLHEEIIHFQAENKDRLMSLEVAMRWTSSSTENIYTFANTINTHEGGTHEEGFRAALTS
Tropheryma whipplei Neuro20        GLFDYVMYQNRIRKHEPLHEEIIHFQAENKDRLMSLEVAMRWTSSSTENIYTFANTINTHEGGTHEEGFRAALTS
Tropheryma whipplei Neuro1         GLFDYVMYQNRIRKHEPLHEEIIHFQAENKDRLMSLEVAMRWTSSSTENIYTFANTINTHEGGTHEEGFRAALTS
Tropheryma whipplei Twist          GLFDYVMYQNRIRKHEPLHEEIIHFQAENKDRLMSLEVAMRWTSSSTENIYTFANTINTHEGGTHEEGFRAALTS
Tropheryma whipplei slow2          GLFDYVMYQNRIRKHEPLHEEIIHFQAENKDRLMSLEVAMRWTSSSTENIYTFANTINTHEGGTHEEGFRAALTS
Tropheryma whipplei Sali28         GLFDYVMYQNRIRKHEPLHEEIIHFQAENKDRLMSLEVAMRWTSSSTENIYTFANTINTHEGGTHEEGFRAALTS
Tropheryma whipplei DigMusc17      GLFDYVMYQNRIRKHEPLHEEIIHFQAENKDRLMSLEVAMRWTSSSTENIYTFANTINTHEGGTHEEGFRAALTS
Tropheryma whipplei Endo27         GLFDYVMYQNRIRKHEPLHEEIIHFQAENKDRLMSLEVAMRWTSSSTENIYTFANTINTHEGGTHEEGFRAALTS
Tropheryma whipplei DigADP25       GLFDYVMYQNRIRKHEPLHEEIIHFQAENKDRLMSLEVAMRWTSSSTENIYTFANTINTHEGGTHEEGFRAALTS
Tropheryma whipplei Dig7           GLFDYVMYQNRIRKHEPLHEEIIHFQAENKDRLMSLEVAMRWTSSSTENIYTFANTINTHEGGTHEEGFRAALTS
Tropheryma whipplei Art29          GLFDYVMYQNRIRKHEPLHEEIIHFQAENKDRLMSLEVAMRWTSSSTENIYTFANTINTHEGGTHEEGFRAALTS
Tropheryma whipplei Pneumo30       GLFDYVMYQNRIRKHEPLHEEIIHFQAENKDRLMSLEVAMRWTSSSTENIYTFANTINTHEGGTHEEGFRAALTS
Tropheryma whipplei Endo32         GLFDYVMYQNRIRKHEPLHEEIIHFQAENKDRLMSLEVAMRWTSSSTENIYTFANTINTHEGGTHEEGFRAALTS
Tropheryma whipplei shenzhen2      GLFDYVMYQNRIRKHEPLHEEIIHFQAENKDRLMSLEVAMRWTSSSTENIYTFANTINTHEGGTHEEGFRAALTS
Tropheryma whipplei shenzhen1      GLFDYVMYQNRIRKHEPLHEEIIHFQAENKDRLMSLEVAMRWTSSSTENIYTFANTINTHEGGTHEEGFRAALTS
```

```
                                     310       320       330       340       350       360       370
Tropheryma whipplei Neuro14    LVNRYAREKGFLKEKDESLQGEDIREGLTAIISVKLGEPQFEGQTKTKLGNTEMRGYVHHEVNSYLWDWFDSHPA
Tropheryma whipplei Dig9       LVNRYAREKGFLKEKDESLQGEDIREGLTAIISVKLGEPQFEGQTKTKLGNTEMRGYVHHEVNSYLWDWFDSHPA
Tropheryma whipplei Dig15      LVNRYAREKGFLKEKDESLQGEDIREGLTAIISVKLGEPQFEGQTKTKLGNTEMRGYVHHEVNSYLWDWFDSHPA
Tropheryma whipplei Art1       LVNRYAREKGFLKEKDESLQGEDIREGLTAIISVKLGEPQFEGQTKTKLGNTEMRGYVHHEVNSYLWDWFDSHPA
Tropheryma whipplei TW08/27    LVNRYAREKGFLKEKDESLQGEDIREGLTAIISVKLGEPQFEGQTKTKLGNTEMRGYVHHEVNSYLWDWFDSHPA
Tropheryma whipplei Bcu26      LVNRYAREKGFLKEKDESLQGEDIREGLTAIISVKLGEPQFEGQTKTKLGNTEMRGYVHHEVNSYLWDWFDSHPA
Tropheryma whipplei Dig10      LVNRYAREKGFLKEKDESLQGEDIREGLTAIISVKLGEPQFEGQTKTKLGNTEMRGYVHHEVNSYLWDWFDSHPA
Tropheryma whipplei Neuro20    LVNRYAREKGFLKEKDESLQGEDIREGLTAIISVKLGEPQFEGQTKTKLGNTEMRGYVHHEVNSYLWDWFDSHPA
Tropheryma whipplei Neuro1     LVNRYAREKGFLKEKDESLQGEDIREGLTAIISVKLGEPQFEGQTKTKLGNTEMRGYVHHEVNSYLWDWFDSHPA
Tropheryma whipplei Twist      LVNRYAREKGFLKEKDESLQGEDIREGLTAIISVKLGEPQFEGQTKTKLGNTEMRGYVHHEVNSYLWDWFDSHPA
Tropheryma whipplei slow2      LVNRYAREKGFLKEKDESLQGEDIREGLTAIISVKLGEPQFEGQTKTKLGNTEMRGYVHHEVNSYLWDWFDSHPA
Tropheryma whipplei Sali28     LVNRYAREKGFLKEKDESLQGEDVREGLTAIISVKLGEPQFEGQTKTKLGNTEMRGYVHHEVNSYLWDWFDSHPA
Tropheryma whipplei DigMusc17  LVNRYAREKGFLKEKDESLQGEDVREGLTAIISVKLGEPQFEGQTKTKLGNTEMRGYVHHEVNSYLWDWFDSHPA
Tropheryma whipplei Endo27     LVNRYAREKGFLKEKDESLQGEDVREGLTAIISVKLGEPQFEGQTKTKLGNTEMRGYVHHEVNSYLWDWFDSHPA
Tropheryma whipplei DigADP25   LVNRYAREKGFLKEKDESLQGEDVREGLTAIISVKLGEPQFEGQTKTKLGNTEMRGYVHHEVNSYLWDWFDSHPA
Tropheryma whipplei Dig7       LVNRYAREKGFLKEKDESLQGEDVREGLTAIISVKLGEPQFEGQTKTKLGNTEMRGYVHHEVNSYLWDWFDSHPA
Tropheryma whipplei Art29      LVNRYAREKGFLKEKDESLQGEDVREGLTAIISVKLGEPQFEGQTKTKLGNTEMRGYVHHEVNSYLWDWFDSHPA
Tropheryma whipplei Pneumo30   LVNRYAREKGFLKEKDESLQGEDVREGLTAIISVKLGEPQFEGQTKTKLGNTEMRGYVHHEVNSYLWDWFDSHPA
Tropheryma whipplei Endo32     LVNRYAREKGFLKEKDESLQGEDVREGLTAIISVKLGEPQFEGQTKTKLGNTEMRGYVHHEVNSYLWDWFDSHPA
Tropheryma whipplei shenzhen2  LVNRYAREKGFLKEKDESLQGEDVREGLTAIISVKLGEPQFEGQTKTKLGNTEMRGYVHHEVNSYLWDWFDSHPA
Tropheryma whipplei shenzhen1  LVNRYAREKGFLKEKDESLQGEDVREGLTAIISVKLGEPQFEGQTKTKLGNTEMRGYVHHEVNSYLWDWFDSHPA
```

```
                                   380       390       400       410       420       430       440       450
Tropheryma whipplei Neuro14    QAKVIISKALLAAEARLAAKRAREASRKRGLFESVSVPGKLKECQAKDPAVSELFIVEGDSAGGSAIQGRDPLTQ
Tropheryma whipplei Dig9       QAKVIISKALLAAEARLAAKRAREASRKRGLFESVSVPGKLKECQAKDPAVSELFIVEGDSAGGSAIQGRDPLTQ
Tropheryma whipplei Dig15      QAKVIISKALLAAEARLAAKRAREASRKRGLFESVSVPGKLKECQAKDPAVSELFIVEGDSAGGSAIQGRDPLTQ
Tropheryma whipplei Art1       QAKVIISKALLAAEARLAAKRAREASRKRGLFESVSVPGKLKECQAKDPAVSELFIVEGDSAGGSAIQGRDPLTQ
Tropheryma whipplei TW08/27    QAKVIISKALLAAEARLAAKRAREASRKRGLFESVSMPGKLKECQAKDPAVSELFIVEGDSAGGSAIQGRDPLTQ
Tropheryma whipplei Bcu26      QAKVIISKALLAAEARLAAKRAREASRKRGLFESVSMPGKLKECQAKDPAVSELFIVEGDSAGGSAIQGRDPLTQ
Tropheryma whipplei Dig10      QAKVIISKALLAAEARLAAKRAREASRKRGLFESVSMPGKLKECQAKDPAVSELFIVEGDSAGGSAIQGRDPLTQ
Tropheryma whipplei Neuro20    QAKVIISKALLAAEARLAAKRAREASRKRGLFESVSMPGKLKECQAKDPAVSELFIVEGDSAGGSAIQGRDPLTQ
Tropheryma whipplei Neuro1     QAKVIISKALLAAEARLAAKRAREASRKRGLFESVSMPGKLKECQAKDPAVSELFIVEGDSAGGSAIQGRDPLTQ
Tropheryma whipplei Twist      QAKVIISKALLAAEARLAAKRAREASRKRGLFESVSMPGKLKECQAKDPAVSELFIVEGDSAGGSAIQGRDPLTQ
Tropheryma whipplei slow2      QAKVIISKALLAAEARLAAKRAREASRKRGLFESVSMPGKLKECQAKDPAVSELFIVEGDSAGGSAIQGRDPLTQ
Tropheryma whipplei Sali28     QAKVIISKALLAAEARLAAKRAREASRKRGLFESVSMPGKLKECQAKDPAVSELFIVEGDSAGGSAIQGRDPLTQ
Tropheryma whipplei DigMusc17  QAKVIISKALLAAEARLAAKRAREASRKRGLFESVSMPGKLKECQAKDPAVSELFIVEGDSAGGSAIQGRDPLTQ
Tropheryma whipplei Endo27     QAKVIISKALLAAEARLAAKRAREASRKRGLFESVSMPGKLKECQAKDPAVSELFIVEGDSAGGSAIQGRDPLTQ
Tropheryma whipplei DigADP25   QAKVIISKALLAAEARLAAKRAREASRKRGLFESVSMPGKLKECQAKDPAVSELFIVEGDSAGGSAIQGRDPLTQ
Tropheryma whipplei Dig7       QAKVIISKALLAAEARLAAKRAREASRKRGLFESVSMPGKLKECQAKDPAVSELFIVEGDSAGGSAIQGRDPLTQ
Tropheryma whipplei Art29      QAKVIISKALLAAEARLAAKRAREASRKRGLFESVSMPGKLKECQAKDPAVSELFIVEGDSAGGSAIQGRDPLTQ
Tropheryma whipplei Pneumo30   QAKVIISKALLAAEARLAAKRAREASRKRGLFESVSMPGKLKECQAKDPAVSELFIVEGDSAGGSAIQGRDPLTQ
Tropheryma whipplei Endo32     QAKVIISKALLAAEARLAAKRAREASRKRGLFESVSMPGKLKECQAKDPAVSELFIVEGDSAGGSAIQGRDPLTQ
Tropheryma whipplei shenzhen2  QAKVIISKALLAAEARLAAKRAREASRKRGLFESVSMPGKLKECQAKDPAVSELFIVEGDSAGGSAIQGRDPLTQ
Tropheryma whipplei shenzhen1  QAKVIISKALLAAEARLAAKRAREASRKRGLFESVSMPGKLKECQAKDPAVSELFIVEGDSAGGSAIQGRDPLTQ
```

```
                                         460       470       480       490       500       510       520
Tropheryma whipplei Neuro14    AVLPLRGKILNVEKARLDKAIANSEIQAMLTAFGSGIAEDFDESKARYHKIILMSDADVDGQHITTLLLTLLFRY
Tropheryma whipplei Dig9       AVLPLRGKILNVEKARLDKAIANSEIQAMLTAFGSGIAEDFDESKARYHKIILMSDADVDGQHITTLLLTLLFRY
Tropheryma whipplei Dig15      AVLPLRGKILNVEKARLDKAIANSEIQAMLTAFGSGIAEDFDESKARYHKIILMSDADVDGQHITTLLLTLLFRY
Tropheryma whipplei Art1       AVLPLRGKILNVEKARLDKAIANSEIQAMLTAFGSGIAEDFDESKARYHKIILMSDADVDGQHITTLLLTLLFRY
Tropheryma whipplei TW08/27    AVLPLRGKILNVEKARLDKAIANSEIQAMLTAFGSGIAEDFDESKARYHKIILMSDADVDGQHITTLLLTLLFRY
Tropheryma whipplei Bcu26      AVLPLRGKILNVEKARLDKAIANSEIQAMLTAFGSGIAEDFDESKARYHKIILMSDADVDGQHITTLLLTLLFRY
Tropheryma whipplei Dig10      AVLPLRGKILNVEKARLDKAIANSEIQAMLTAFGSGIAEDFDESKARYHKIILMSDADVDGQHITTLLLTLLFRY
Tropheryma whipplei Neuro20    AVLPLRGKILNVEKARLDKAIANSEIQAMLTAFGSGIAEDFDESKARYHKIILMSDADVDGQHITTLLLTLLFRY
Tropheryma whipplei Neuro1     AVLPLRGKILNVEKARLDKAIANSEIQAMLTAFGSGIAEDFDESKARYHKIILMSDADVDGQHITTLLLTLLFRY
Tropheryma whipplei Twist      AVLPLRGKILNVEKARLDKAIANSEIQAMLTAFGSGIAEDFDESKARYHKIILMSDADVDGQHITTLLLTLLFRY
Tropheryma whipplei slow2      AVLPLRGKILNVEKARLDKAIANSEIQAMLTAFGSGIAEDFDESKARYHKIILMSDADVDGQHITTLLLTLLFRY
Tropheryma whipplei Sali28     AVLPLRGKILNVEKARLDKAIANSEIQAMLTAFGSGIAEDFDESKARYHKIILMSDADVDGQHITTLLLTLLFRY
Tropheryma whipplei DigMusc17  AVLPLRGKILNVEKARLDKAIANSEIQAMLTAFGSGIAEDFDESKARYHKIILMSDADVDGQHITTLLLTLLFRY
Tropheryma whipplei Endo27     AVLPLRGKILNVEKARLDKAIANSEIQAMLTAFGSGIAEDFDESKARYHKIILMSDADVDGQHITTLLLTLLFRY
Tropheryma whipplei DigADP25   AVLPLRGKILNVEKARLDKAIANSEIQAMLTAFGSGIAEDFDESKARYHKIILMSDADVDGQHITTLLLTLLFRY
Tropheryma whipplei Dig7       AVLPLRGKILNVEKARLDKAIANSEIQAMLTAFGSGIAEDFDESKARYHKIILMSDADVDGQHITTLLLTLLFRY
Tropheryma whipplei Art29      AVLPLRGKILNVEKARLDKAIANSEIQAMLTAFGSGIAEDFDESKARYHKIILMSDADVDGQHITTLLLTLLFRY
Tropheryma whipplei Pneumo30   AVLPLRGKILNVEKARLDKAIANSEIQAMLTAFGSGIAEDFDESKARYHKIILMSDADVDGQHITTLLLTLLFRY
Tropheryma whipplei Endo32     AVLPLRGKILNVEKARLDKAIANSEIQAMLTAFGSGIAEDFDESKARYHKIILMSDADVDGQHITTLLLTLLFRY
Tropheryma whipplei shenzhen2  AVLPLRGKILNVEKARLDKAIANSEIQAMLTAFGSGIAEDFDESKARYHKIILMSDADVDGQHITTLLLTLLFRY
Tropheryma whipplei shenzhen1  AVLPLRGKILNVEKARLDKAIANSEIQAMLTAFGSGIAEDFDESKARYHKIILMSDADVDGQHITTLLLTLLFRY
```

Quinolone Resistance-Determining Regions

```
                                   530       540       550       560       570       580       590       600
Tropheryma whipplei Neuro14    MRPLIESGYVYLAHPPLYRIKWSNSEHDYVFSDRERDKKLAEGKLSGKKIPKENAIQRYKGLGEMNYRELWDTTM
Tropheryma whipplei Dig9       MRPLIESGYVYLAHPPLYRIKWSNSEHDYVFSDRERDKKLAEGKLSGKKIPKENAIQRYKGLGEMNYRELWDTTM
Tropheryma whipplei Dig15      MRPLIESGYVYLAHPPLYRIKWSNSEHDYVFSDRERDKKLAEGKLSGKKIPKENAIQRYKGLGEMNYRELWDTTM
Tropheryma whipplei Art1       MRPLIESGYVYLAHPPLYRIKWSNSEHDYVFSDRERDKKLAEGKLSGKKIPKENAIQRYKGLGEMNYRELWDTTM
Tropheryma whipplei TW08/27    MRPLIESGYVYLAHPPLYRIKWSNSEHDYVFSDRERDKKLAEGKLSGKKIPKENAIQRYKGLGEMNYRELWDTTM
Tropheryma whipplei Bcu26      MRPLIESGYVYLAHPPLYRIKWSNSEHDYVFSDRERDKKLAEGKLSGKKIPKENAIQRYKGLGEMNYRELWDTTM
Tropheryma whipplei Dig10      MRPLIESGYVYLAHPPLYRIKWSNSEHDYVFSDRERDKKLAEGKLSGKKIPKENAIQRYKGLGEMNYRELWDTTM
Tropheryma whipplei Neuro20    MRPLIESGYVYLAHPPLYRIKWSNSEHDYVFSDRERDKKLAEGKLSGKKIPKENAIQRYKGLGEMNYRELWDTTM
Tropheryma whipplei Neuro1     MRPLIESGYVYLAHPPLYRIKWSNSEHDYVFSDRERDKKLAEGKLSGKKIPKENAIQRYKGLGEMNYRELWDTTM
Tropheryma whipplei Twist      MRPLIESGYVYLAHPPLYRIKWSNSEHDYVFSDRERDKKLAEGKLSGKKIPKENAIQRYKGLGEMNYRELWDTTM
Tropheryma whipplei slow2      MRPLIESGYVYLAHPPLYRIKWSNSEHDYVFSDRERDKKLAEGKLSGKKIPKENAIQRYKGLGEMNYRELWDTTM
Tropheryma whipplei Sali28     MRPLIESGYVYLAHPPLYRIKWSNSEHDYVFSDRERDKKLAEGKLSGKKIPKENAIQRYKGLGEMNYRELWDTTM
Tropheryma whipplei DigMusc17  MRPLIESGYVYLAHPPLYRIKWSNSEHDYVFSDRERDKKLAEGKLSGKKIPKENAIQRYKGLGEMNYRELWDTTM
Tropheryma whipplei Endo27     MRPLIESGYVYLAHPPLYRIKWSNSEHDYVFSDRERDKKLAEGKLSGKKIPKENAIQRYKGLGEMNYRELWDTTM
Tropheryma whipplei DigADP25   MRPLIESGYVYLAHPPLYRIKWSNSEHDYVFSDRERDKKLAEGKLSGKKIPKENAIQRYKGLGEMNYRELWDTTM
Tropheryma whipplei Dig7       MRPLIESGYVYLAHPPLYRIKWSNSEHDYVFSDRERDKKLAEGKLSGKKIPKENAIQRYKGLGEMNYRELWDTTM
Tropheryma whipplei Art29      MRPLIESGYVYLAHPPLYRIKWSNSEHDYVFSDRERDKKLAEGKLSGKKIPKENAIQRYKGLGEMNYRELWDTTM
Tropheryma whipplei Pneumo30   MRPLIESGYVYLAHPPLYRIKWSNSEHDYVFSDRERDKKLAEGKLSGKKIPKENAIQRYKGLGEMNYRELWDTTM
Tropheryma whipplei Endo32     MRPLIESGYVYLAHPPLYRIKWSNSEHDYVFSDRERDKKLAEGKLSGKKIPKENAIQRYKGLGEMNYRELWDTTM
Tropheryma whipplei shenzhen2  MRPLIESGYVYLAHPPLYRIKWSNSEHDYVFSDRERDKKLAEGKLSGKKIPKENAIQRYKGLGEMNYRELWDTTM
Tropheryma whipplei shenzhen1  MRPLIESGYVYLAHPPLYRIKWSNSEHDYVFSDRERDKKLAEGKLSGKKIPKENAIQRYKGLGEMNYRELWDTTM
```

```
                                            610       620       630       640
                                              .         .         .         .
Tropheryma whipplei Neuro14    NPATRTLRQITVDDVTLADEIFSVLMGEDVDSRRKFIQHNARDVRFLDI
Tropheryma whipplei Dig9       NPATRTLRQITVDDVTLADEIFSVLMGEDVDSRRKFIQHNARDVRFLDI
Tropheryma whipplei Dig15      NPATRTLRQITVDDVTLADEIFSVLMGEDVDSRRKFIQHNARDVRFLDI
Tropheryma whipplei Art1       NPATRTLRQITVDDVTLADEIFSVLMGEDVDSRRKFIQHNARDVRFLDI
Tropheryma whipplei TW08/27    NPATRTLRQITVDDVTLADEIFSVLMGEDVDSRRKFIQHNARDVRFLDI
Tropheryma whipplei Bcu26      NPATRTLRQITVDDVTLADEIFSVLMGEDVDSRRKFIQHNARDVRFLDI
Tropheryma whipplei Dig10      NPATRTLRQITVDDVTLADEIFSVLMGEDVDSRRKFIQHNARDVRFLDI
Tropheryma whipplei Neuro20    NPATRTLRQITVDDVTLADEIFSVLMGEDVDSRRKFIQHNARDVRFLDI
Tropheryma whipplei Neuro1     NPATRTLRQITVDDVTLADEIFSVLMGEDVDSRRKFIQHNARDVRFLDI
Tropheryma whipplei Twist      NPATRTLRQITVDDVTLADEIFSVLMGEDVDSRRKFIQHNARDVRFLDI
Tropheryma whipplei slow2      NPATRTLRQITVDDVTLADEIFSVLMGEDVDSRRKFIQHNARDVRFLDI
Tropheryma whipplei Sali28     NPATRTLRQITVDDVTLADEIFSVLMGEDVDSRRKFIQHNARDVRFLDI
Tropheryma whipplei DigMusc17  NPATRTLRQITVDDVTLADEIFSVLMGEDVDSRRKFIQHNARDVRFLDI
Tropheryma whipplei Endo27     NPATRTLRQITVDDVTLADEIFSVLMGEDVDSRRKFIQHNARDVRFLDI
Tropheryma whipplei DigADP25   NPATRTLRQITVDDVTLADEIFSVLMGEDVDSRRKFIQHNARDVRFLDI
Tropheryma whipplei Dig7       NPATRTLRQITVDDVTLADEIFSVLMGEDVDSRRKFIQHNARDVRFLDI
Tropheryma whipplei Art29      NPATRTLRQITVDDVTLADEIFSVLMGEDVDSRRKFIQHNARDVRFLDI
Tropheryma whipplei Pneumo30   NPATRTLRQITVDDVTLADEIFSVLMGEDVDSRRKFIQHNARDVRFLDI
Tropheryma whipplei Endo32     NPATRTLRQITVDDVTLADEIFSVLMGEDVDSRRKFIQHNARDVRFLDI
Tropheryma whipplei shenzhen2  NPATRTLRQITVDDVTLADEIFSVLMGEDVDSRRKFIQHNARDVRFLDI
Tropheryma whipplei shenzhen1  NPATRTLRQITVDDVTLADEIFSVLMGEDVDSRRKFIQHNARDVRFLDI
```
